# Assessing Animal Welfare Impacts in the Management of European Rabbits (*Oryctolagus cuniculus*), European Moles (*Talpa europaea*) and Carrion Crows (*Corvus corone*)

**DOI:** 10.1371/journal.pone.0146298

**Published:** 2016-01-04

**Authors:** Sandra E. Baker, Trudy M. Sharp, David W. Macdonald

**Affiliations:** 1 Wildlife Conservation Research Unit, Department of Zoology, University of Oxford, Oxford, Oxfordshire, United Kingdom; 2 Fowlers Gap Arid Zone Research Station, Centre of Ecosystem Science, School of Biological, Earth and Environmental Sciences, University of New South Wales, Kensington, NSW 2052, Australia; Harvard University Faculty of Arts and Sciences, UNITED STATES

## Abstract

Human-wildlife conflict is a global issue. Attempts to manage this conflict impact upon wild animal welfare, an issue receiving little attention until relatively recently. Where human activities harm animal welfare these effects should be minimised where possible. However, little is known about the welfare impacts of different wildlife management interventions, and opinions on impacts vary widely. Welfare impacts therefore need to be assessed objectively. Our objectives were to: 1) establish whether an existing welfare assessment model could differentiate and rank the impacts of different wildlife management interventions (for decision-making purposes); 2) identify and evaluate any additional benefits of making formal welfare assessments; and 3) illustrate issues raised by application of the model. We applied the welfare assessment model to interventions commonly used with rabbits *(Oryctolagus cuniculus)*, moles *(Talpa europaea)* and crows *(Corvus corone)* in the UK. The model ranked interventions for rabbits (least impact first: fencing, head shot, chest shot) and crows (shooting, scaring, live trapping with cervical dislocation). For moles, managing molehills and tunnels scored least impact. Both spring trapping, and live trapping followed by translocation, scored greater impacts, but these could not be compared directly as they scored on different axes of the model. Some rankings appeared counter-intuitive, highlighting the need for objective formal welfare assessments. As well as ranking the humaneness of interventions, the model highlighted future research needs and how Standard Operating Procedures might be improved. The model is a milestone in assessing wildlife management welfare impacts, but our research revealed some limitations of the model and we discuss likely challenges in resolving these. In future, the model might be developed to improve its utility, e.g. by refining the time-scales. It might also be used to reach consensus among stakeholders about relative welfare impacts or to identify ways of improving wildlife management practice in the field.

## Introduction

Human-wildlife conflict is a pervasive global issue [1], with diverse causes [2] (e.g. competition for food or other resources [3], damage to livestock [4] or property [5], threats to conservation [6] and risk of disease spread [7, 8], injury or death [2, 9]), and it occurs in a wide variety of circumstances (agriculture, game-rearing, fisheries, forestry, food processing, amenities, business and domestic settings [e.g. [10–12]). As well as threatening livelihoods, conservation and human lives, human-wildlife conflict costs many millions of US dollars per annum globally, e.g. annual livestock losses through depredation alone have been estimated at US$171 million in South Africa [13], while livestock losses to coyotes, in the USA, cost US$40 million [14]. As the global human population continues to grow and people and wildlife are forced into closer proximity, human-wildlife conflicts [15, 16] and human impacts on wild animals [17] are set to increase.

Typically, people try to manage human-wildlife conflict, and there may even be a legal obligation on a land owner or manager to kill, or otherwise control, certain species (e.g., in Britain, rabbits, hares (*Lepus europaeus*), rats (*Rattus norvegicus*), mice (*Mus musculus*), deer (e.g. *Dama dama*, *Capreolus capreolus*), foxes (*Vulpes vulpes*), moles and certain birds, under the Pests Act 1954 [18], the Prevention of Damage by Pests Act 1949 [19] or the Agriculture Act 1947 [20]). While lethal and non-lethal methods are available (their suitability depending on the species and circumstances concerned), some managers may rely heavily on lethal methods (e.g. shooting, snaring, poisoning, gassing, spring trapping [spring traps are lethal traps powered by a spring]) [21], especially in the management of carnivores [2], where there may be an element of retaliation involved if livestock have been killed [22]. Sometimes, where a species is perceived as living at high density, lethal control may be a matter of tradition or conducted prophylactically, or simply routinely, e.g. some mole catching in Britain [23]. Many non-lethal methods are available, with some having been used for centuries, e.g. scarecrows [24]. Some non-lethal methods are widely used (e.g. habitat management, fencing, tree-guards, scaring devices), while some are used in particular scenarios (e.g. fertility control, diversionary feeding, translocation), and others (e.g. learned food aversions) require further development [21, 25]. Whether lethal or non-lethal methods are used, wildlife management clearly has the capacity to impact upon the welfare of target (and non-target) animals [26]. Where people adversely affect the welfare of animals there is an ethical obligation to minimise this impact where possible, and it is important to consider seriously whether control is necessary and, if so, to use the most humane option available that achieves the desired aims, whether lethal or non-lethal [27]. Humane, in this sense, and the sense used throughout this paper, means that pain, suffering and distress is avoided or minimised [28]. It is important however to note that ‘humane’ is sometimes used by other authors, or in general use in different cultures, to convey other concepts e.g. ethical, or non-lethal (e.g. see ‘humane mole tube traps’ in Baker et al [29]).

The importance of animal welfare is gathering recognition internationally [30]. However, until the second half of the 20th century, relatively little attention was paid to anthropogenic impacts on the welfare of wild animals, compared to domestic and laboratory animals [31]. In the 1950s and 60s spring trap regulation was introduced and certain traps and poisons were banned in the UK. In the 1980s and 1990s a number of papers were published on the welfare impacts of traps on wildlife, e.g. by Warburton and Zelin and their respective colleagues [32, 33], and International Organization for Standardization (ISO) trap standards were developed [34, 35]. Recent studies have addressed wild animal welfare considerations in wildlife reintroductions [36], the wildlife trade [37], the exotic pet trade [38], and wildlife tourist attractions [39]. Nevertheless, even in the 21^st^ century, the welfare of wild animals continues to lag behind, with even animal welfare scientists paying the issue little attention until recently [40]. Indeed, in 2008, David Fraser used wolves, pigs and dogs as examples, to contrast the way, even now, people in Western cultures treat wild, farmed and pet animals with similar capacities for suffering [41]. The welfare of pest animals has been particularly undervalued [15], but now respect for wild animal welfare is growing rapidly [2, 15, 42, 43] and there is increasing concern, among the wider community in some parts of the world, regarding the acceptability of killing wild animals considered pests [2, 16, 42].

Legislative changes also reflect greater attention to wild animal welfare. For example, in the late 1990s, Europe signed the Agreement on International Humane Trapping Standards with Canada, Russia and the United States of America, which banned the import of wild fur products unless they come from countries where leg hold traps are prohibited and where trapping methods meet internationally-agreed welfare standards. Also, wild animals in Britain have recently been granted wider protection through the Wild Mammals Protection Act 1996 [44], the Protection of Wild Mammals (Scotland) Act 2002 [45], the Hunting Act 2004 [46], the Animal Welfare Act 2006 [47] and the Animal Health and Welfare (Scotland) Act 2006 [48]. Under the Wild Mammals Protection Act it is an offence, except under certain exemptions, to mutilate, kick, beat, nail or otherwise impale, stab, burn, stone, crush, drown, drag or asphyxiate any wild mammal with intent to inflict unnecessary suffering [44]. The Animal Welfare Act introduces a general duty of care for all animals under the control of man, including wild vertebrates held captive or restrained by man’s actions, e.g., caught in a net or snare, held in the hand, in an enclosure, pen or cage trap or during transportation [49].

Selection of a wildlife management method may be influenced by many factors, including its perceived humaneness, efficacy, cost-effectiveness, target specificity, practicality, ease of application, speed of effect, durability, legality (e.g. statutory restrictions/requirements), safety for operators and other people, environmental impact, acceptability to the public and, to some extent, how tolerable any conflict is perceived to be. If the management has another aim, this may be an additional consideration, e.g. if the animal is to be harvested for fur then it might need to be live-trapped and killed followed by immediate skinning. Humaneness should (and is increasingly likely to) be taken into account in deciding whether and how wild animals are managed [2, 43]. Littin et al. [27] propose that, as well as using the most humane management methods that achieve the aims in any given situation, people should aim to maximise the humaneness of existing methods and to identify new methods that are more humane. However, there is little reliable information available with which to evaluate and compare the humaneness of wildlife management methods [42], and the opinions of wildlife managers on the humaneness of a management method may vary widely, for example, depending on which stakeholder group the manager belongs to and whether they believe they have suffered losses to the species in question [4, 50]. This may be related to the fact that the perceived importance of humaneness in choosing a management method also varies with stakeholder group. There is therefore a clear need for objective assessment of the welfare impacts of different methods, so that humaneness may be considered without bias when deciding on a course of wildlife management action [27, 42].

Assessing animal welfare is notoriously difficult [51–53], but various approaches have been used [41], and a large number of objective and semi-objective measures proposed (e.g., physiological and behavioural [54]), for determining the welfare state of an animal [30]. However, a key problem in assessing animal welfare can be that interpretation of the many proposed objective welfare measures requires some subjective judgement [42], which may be influenced by the assessor’s regard for the animal involved [52]. Also, no single measure is likely to represent adequately an animal’s welfare status and there are potential problems associated with combining separate indicators to estimate overall welfare impact. In 1979, The Farm Animal Welfare Council was charged with advising the UK government on farm animal welfare and produced its Five Freedoms to define the ideal physical and mental states for an animal [55]. Today these are expressed as: 1. freedom from hunger and thirst; 2. freedom from discomfort; 3. freedom from pain, injury or disease; 4. freedom to express normal behaviour; and 5. freedom from fear and distress [56]. In 1994, Mellor and Reid developed, from the Five Freedoms, five ‘domains of potential [welfare] compromise’ to produce a system for assessing the welfare impacts of a proposed animal experiment or usage [57]. This, and a revised version, have been used in New Zealand since 1997 to assess and record the level of animal welfare compromise imposed by research, testing and teaching [42, 58]. Mellor and Reid’s five welfare domains consider impacts on an animal’s nutrition, environment, health, behaviour and mental state. Their system was designed to be comprehensive, aiming to capture all possible welfare impacts, such that the welfare impact outcome identified depends on all five domains, without the need to combine or weight scores in any way (which could create unplanned biases) [53]. Their system has been adapted for use with farm animals [59] and pests [60, 61], and incorporated into the Zoo and Aquarium Association’s animal welfare position statement [62]; the five domains model and other systems devised for assessing farm animal welfare are reviewed by Botreau et al. [63, 64]. In 1994, Kirkwood and co-authors examined ways of assessing adverse human impacts on the welfare of free-living wild animals [31]. They proposed that the welfare impact of human interventions could be evaluated by considering: 1) the nature of the harm caused; 2) the duration of the harm; 3) the numbers of animals affected; and 4) their capacity for suffering. Then, in 1999, Broom suggested that an estimate of the severity of a pest management procedure could be obtained by multiplying the extent of poor welfare by its duration [65].

In 2008, Sharp and Saunders developed a model for assessing the relative humaneness of pest animal management interventions in Australia [42, 66]. This has been used to assess the welfare impact of various management interventions on a range of wild vertebrates in Australia and New Zealand [26, 42]. The model allows for the assessment of a wide range of both lethal and non-lethal management interventions. It provides a systematic, comprehensive and transparent process designed to promote consensus among stakeholders about the humaneness of management interventions. Evidence-based assessments are made using information from the literature regarding the animal’s physiological, behavioural and pathological responses to a particular intervention. Assessments involve thorough literature searching and the documentation of evidence. Sharp and Saunders developed their model in collaboration with stakeholders, with particular expertise in animal welfare and pest management, including farming groups, animal welfare organisations, government and non-government land managers and the community. The authors reported that the final model received widespread support from stakeholders, with most considering it effective and practical [42].

To demonstrate the use of the Sharp and Saunders’ model (‘the model’), we assessed the relative welfare impacts (on target animals) for three commonly used interventions for managing each of European rabbits, European moles and carrion crows. These species were chosen as they are widely regarded as pests in Britain [67–69] and elsewhere in Europe [70–72], a range of options for their management are available and data were likely to be available for use with the model. Our objectives were to: 1) determine whether the model could be used to differentiate and rank, systematically, the impacts of different wildlife management interventions used with each species (thereby providing a basis for decision-making); 2) demonstrate the value of making formalised welfare assessments in identifying interventions, or components of interventions, that pose a relatively greater welfare threat, or which are currently under-researched and poorly understood; and 3) to identify issues raised by application of the model. These objectives were achieved.

## Materials and Methods

### Standard operating procedures (SOPs)

The humaneness of wildlife management depends on which intervention is used, how it is applied and the skill of the operator [42]. Variation in details, such as doses, frequency of trap inspection, speed and skill in handling animals, confirmation of death etc, may have a significant effect on the level and duration of any welfare impact and so the exact details of a management intervention are crucial in determining its welfare and other outcomes. A welfare assessment therefore needs to apply to a particular standard operating procedure (SOP) that makes explicit the exact methodology of the intervention. Meaningful comparison of the welfare impacts of different management interventions is only possible if SOPs are followed. As well as providing the basis for making welfare assessments, a SOP allows uniform implementation of ‘best practice’ wildlife management, and management skills training, and by standardising the details of a management intervention, welfare impacts may be reduced or prevented [42].

Before developing their welfare assessment model (‘the model’), Sharp and Saunders produced a series of SOPs covering various management interventions used with invasive species in Australia [73]). SOPs are generally not available for wildlife management interventions in Britain and so we developed British SOPs, covering three management interventions used with each of rabbits, moles and crows (these are listed in Table 1 and the full SOPs are available at S1–S8 SOPs). We followed the format of Sharp’s SOPs as a guide [73]. Our SOPs were designed to incorporate best practice as described in a variety of relevant sources, including Natural England Information Notes and Advice Notes, Codes of Practice (produced by the Department of Environment, Food and Rural Affairs (DEFRA), British Association for Shooting and Conservation (BASC), the Game and Wildlife Conservation Trust (GWCT) and the National Farmers Union (NFU)), Forestry Authority Practice Notes, Natural England Licenses, Australian government SOPs (produced by Sharp and Saunders), reports by the Ministry of Agriculture Fisheries and Food (MAFF) and by its successor, DEFRA, and by the Pesticides Safety Directorate (PSD), product manufacturers’ instructions and safety information, the scientific literature and books. These SOPs do not constitute advice and have been produced for the purposes of this study only.

**Table 1:** Details of the eight standard operating procedures created and the nine associated welfare assessments (both head and chest shots for rabbits are covered by a single SOP).

	Standard Operating Procedure (SOP)	Welfare assessment	Summary of main features
Rabbits	Shooting rabbits (S1 SOP)	Shooting rabbits; head shot	Head shot, specified rifle or shotgun (depending on distance), specified ammunition, lamp used if at night
		Shooting rabbits; chest shot	Chest shot, specified rifle or shotgun (depending on distance), specified ammunition, lamp used if at night
	Fencing rabbits from crops (S2 SOP)	*Fencing rabbits from crops (installation); Fencing rabbits from crops (established)	Permanent, non-electric, wire-mesh fencing, around wheat field, installed after harvest/before winter planting. Assessments for 2 month period at installation (Sept-Oct) and again once established (spring)
Moles	Spring trapping moles (S3 SOP)	Spring trapping moles	Scissor, Duffus or Talpa spring traps that meet welfare approval standards, during spring (but outside breeding period), checked every 24 hours
	Live trapping and translocation of moles (S4 SOP)	*Live trapping moles; Translocating moles	Plastic tube traps, with food (earthworms), no bedding, during spring (but outside breeding period), checked every 4 hours. Soft-release at suitable and apparently unoccupied sites, in man-made chambers with bedding and food, released during spring (but outside breeding period)
	Managing molehills and tunnels (S5 SOP)	Managing molehills and tunnels on lawns	Molehill soil carefully lifted using a shovel or spade, and redistributed elsewhere, surface tunnels gently trodden down
Crows	Shooting crows (S6 SOP)	Shooting crows	Chest shot; specified shotgun; specified ammunition; daylight hours only; outside breeding period; under Natural England General Licence WML-GL04
	Cage trapping and cervical dislocation of crows (S7 SOP)	Cage trapping with cervical dislocation of crows	Single-capture Larsen traps, checked every 24 hours; outside breeding period; under Natural England General Licence WML-GL04
	Scaring crows using gas guns (S8 SOP)	Scaring crows using gas guns	Propane or acetylene gas guns; daylight only; outside breeding period. Assessments for 2 month period between harvest and winter wheat planting (Sept-Oct)

*In two cases, above, we made two separate assessments: 1) where a method consisted of two non-lethal parts (live trapping moles followed by translocation); and 2) where a method consisted of two separable phases (installation of a rabbit fence in autumn, and once the fence was established the following spring).

### Assessments

The model comprises parts A and B. Part A is based on Mellor and Reid’s five welfare impact domains [57] and examines the impact of a lethal or non-lethal management intervention on an individual animal’s welfare, and the duration of this impact. For lethal interventions, Part A of the assessment examines only the impact before the action causing death, while for non-lethal interventions it examines the impact of the whole procedure. We refer to this as the ‘non-lethal welfare impact’ (although Sharp and Saunders called it the ‘overall welfare impact’ [42]). Part B of the model applies only to lethal interventions and is based on Broom’s suggested approach [65]. It assesses the impact on welfare of any killing method involved, by evaluating the intensity and duration of suffering caused by the killing technique alone. Therefore lethal interventions were scored under both Parts A and B, and non-lethal interventions under Part A only.

Part A impacts were considered under five separate domains: 1) water deprivation, food deprivation or malnutrition; 2) environmental challenge; 3) injury, disease, functional impairment; 4) behavioural, interactive restriction; 5) anxiety, fear, pain, distress, thirst, hunger etc. A welfare impact category (‘no impact’, ‘mild impact’, ‘moderate impact’, ‘severe impact’ or ‘extreme impact’) was assigned for each domain by referring to a set of impact scales for Part A (S1–S5 Tables). The impact categories assigned reflected the level of impact at the time of maximum impact. Where there was insufficient information to assign a single impact category, a range of categories was assigned, e.g. moderate to severe impact, and supporting references cited where appropriate. The score in domain 5 was assessed as the cumulative effect of the other four domains. This was usually equivalent to the maximum impact assigned to any of the domains 1–4, but could be greater. Ultimately the overall impact category for the management intervention was determined; this was usually the same as the category assigned to domain 5 (mental state), but if domain 5 could not be assessed for some reason then the overall impact category would be based on the level of welfare compromise under the other domains. Next, the duration of the impact (‘immediate/seconds’, ‘minutes’, ‘hours’, ‘days’, ‘weeks’) was determined. Then, the scoring matrix for Part A (S6 Table) was consulted, using the overall impact category, and the duration of that impact, to identify the non-lethal welfare impact of the intervention (ranging between 1 [no impact] and 8 [maximum impact]). Crucially, the scientific evidence for the impact scores assigned to the five domains was recorded as referenced notes.

To make an assessment under Part B, the time to insensibility from the application of the action causing death was determined (‘immediate/seconds’, ‘minutes’, ‘hours’, ‘days’, ‘weeks’). [Note, a lag time was deducted where there were no negative welfare impacts immediately, e.g. between setting a mole trap and trapping a mole.] Then the level of suffering (‘no suffering’, ‘mild suffering’, ‘moderate suffering’, ‘severe suffering’ or ‘extreme suffering’) was identified from the impact scale for Part B (S7 Table). Where a single impact category could not be assigned with confidence, a range of categories was allocated, e.g. mild to moderate suffering. Then the scoring matrix for Part B (S8 Table) was consulted, using the level of suffering and the time to insensibility, to identify the impact of the killing method (ranging between A [no impact] and H [maximum impact]). The evidence used to determine the level and duration of suffering was recorded as referenced notes. For lethal interventions, the scores for Parts A and B of the assessment were combined to give the overall assessment, ranging 1A-8H. Scores for non-lethal interventions ranged 1–8. Ultimately the welfare impact scores were compared for different management interventions applied to the same species.

When welfare assessments are made using the model for widespread use, this should be done by a panel of experts [42]. However, our purpose here was to demonstrate both the use of the model, and its associated benefits, and to identify issues raised by its application, and the assessments were made by SB with guidance from TS and DM. We assessed the management interventions listed in Table 1, assuming they were conducted according to best practice as specified in the relevant SOPs. Full details of the method for applying the model are available elsewhere [42] and are summarised here. Assessments were recorded in a standard format (e.g. S9 Table). Before beginning an assessment, any assumptions were made explicit, including that best practice was followed, according to a particular SOP. Where relevant, the timing of the management intervention was recorded, e.g. which season, or time of day, and, for those non-lethal interventions without a specific end-point, the duration of the intervention conducted (for scaring crows and fencing rabbits this was assumed to be two months). Other key details, such as trap-checking frequencies, were also recorded. Comments (relating, for example, to impacts on dependent offspring or other non-target animals, or what the effect might be if the operator deviated from the SOP in a particular way etc) were noted at the end of an assessment. Each assessment related to the welfare impact of a management intervention on a single target animal. Assessments were based on objective evidence from the literature and where necessary this was extrapolated from other species, including humans. If no objective data were available for a particular part of an assessment, we chose a welfare impact category based on informed judgement. Where there was doubt or a lack of objective knowledge about whether an animal would suffer severely, the animal was given the benefit of the doubt. Assessments considered the likely welfare impact in the majority of situations [42]. Where Sharp and Saunders had already made assessments of related methods, as used in Australia, e.g. shooting rabbits and birds, and trapping birds [74–76], we used these where appropriate as a basis for our assessments, adding or omitting literature to ensure assessments were made according to the British SOPs.

## Results

### Comparing welfare impact scores

Results of the welfare assessments are summarised here (and full details are given in S9–S19 Tables). Widely varying impact scores were attributed to the different interventions used for each species. Scores on the two axes (numbers 1–8 and letters A-H) are not intended for comparison with each other [42], but nevertheless useful comparisons could usually be made, even between lethal and non-lethal interventions. Applying the model also demonstrated the value of making formalised welfare assessments for identifying components of interventions that pose a relatively greater welfare threat, or which are currently under-researched and poorly understood. These aspects are highlighted for each species below.

### Rabbits

**Fig 1:**
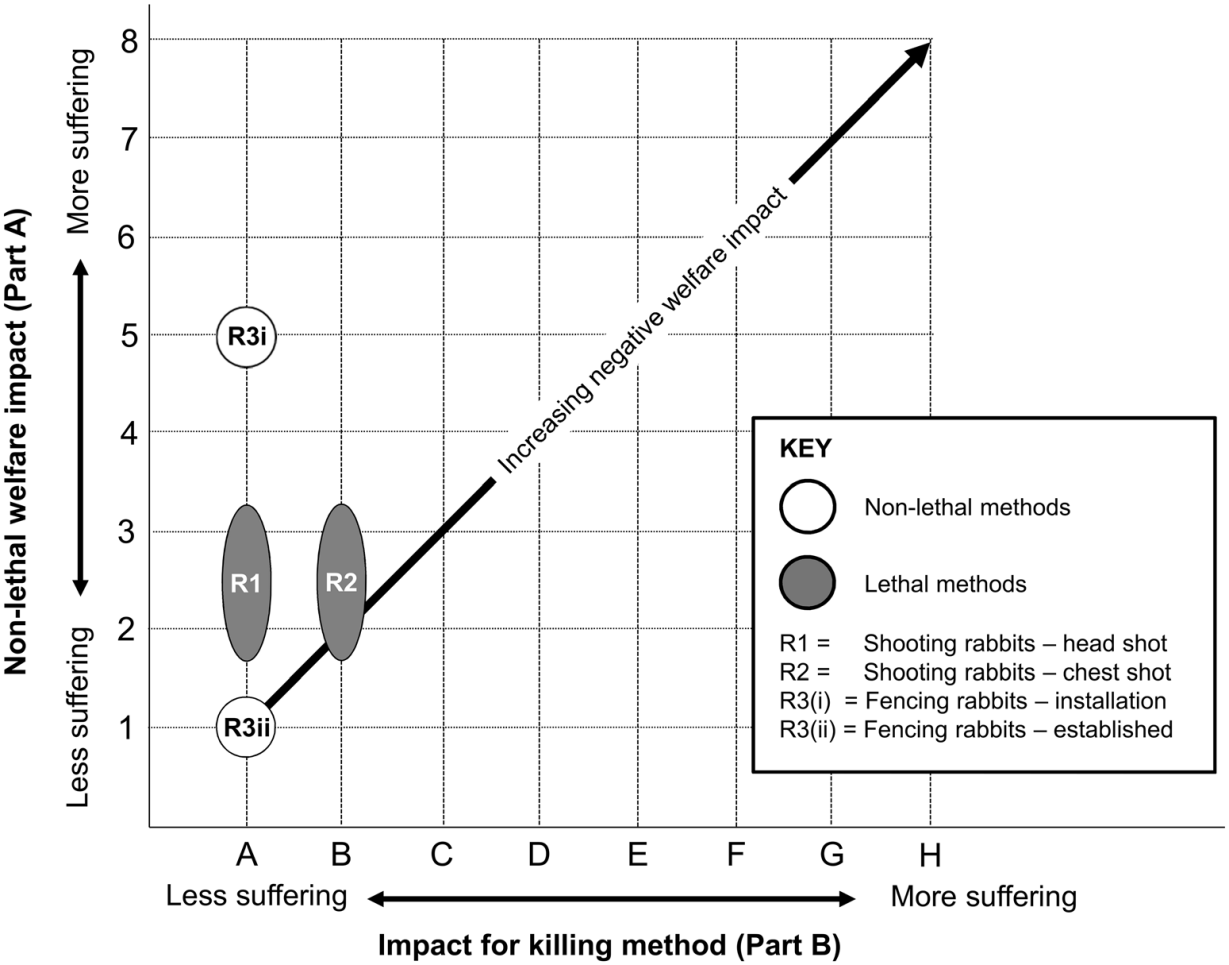
Welfare assessment grid for rabbit management interventions.

Rabbit management interventions were clearly ranked by the model (Fig 1). Shooting rabbits with a head shot scored a lower level of suffering (score A, Fig 1 R1; see S9 Table) for the killing method, under Part B of the model, than using a chest shot (score B, Fig 1 R2; see S10 Table). This difference originated from chest shots scoring ‘mild’ suffering over seconds (‘immediate/seconds’), and head shots no suffering (‘none’) [77] with death occurring immediately (also ‘immediate/seconds’) [78]. Chest shots were considered to produce only mild suffering in rabbits because of the relative scale of the damage likely to be inflicted, by a chest shot, to an animal the small size of a rabbit. Rabbits, shot elsewhere than in the head or chest, are likely to suffer more. The non-lethal welfare impact occurring before the rabbit was actually shot (under Part A of the model), of either a head or chest shot, was graded as ‘2–3’. This reflected the likelihood that multiple rabbits from the same social group [79], cited in [80], would be shot in a single shooting exercise and that most of these would therefore have experienced members of their social group being shot (‘mild’ impact)–together with the associated fear/panic from the noise and general disturbance—over a period of up to minutes (‘immediate/seconds’ to ‘minutes’), before they themselves were shot. Our assessments indicate that rabbit welfare could be significantly improved by targeting the head rather than the chest or any other body part. It is reassuring that the model aligns with this common-sense finding.

Because head shots scored no impact (score A) for the killing method (under Part B of the model), we were able to compare the other (non-lethal) impacts of head shots directly with those of wire-mesh fencing (under Part A of the model). Fence installation (in autumn, for the protection of imminently planted winter wheat) scored a greater impact (score 5, Fig 1 R3(i); see S11 Table) than shooting (score 2–3). This difference arose because, while both interventions were attributed a ‘mild’ impact, this was likely to last ‘days’ following fence installation (which would disturb nearby rabbit populations and interfere with rabbits’ usual access to foraging areas on other sides of the field) [81] and between no time (‘immediate/seconds’) and ‘minutes’ for a head shot (because a rabbit would suffer distress and behavioural restrictions when other rabbits are shot before it is). However, once a rabbit fence is properly installed (and rabbit ranging behaviour has adapted to the presence of the fence), it should, if well maintained, last for about 10 years [82], and without further welfare impact (score 1, Fig 1 R3(ii); see S12 Table).

### Moles

**Fig 2:**
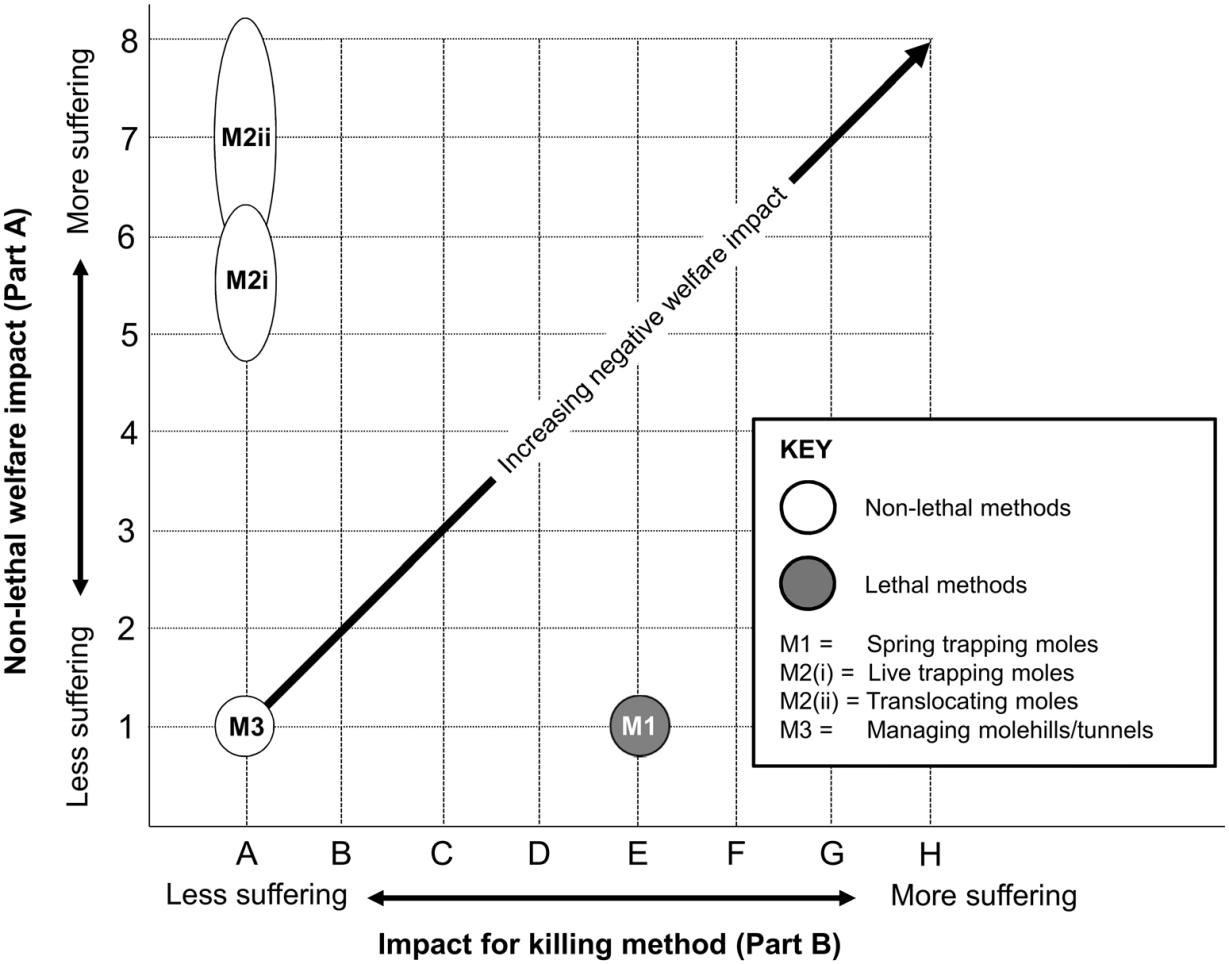
Welfare assessment grid for mole management interventions.

Of the three mole management options assessed, managing molehills and tunnels (Fig 2 M3) scored lowest with ‘no impact’ (score 1; see S13 Table). Spring trapping scored 1E (Fig 2 M1; see S14 Table), with no welfare impact (under Part A) before the trap was triggered, but the killing method scored ‘severe’ suffering, most likely as a result of acute haemorrhaging [83], potentially for ‘minutes’ (under Part B). Because mole traps are exempt from welfare approval, no data are available on how long it takes for spring trapped moles to reach irreversible unconsciousness and this is likely to remain the case unless the exemption is revoked. For the purposes of this assessment the SOP assumed time to irreversible unconsciousness (TIU) for ≥ 80% of moles trapped was ≤ 5 minutes, which is the requirement for non-exempt spring traps in England and Wales [84]. The suffering caused by spring trapping moles could be reduced if TIU was decreased [85].

Live trapping and translocation (Fig 2 M2(i) and M2(ii); see S15 and S16 Tables), which need to be considered cumulatively in a translocation exercise, scored non-lethal welfare impacts of 5–6 and 6–8 respectively. A range of potential impacts was attributed to both because there are very few data regarding their impact on moles. The live trapping component represented a ‘moderate’ to ‘severe’ impact for ‘hours’, largely because moles survive poorly in live traps (e.g. [83]) and the particular type of traps available and assessed here (tube traps) do not accommodate the provision of bedding [29]. The translocation component scored a ‘moderate’ to ‘extreme’ impact over several ‘days’, because of the risks involved in either encountering existing territory holders [86, 87] or dispersing above ground [88, 89], and the difficulties associated with setting up an independent territory of feeding tunnels [87]. The impacts of live trapping moles might be reduced by checking traps more frequently, or using different traps that allow the use of bedding [29], but the potential of both live trapping and translocation for managing moles needs more research to determine whether it can be done successfully and with acceptably low welfare impact levels [29]. These assessments highlight two issues with applying the model: first, how to compare total impact scores between some lethal and non-lethal interventions (as seen for spring trapping and for live-trapping followed by translocation, where only non-lethal scores can be compared, under Part A); and second, how to assess two methods used in sequence as part of a single management intervention (whether two non-lethal methods or a non-lethal followed by a lethal method).

### Crows

**Fig 3:**
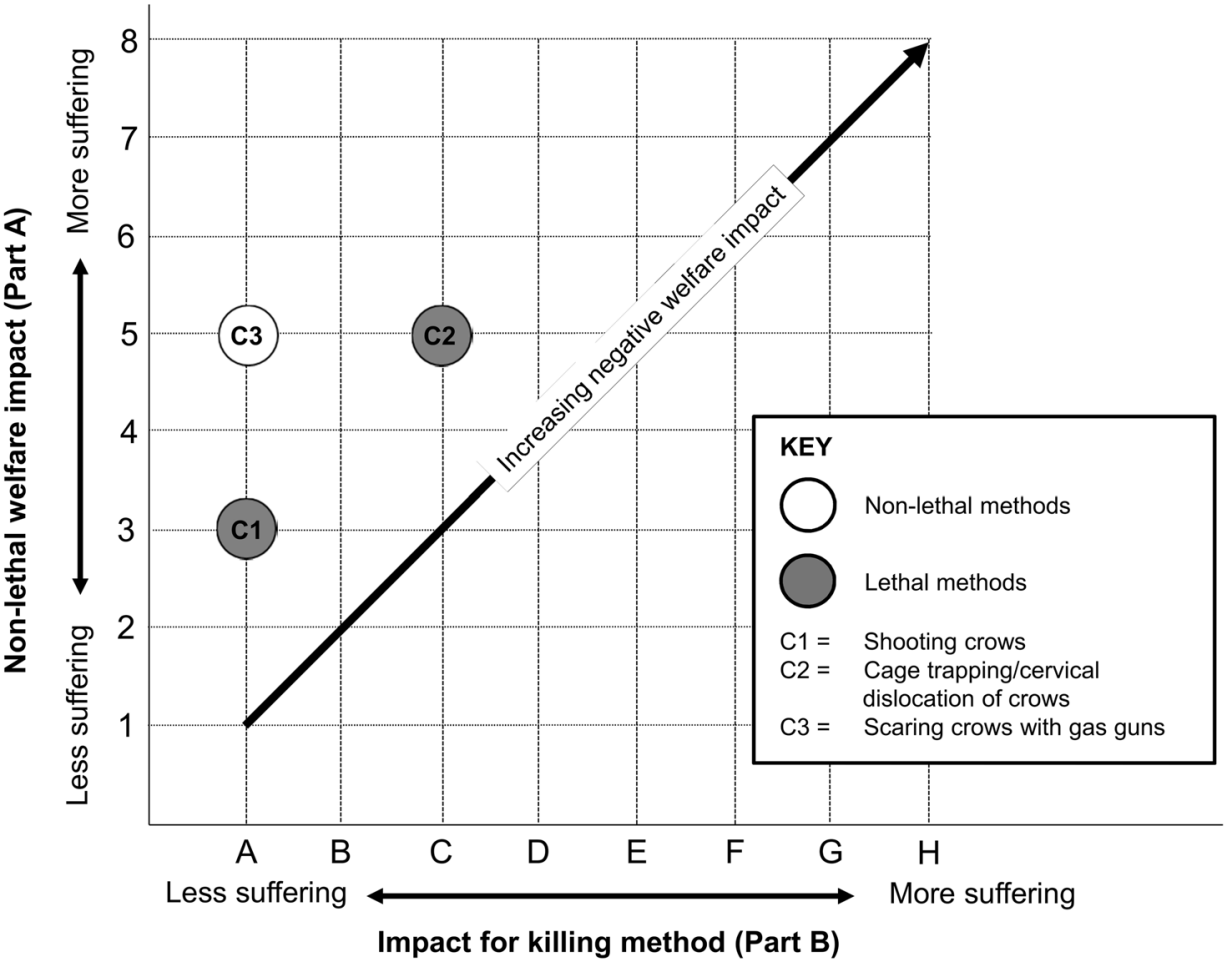
Welfare assessment grid for crow management interventions.

The relative welfare impacts of the three crow management interventions were clearly ranked by the model. Shooting scored the least impact (score 3A, Fig 3 C1; see S17 Table), with a ‘mild’ non-lethal welfare impact occurring, potentially for ‘minutes’, before the target crow itself was shot (Part A). Crows are often shot at in a group, and any particular individual is likely to be distressed by the shooting of nearby conspecifics before it is shot [90]. The actual shooting of the target crow scored ‘no impact’ (Part B) [91]. Non-lethal scaring using a propane gas gun had a greater impact (score 5, Fig 3 C3; S18 Table), involving a ‘mild’ non-lethal welfare impact, potentially lasting ‘days’, as a result of crows being disturbed by repeated noise from intermittent gunfire close to feeding or roosting areas [92]. Cage trapping with cervical dislocation had the greatest impact (score 5C, Fig 3 C2; see S19 Table), with trapping causing a ‘moderate’ non-lethal welfare impact over ‘hours’ (while the bird was in the trap) and the cervical dislocation causing ‘moderate’ but short-lived (‘immediate/seconds’) suffering [93]. Trapped birds can suffer distress, injury or panic during confinement in the trap (see [94] about unrestricted minimum cage size for crows), while birds being euthanized will be distressed by handling and the response of the decoy bird to the handler, and can experience hypoxia following cervical dislocation [95–97]. The assessment for cage trapping with cervical dislocation reveals that the impact of this intervention might be considerably reduced if traps were checked more frequently. However, reducing trap-checking frequency from once every 24, even to once every 2 hours, would not be detected by the model in its current form.

## Discussion

The Sharp and Saunders model, created in collaboration with experts in animal welfare, wildlife management and veterinary science [42], marks a turning-point in the assessment of welfare impacts in vertebrate wildlife management. The model provides a transparent, evidence-based method for ranking the relative welfare impacts of different management interventions involving lethal and/or non-lethal methods, and in doing so provides helpful information for decision-makers. It is designed for assessing impacts on individual target animals but assessments can also be made for each type of non-target animal likely to be affected. The scores attributed under Parts A (non-lethal welfare impact) and B (impact of killing method) are not intended to be comparable with each other but in our study the model allowed the ranking of most interventions, by their impacts, even including lethal / non-lethal comparisons (see also Sharp et al.’s assessments of lethal and non-lethal camel removal procedures [98]). The model distinguishes physical and affective (emotional) impacts, enabling better consideration of negative affective impacts [61] and it can be modified to address the assessment of a particular class of interventions, e.g. toxic agents [26], or spring traps. Importantly, the model allows identification of gaps in existing knowledge about welfare impacts (and the incorporation of new knowledge as this arises [61]), thus revealing where further research would be best directed. The model also highlights ways of improving SOPs to reduce welfare impacts, and provides a tool for informing debate on the acceptability of management interventions and for setting limits on acceptable and unacceptable levels of welfare impact [99].

### Assessments

Our assessments of management interventions used with rabbits, moles and crows indicated that applying the model produces helpful information for deciding which interventions to use, where interventions might be improved and where further research would best be directed. It must be borne in mind however that the assessments presented here are for interventions conducted according to a best practice SOP, and the relative welfare impacts of different interventions could be different if SOPs are not followed.

#### Rabbits

Our assessments for rabbits suggest that, while the installation of fencing in autumn may have a mild impact for a few days, established fencing has the least welfare impact (none), followed by head shots and then chest shots. However, in some situations, e.g. involving transitory rabbit damage, fencing may not be appropriate and shooting may be the best practical option. Shooters should target the rabbit’s head, because not only is the impact of a chest shot greater than that of a head shot, but it is also more variable [100], even when a chest shot is conducted well. It may be that shooters are unaware of this. Of course, the impact scores attributed to head and chest shots depend on how strictly the SOP is followed, e.g. in terms of weapon, ammunition and distance; rabbits shot not in accordance with the SOP are likely to suffer more, e.g. see Hampton et al [101], who found that the greater the shooting distance, the more likely a rabbit was to be wounded or missed. This raises questions about what proportion of the many thousands of rabbits shot in Britain each year are shot cleanly in the head, in the chest and elsewhere, and using the appropriate weapon, ammunition and range needed to achieve the welfare impact scores derived here.

#### Moles

Our assessments for moles indicate that managing molehills and tunnels according to the SOP is likely to have little or no welfare impact, while the two removal interventions, spring trapping, and live trapping followed by translocation, have large impacts on different axes. While impacts for these two interventions can be compared under Part A (spring trapping scored 1, whereas live trapping and translocation scored 5–6 and 6–8 respectively), they cannot be compared under Part B because non-lethal interventions are not be scored on this axis. We assumed that spring traps met the approval standard generally required under the UK Pests Act 1954 [18], that at least 80% of 12 animals should reach irreversible unconsciousness within 5 minutes [84, 85]. However mole traps are exempt from approval under the Small Ground Vermin Traps Order 1958 [102]. Probably because of this, there is wide variation in the mechanical performance of mole traps available in Britain, and therefore in their potential welfare impacts [85], and at least some might fail to meet approval standards if they were tested. Better information, on TIU for mole spring traps, is required before the impact of mole trapping can be properly assessed. Withdrawal of their exemption from regulation would help to ensure that mole spring traps at least meet the minimum standards assessed here. A recent report by the Food and Environment Research Agency recommended that spring traps for all species should be required to meet specified minimum welfare standards, and suggested that two additional, stricter welfare tiers should be introduced, giving three thresholds for TIU of 5 minutes, 3 minutes and 30 seconds [103]. Clearly, differences in TIU in the order of seconds, and very few minutes, are considered significant in welfare terms. However, because the model uses categorical time units (immediate/seconds, minutes, hours, days, weeks [42]), it may not always be sensitive to relatively large differences in the duration of suffering (and hence the welfare impact) under Parts A or B. For example, it would not distinguish traps taking 3, 5 or even 50 minutes to cause TIU, but where two interventions are attributed the same welfare impact score, their relative rankings might sometimes be determined simply by looking at the details underlying the assessments. Alternatively, the time categories of the model could be modified to usefully distinguish the impacts of, for example, different types of mole trap.

For mole live trapping we assessed the plastic tube trap, seemingly the only mole live trap available ‘off-the-shelf’ in Britain. While any live trap will prevent a trapped animal having normal interactions with conspecifics, the design of tube traps is particularly likely to compromise welfare. Being narrow, they restrict the movement of a trapped mole and do not allow the provision of bedding, or addition of a nest box, and the one-way swing doors potentially allow more than one animal to become trapped, which could lead to fighting [29]. Moles are poorly disposed to live trapping although it is not clear whether this is due to starvation, hypothermia or shock [87, 104]. And, because moles have a high metabolic rate, Natural England recommends that tube traps are checked more frequently than the usually recommended once every 24 hours [87]. Mellanby reported that moles may survive for 24 hours without food, but recommended that live traps are checked at least every 8 hours to prevent trapped moles from dying from hypothermia, starvation and from throwing themselves at the trap walls (although the latter would not be possible in the close-fitting tube trap) [104]. Of the eight moles trapped by Shaw et al, in Friesian and Talpa live traps (both types provisioned with food, and with nest boxes filled with hay), the two caught overnight (≤14 hours in the trap) were found dead in the morning, while the six caught during the day (≤10 hours in the trap) survived (Ros Shaw, University of Exeter, personal communication). Currently there is no legal requirement to check live traps (other than Larsen traps and snares which must be checked at least every 24 hours [94, 105]). Our assessment here related to mole tube traps being checked every 4 hours and it seems little can be done to improve their welfare impact, except checking them even more frequently (although the model would not distinguish between checking traps every 4 or every 2 hours). However, larger, bespoke wooden Friesian traps with a nest box attached, and allowing single captures only, might have a lower impact [88, 104]. Better information is needed on how to maximise mole welfare and survival in live traps, and better traps (or trapping procedures) could be designed for the mass market as a result.

Natural England warns that releasing a mole into an already occupied territory can lead to fighting, while releasing it into an area without an existing run (feeding tunnel) system may be an offence under The Animal Welfare Act 2006 [49, 87]. Natural England therefore recommends that live trapped moles are despatched humanely [49]. Better information is needed on how to identify whether an adequate, unoccupied run system exists at potential release sites, whether and in which conditions moles are capable of quickly establishing an adequate run system where necessary. Even if a mole could be live trapped well, there are currently insurmountable issues regarding mole translocation and it may not be possible to reduce welfare impacts adequately to justify this type of intervention with moles.

A more general point highlighted by the assessment for live trapping followed by translocation is that it is difficult to interpret together the sequential impacts of the two separate stages of an intervention (as we have attempted here). However, the advantage of assessing each component of an intervention separately, according to the model (as we have done), is that comparisons can be made between different secondary techniques following the same primary technique, thereby helping with decisions on what the best secondary technique might be. In the case of two or more non-lethal methods, used sequentially, an alternative approach would be to assess the component parts separately and then to score the combined process the same as the highest scoring component part (as done in the Australian assessments of camel removal and killing at an abattoir, a process consisting of several non-lethal stages before the killing method is applied [98]). While the overall score identified in this way may not be particularly informative by itself, the associated welfare assessment worksheets and matrix can be consulted to identify which of the component methods has the greatest welfare impact. In the case of mole live-trapping followed by translocation the overall score would be 6–8 (the score attributed to the translocation component on its own, which exceeded that for live-trapping).

#### Crows

Our assessments for crows suggest that shooting has least impact, followed by scaring, and then cage trapping with cervical dislocation. The impact of cage trapping could be reduced by increasing the frequency of trap-checking, but even if traps were checked every 2 hours, instead of every 24, the model would still rank it worst of the three interventions. In addition, Larsen trapping requires the use of a live decoy bird to lure target birds into the trap, and inevitably involves impacts on the decoy, whenever the trap is set, even when no target bird is caught. The model does not assess impacts on non-target animals, whether these are conspecifics (including decoys, dependent offspring, or would-be target animals that are impacted but not successfully targeted), or members of non-target species (such as predators, by-catch etc). However, a separate assessment can be made for each non-target animal likely to be affected [42].

Territorial animals (such as crows) removed from an area, e.g. by shooting, or cage trapping with cervical dislocation, are likely to be replaced by incoming conspecifics [106], which might then also be removed. However, crows that were previously scared away might return to the site, and be scared again, or be replaced by new individuals that would in turn be scared away. Crows can only be shot or cage trapped under Natural England General Licence WML-GL04 and only “where the authorised person is satisfied that appropriate legal methods of resolving the problem, such as scaring and proofing, are either ineffective or impracticable” [107]. If birds have been subjected to scaring, before being shot or cage trapped, any welfare impacts might be compounded, potentially over an extended time-scale. This brings us back to the issue of sequential impacts in general and where the line should be drawn regarding what is considered a single impact, and what is considered two, to be scored separately? This is especially relevant for ongoing or repeated non-lethal interventions, e.g. scaring / hazing or mustering. Here we treated a 2-month period of crow scaring as a single intervention (and for rabbits, assessed the impact of fencing over 2 months), and while the model may be used in this way to assess the impact of individual bouts of an intervention, this would not take account of potential cumulative effects.

### The model

We have drawn attention to some issues raised by the model and we explore here the challenges in trying to address them.

#### Comparability of assessments

An ideal welfare assessment model would allow direct comparison of the total impact of any management interventions, whether lethal or non-lethal. However, for example, while we were able to compare the non-lethal impacts of spring trapping moles with those of live-trapping followed by translocation, we were not able to rank the two interventions on the basis of their total impacts (including lethal impact of spring trapping) because scales on the two axes are not (and are not intended to be) comparable. The scores for lethal and non-lethal impacts are expressed separately (and represented on separate axes), under the Sharp and Saunders model, because it uses two different systems to assess lethal and non-lethal impacts. A model using a single system to assess both lethal and non-lethal impacts (the results of which could be shown on a single axis), would be ideal, but difficulties in designing such a model include producing a single set of impact scales relating to both lethal and non-lethal impacts, and combining these scores in the case of lethal interventions. In reality such a model might be impossible to achieve but the Sharp and Saunders model goes part way towards this.

The total welfare impacts of interventions scoring on two different scales would be comparable if the scores on the two axes of the model (1–8 and A-H) were comparable, e.g. 3 = C, and interval in nature, such that each impact increment on either axis was of equal value (e.g. 1xF = 2xC). However there is a difficulty with this because it is not possible to create with confidence impact scores that are either interval, or comparable between axes. The scores on both axes of the model (1–8 for Part A and A-H for Part B) are the product of impact (or severity) categories on an ordinal scale (no impact, mild, moderate, severe, extreme), and time categories on an irregular, semi-logarithmic scale (seconds, minutes, hours, days, weeks). (See scoring matrices in S6 and S8 Tables). And so, while the impact categories are ordinal (no impact < mild < moderate < severe < extreme), the difference between pairs of adjacent categories do not necessarily correspond to an equivalent change in welfare status [61], e.g. the increase in severity between moderate and severe, may not be the same as that between severe and extreme. The same is true for the time categories, in the scoring matrices, which increase by irregular quantities (60 seconds per minute, 60 minutes per hour, 24 hours per day, 7 days per week). It is most likely not possible to create interval scales of welfare impact severity and duration, unless suffering is known to increase consistently and the pattern of increase does not depend on the type of suffering involved [53].

Once this limitation of the model is understood however its real benefits can be appreciated. The model is intended to provide an overall view of relative welfare impacts of a range of different management interventions. The numerical and alphabetical scores are discrete ordinal scores, which when placed on a grid can facilitate the visual comparison of welfare impact across a range of management methods [61].

#### Breadth of impact scores

Each of the impact scores produced using the model can be arrived at in a number of different ways. For example, a score of ‘E’ under Part B of the model could represent anything from an extreme impact lasting seconds, right through to a mild impact lasting days. However, because of the irregular nature of the impact and time scales underlying these scores, not all interventions attributed the same score can necessarily be considered equivalent. Also, because of the breadth of each of the time categories, the model may not always be sufficiently sensitive to detect potentially important differences in impact scores, such as those dependent on, for example, trap-checking times or TIU. Beausoleil and Mellor [61] make the related point that the model outputs may suggest a precision that is not currently possible. However, the model was developed to assess and compare the wide range of methods currently used in Australia and as a result the scales are quite coarse in order to accommodate methods taking seconds and those taking weeks; the model also worked well in our study. In cases where a group of similar methods need to be assessed (e.g. different mole traps, as mentioned above, or different types of poisons) the duration timescales could be adapted to make the intervals finer.

#### Over and underestimation of impacts

A model is necessarily imperfect, and the Sharp and Saunders model might, in some cases, overestimate or underestimate the welfare impact of an intervention. This could happen in several ways: 1) if there is doubt about the impact on an animal, the rules of the model require that the animal is given the benefit of the doubt (potentially overestimating the impact); 2) the model is supposed to assign the greatest welfare impact scores occurring at any point in the management process, but also to assume that the duration of impact or suffering is the total duration of impact or suffering regardless of how much of this was at the maximal level (also potentially overestimating); 3) application of the model is based on a SOP for, and on various assumptions about, the intervention, stated at the outset (potentially overestimating or underestimating welfare impacts if the SOP is not followed); and 4) the model assesses what happens to the majority of animals, e.g. 51–100% of the animals affected (in extreme examples it might be possible for 51% of animals to suffer enormously, and 49% only a little, or vice-versa, potentially leading to large over or underestimation of the impact). Where the welfare impact of an intervention is overestimated, this might be considered to protect the welfare of an animal [42]. However, because the model is used for measuring relative welfare impacts of different interventions, overestimating the impact of one intervention might potentially improve the relative ranking of another, which actually has a greater welfare impact. If this worse (but higher ranking) intervention was selected for use as a result, this might result in a greater level of animal suffering as a result of the intervention. Of course, it may not be possible to detect an over or underestimation, but attention should be drawn to either, where it is suspected to have occurred, so that anyone interpreting impact scores can examine the details of the assessment.

#### Lack of scientific data

Our assessments illustrate that there are often large gaps in scientific understanding of the animal welfare impacts of management interventions. Often there may be no information about what happens to the majority of animals and when objective data are lacking, assessments—rather than being evidence-based—will be based on guidance from a range of experts. A benefit of the model is that it can help to identify precisely where future research should be targeted.

### Wider perspectives

It is increasingly important that humaneness is considered when deciding how (and even whether) human-wildlife conflict should be tackled [2, 43]. Managers need to think and rethink about management more often, as pest situations differ or change, management options evolve and improve, and knowledge about impacts increases. However, managers may have preconceptions about lethal and non-lethal interventions, or may turn automatically to familiar, favoured methods without much thought. Even if managers do try to take the humaneness of interventions into account, their opinions on the impacts of different methods on animal welfare may vary widely [4, 50]. Also, the real welfare impact of a method can sometimes be counter-intuitive to our perceptions of impact; our assessments revealed that the relative welfare impacts of different interventions are not always easy to predict, thus confirming the need for objective formal assessments. For example, live-trapping and translocating moles may be associated with a very high welfare impact, although to many, this intervention may intuitively seem ‘the kindest option’ for managing moles. Shooting crows is likely to cause very little welfare impact prior to death, whereas scaring crows with gas guns may result in a short period of food restriction, noise and fear/distress before they leave the area. And while shooting rabbits with a head shot is unlikely to have much impact on their welfare before death, installing rabbit fencing may result in a short period of food and behavioural restrictions and fear/distress for rabbits (although—once established—fencing may have no further welfare impact). Littin et al. propose that wildlife managers should use the most humane methods that achieve the aims in any given situation, which means that any decision regarding choice of method needs to incorporate other considerations, e.g. ethics and conservation (as in the latter examples), as well as whether the method is effective and easy to use, affordable, safe for human users and other people, specific to the target species or individuals, and safe for the environment [27]. While it might seem desirable to develop a holistic model that could help to achieve the right balance of all these factors, this would be complex and would require judgements to be made about the relative importance of potentially competing priorities. As it stands the model is a useful tool for simplifying and clarifying the details of welfare impacts, so that these can be taken into account consistently in any decisions about wildlife management.

Assessing welfare impacts in wildlife management is much more difficult than doing the same in livestock farming or laboratory animal experimentation, where managers have more (in some cases, complete) control over the details and duration of any intervention they make, and with greater knowledge of how many, and which, individuals are affected. In wildlife management, impacts can be influenced by a host of environmental factors, including the weather, and which and how many target and non-target animals happen to enter the fray. The total animal welfare impact of a particular wildlife management operation will depend on the severity of impact on an individual and the number of individuals involved (for both target and non-target animals) [31, 65, 108]; this may be seen as an ethical or philosophical issue that can be considered after the severity of impact on an individual animal is known (e.g. see [21]).

Also, there are wider issues about the balance between the number of animals and the level of suffering involved, and uncertainties about how a target population responds to an intervention. The balance of numbers and suffering is especially relevant for most non-lethal interventions, which often maintain an unknown number of the same individuals in their habitat, subjecting them to repeated or even continuous welfare impacts which are difficult to compare with those resulting from a removal intervention (culling or translocation), conducted in a different pattern, potentially on a known number of animals, each affected once. In our case of a cohort of rabbits suffering the mild impacts of a new fence for a few days, compared to ten cohorts (each potentially consisting of multiple litters [109]) of rabbits managed by shooting (as well as individuals born to fill vacancies created, within a cohort, by density-dependent responses [110]), it is relatively easy to judge that over a 10 year period the total welfare impact of a fencing operation (including installation) will be less than that of an annual shooting operation. In other comparisons it might be far less obvious which option involves less total suffering. Also there may be uncertainties about how a target population will respond to an intervention, for example, where an animal is removed (by killing or translocation) it may be replaced by another, and in time others [106], which may then be subject to the same or a different intervention. Conversely, an animal exposed to certain types of non-lethal control may be subjected repeatedly to the same or different interventions. It may be useful therefore to think in terms of both the ‘lifetime welfare impact’ of multiple interventions on a particular individual, and perhaps the ‘community welfare impact’ of an intervention on all animals affected by it. Despite the difficulties involved, it is important that welfare scientists continue to develop tools for assessing the humaneness of wildlife management interventions and for reducing these impacts where possible.

There are some parallels between Sharp and Saunders’ welfare assessment model and the quality-adjusted life years (QALYs) system used to provide a common currency for measuring the extent of health benefits resulting from human healthcare interventions and for making choices based on this. The QALYs system incorporates the quality and quantity of life that a patient can expect following a particular medical treatment (represented as a positive score) [111]. Similarly, but in reverse, Sharp and Saunders’ model provides a common currency for measuring the extent of welfare impacts resulting from wildlife management interventions. Broadly speaking, the model incorporates the welfare impact and duration of suffering associated with an intervention (represented as a negative score). As well as informing decision making about the humaneness of different interventions, the model can be used for identifying ways of reducing the welfare impact of an intervention and for directing future research as discussed above. The model could also be used to improve the way that interventions are practised, e.g., by comparing the welfare impacts of an intervention conducted according to a SOP (based, for example, on an organisation’s Code of Practice or Best Practice Guidelines) and the impacts of the same intervention conducted as it is in practice (perhaps by that same organisation’s members). This could help to identify where pressure might most efficiently be applied to improve wildlife management practise, in order to benefit animal welfare.

For the purposes of this study welfare assessments were performed to demonstrate the application of the model and various related issues. Our plan for taking this work forward is to lead assessments using a panel of experts in an effort to reach consensus (a Delphi approach) on the relative welfare impacts of a much wider range of management interventions on a larger number of species. The participation of, and agreement among, diverse stakeholders should reduce any effects of subjective judgement and increase acceptance of the resulting impact scores.

## Supporting Information

S1 SOP Standard Operating Procedure for shooting rabbits. (PDF)

S2 SOP Standard Operating Procedure for fencing rabbits from crops. (PDF)

S3 SOP Standard Operating Procedure for spring trapping moles. (PDF)

S4 SOP Standard Operating Procedure for live trapping and translocation of moles. (PDF)

S5 SOP Standard Operating Procedure for managing molehills and tunnels on lawns. (PDF)

S6 SOP Standard Operating Procedure for shooting crows. (PDF)

S7 SOP Standard Operating Procedure for cage trapping and cervical dislocation of crows. (PDF)

S8 SOP Standard Operating Procedure for scaring crows using gas guns. (PDF)

S1 Table Impact scale for part A of the model (assessment of non-lethal welfare impact) Domain 1: Water deprivation, food deprivation, malnutrition. From Sharp and Saunders (2011).
(PDF)

S2 Table Impact scale for part A of the model (assessment of non-lethal welfare impact) Domain 2: Environmental challenge. From Sharp and Saunders (2011).
(PDF)

S3 Table Impact scale for part A of the model (assessment of non-lethal welfare impact) Domain 3: Injury, disease, functional impairment. From Sharp and Saunders (2011).
(PDF)

S4 Table Impact scale for part A of the model (assessment of non-lethal welfare impact) Domain 4: Behavioural, interactive restriction. From Sharp and Saunders (2011).
(PDF)

S5 Table Impact scale for part A of the model (assessment of non-lethal welfare impact) Domain 5: Anxiety, fear, pain, distress, thirst, hunger etc. From Sharp and Saunders (2011).
(PDF)

S6 Table Scoring matrix for part A of the model: assessment of non-lethal welfare impact. From Sharp and Saunders (2011).
(PDF)

S7 Table Impact scale for part B of the model (assessment of killing method). From Sharp and Saunders (2011).
(PDF)

S8 Table Scoring matrix for part B of the model: assessment of mode of death. From Sharp and Saunders (2011).
(PDF)

S9 Table Welfare assessment for shooting rabbits (head shot). (PDF)

S10 Table Welfare assessment for shooting rabbits (chest shot). (PDF)

S11 Table Welfare assessment for fencing against rabbits (fence installation). (PDF)

S12 Table Welfare assessment for fencing against rabbits (fence established). (PDF)

S13 Table Welfare assessment for managing molehills on lawns. (PDF)

S14 Table Welfare assessment for spring trapping moles. (PDF)

S15 Table Welfare assessment for live-trapping moles. (PDF)

S16 Table Welfare assessment for translocating moles. (PDF)

S17 Table Welfare assessment of shooting crows. (PDF)

S18 Table Welfare assessment for scaring crows. (PDF)

S19 Table Welfare assessment for cage-trapping and cervical dislocation of crows. (PDF)
