# Supplementary material for: Assessing Animal Welfare Impacts in the Management of European Rabbits (Oryctolagus cuniculus), European Moles (Talpa europaea) and Carrion Crows (Corvus corone)
Source: PLoS One. 2016 Jan 4;11(1):e0146298. doi: 10.1371/journal.pone.0146298 (PMC4699632; doi:10.1371/journal.pone.0146298)

Shooting rabbits

Background

European rabbits (*Oryctolagus cuniculus*) are often shot in the UK either as pests of agricultural, horticultural, forestry or amenity interests, or as food. Other rabbit control methods include gassing with phosphine, spring trapping, live trapping (cage or drop-box), snaring, ferreting, hunting with dogs, stalking or flushing with dogs and shooting, long-netting, warren and harbour destruction, exclusion fencing (wire mesh or electric), habitat management, tree-guards and shelters, and repellents.

Shooting is usually done at night with the aid of a spotlight (lamping), but can also be conducted during the day. Although shooting may be useful when rabbit numbers are already low, it is labour intensive and may not be effective as a general rabbit control method. Shooting can be a humane way of destroying rabbits when carried out by experienced, skilled and responsible shooters, when the animal can be seen clearly and is within range, and when the correct firearm, ammunition and shot placement are used. Rabbits may be shot using a rifle, a shotgun or an air rifle. However, this Standard Operating Procedure (SOP) covers shooting rabbits with either a rifle or a shotgun, because air rifles are suitable only at very short range and therefore less practical for shooting rabbits. This SOP is a guide only; it does not replace or override the legislation and should only be used subject to the applicable legal requirements.

Application

• Owners or occupiers of land may be legally obliged to carry out rabbit control at any time of the year under the Pests Act 1954, the Agricultural Act 1947 and the Agriculture (Scotland) Act 1948. These are likely to apply where horticultural or agricultural crops require protection.
• The Ground Game Act 1880 and the Wildlife and Countryside Act 1981 control who may shoot rabbits during the day and during the night. There is no close season for rabbits or prohibited time of taking with the exception of the provisions of the Ground Game Acts 1880 and 1906, relating to the taking of rabbits on moorland and on unenclosed land.
• Shooting rabbits is a popular management approach, but tends to target larger males and has limited effect on breeding potential the following spring, unless considerable time and effort are

spent. While ineffective in significantly reducing rabbit populations, or even maintaining them at low levels, shooting may have limited use in controlling light rabbit infestations.

• Shooting should only be used strategically, as part of a coordinated programme designed to achieve sustained effective control. It should only be used as an adjunct to more effective methods (e.g. fumigation) or to remove problem animals that cannot be disposed of by other means. Ferrets or dogs can be used to drive rabbits to guns. Habitat management should be incorporated to reduce harbourage.

• Shooting for the control of rabbits is most effective between November (possibly earlier on heavily grazed autumn cereals) and March and when conducted at night using a spotlight. Single operations are not particularly effective (reducing rabbit numbers by only about 30%).

• Shooting is most suited to open ground with a minimum of cover. It is not suitable in wooded areas or in the vicinity of human habitation. Shooting should be concentrated in rabbit feeding areas, normally indicated by shortly cropped grass with rabbit scratchings and droppings.

• Only a limited number of rabbits can be shot, at one time, in one place, before the remainder takes flight. In order to kill substantial numbers, regular visits are required to several places in turn. However, rabbits become wary after repeated shooting and do not show themselves, giving the erroneous impression that they have all been killed or driven away.

• Ineffective shooting may produce rabbits that are ‘shy’ of both guns and spotlights; in such cases shooting operations should be suspended for several months.

• Hunting rabbits with dogs is exempt hunting under the Hunting Act 2004. However in the context of shooting, dogs may also be used to flush rabbits from vegetation or from underground for subsequent shooting. These exemptions from the Act apply only on the shooter’s own land or on other land with the permission of the occupier or owner. Dogs may also be used in the retrieval of quarry.

• Shooting of rabbits should only be performed by skilled operators who have the necessary experience with firearms and who hold the appropriate licences and accreditation. Storage and transportation of firearms and ammunition must comply with relevant legislation requirements.

• The use of firearms is regulated under The Firearms Act 1968. In general rifles need to be licensed on a firearms certificate and shotguns on a shotgun certificate. Shooters are advised to carry their certificate with them when shooting.

• The Game and Wildlife Conservation Trust and other organisations have together produced a Code of Good Shooting Practice, while BASC have produced Codes of Practice on good shooting practice, shotgun use, lamping and respect for quarry.

STANDARD OPERATING PROCEDURE

Animal Welfare Considerations

Impact on target animals

• Shooting is not a single, standard technique; there are many factors that can vary and influence the humaneness of the method. These include the type of weapon (rifle or shotgun), calibre, choke, size and number of shot and load, range, ability of the shooter, movement and direction of the rabbit, exposure time, terrain and weather.
• Humaneness of shooting as a control technique depends almost entirely on the skill and judgement of the shooter. If properly carried out (appropriate firearms used, shots on target and in range, causing immediate unconsciousness and death), it is one of the most humane methods of destroying rabbits. On the other hand, if inexpertly carried out, shooting can result in wounding which may cause considerable pain and suffering.
• Shooting must be conducted in a manner which aims to cause immediate insensibility and painless death. The appropriate firearms and ammunition must always be used.
• Shooters should not shoot at an animal unless it is clearly visible and they are confident of killing it with a single shot. They must be competent at estimating range and shoot within the limitations of their equipment to kill cleanly and consistently. Shooters should practice target accuracy on clays.
• Head (brain) or chest (heart-lung) shots must be used. Shooting at other parts of the body is unacceptable.
• The shooter must be certain that each animal is dead before another is targeted.
• Wounded rabbits must be located and dispatched as quickly and humanely as possible with a second shot, preferably directed to the head. If left, wounded animals can suffer from the disabling effects of the injury, from sickness due to infection of the wound, and from pain created by the wound.
• If lactating rabbits are shot, reasonable efforts should be made to find dependent kittens and kill them quickly and humanely.

Impact on non-target animals

• Shooting is relatively target specific and does not usually impact on other species. However, there is a risk of injuring or killing non-target animals, including livestock, if shots are taken at movement, colour, shape, sound or, when spotlighting, eye reflection (‘eye shine’). Only shoot at the target animal once it has been positively identified. Also, never shoot over the top of hills or ridges as other animals may be out of sight beyond the hill in the danger zone.

STANDARD OPERATING PROCEDURE

• Shooting should be used with caution around lambing paddocks as it may disturb the lambing flock and cause mis-mothering. Also, avoid paddocks containing horses or deer. They are easily frightened by spotlights and gunshots and may injure themselves by running into fences and other obstacles. Sound moderators should be considered for both rim-fire and centre-fire rifles particularly in areas close to human habitation or livestock.

Health and Safety Considerations

• It is an offence (except in certain circumstances) to possess, purchase or acquire a firearm or ammunition without an appropriate firearms certificate or temporary police permit.

• Firearms are potentially hazardous. Users should observe all relevant guidelines on ownership, possession and use. Users should follow BASC's Best Practice guidelines on shooting and gun use, e.g. when carrying, transporting, holding, passing, using, maintaining and storing a gun. Always handle a gun as if loaded, carry it empty, in a case or open and over the crook of the arm, keep the safety catch on until the shot is about to be taken and only touch the trigger when firing a shot. It is an offence to be in a public place with a loaded firearm under The Firearms Act 1968.

• Maintain guns in good condition.

• Ensure ammunition is compatible with the gun and of an appropriate type and size for shooting rabbits. Do not mix cartridges of different bores.

• When not in use, firearms must be stored securely in a purpose-built firearms cabinet that meets UK legal requirements, to prevent access by unauthorised persons. Ammunition must be stored in a locked container in cool, dry conditions, separate from firearms.

• It is advisable to have adequate legal liability (third party) insurance when shooting.

• Take measures to ensure the safety of people not involved in shooting, including those using roads, footpaths, bridleways etc. Do not shoot across public rights of way. Take special care to warn riders using rights of way in earshot as horses may be easily frightened by loud noises, potentially threatening the safety of riders, horses, road users and others nearby.

• Never shoot at or near overhead power lines or insulators.

• Always carry a mobile phone, and where possible a six-figure grid reference of the area for emergency purposes.

• When a rabbit is being shot, any other people should stand well behind the shooter. The line of fire must be chosen to prevent accidents or injury from stray bullets or ricochets.

STANDARD OPERATING PROCEDURE

• Shooting from a vehicle is potentially dangerous – never shoot when the vehicle is moving. An agreed safety procedure between the shooter and others in the vehicle must be in place to ensure that people do not enter the field of fire or disturb the taking of a shot.

• Only take safe shots, when there is no risk of injury to other people and when the target can be identified as a rabbit with certainty.

• Ear defenders should be worn by the shooter and others in the immediate vicinity of the shooter. Repeated exposure to firearm noise can cause irreversible hearing damage.

• Safety glasses or goggles are recommended to protect eyes from gases, metal fragments and other particles.

• Warm, comfortable clothing and stout footwear to ensure a good grip and traction when taking up a firing position is recommended, especially when shooting at night.

Equipment Required

Firearms and ammunition

• For distances up to around 80 metres, small bore rifles fitted with a telescopic sight are recommended e.g. .22 rimfire. Centre-fire rifles e.g. .22 Hornet, .223 Rem, could be used in areas where long shots are required. Hollow-point or soft-nosed ammunition should be used.

• For a moving target at ranges less than 20 metres, a 12-gauge shotgun with shot sizes between No. 4 and No. 6 may be used. However, shotguns are not recommended for shooting rabbits because their noise level will drive nearby rabbits to cover.

• If intending to use steel shot ensure that it is safe and effective to do so in your gun. Steel pellets should only be discharged in modern guns that are capable of withstanding the extra stresses produced.

• Ensure that the choke configuration of a shotgun delivers a dense pattern on the target within the specified distances.

• The accuracy and precision of firearms should be tested against inanimate targets prior to the commencement of any shooting operation. Pattern your chosen gun/cartridge/choke combination before shooting to check your accuracy and that the pattern is adequate for the intended target animal.

Other equipment:

• If shooting at night, a handheld spotlight (at least 100 watt), or a helmet or headband mounted 12 volt (35 watt) spotlight.

• First Aid kit.

- Lockable firearm box.
- Lockable ammunition box.
- Personal protective equipment (hearing and eye protection).

Procedures

Assessment of site and estimation of rabbit numbers

- To maximise effect on rabbit populations, conduct a careful on-site risk assessment to confirm the need for shooting and assess the suitability of the area before shooting begins. Evidence of active warrens may include fresh rabbit droppings, tracks, mounds, or diggings.
- The density of rabbits on the site should be estimated using spotlight counts and warren monitoring. The location and numbers of rabbits on neighbouring properties should also be approximated.
- During daylight hours, shooters should identify suitable shooting points, firing directions and back-stops, and familiarise themselves with the terrain they are to cover, taking note of potential hazards and any landmarks that may help with navigation.
- Contact Natural England's Wildlife Management Advisors for more information and advice on site assessment and monitoring of rabbit numbers.

Shooting at night

- Where appropriate, notify landowners, tenants and neighbours about the planned shoot. Ensure that shooting does not obstruct, or cause danger or alarm to users of, the public highway, including roads, bridleways, footpaths and other rights of way. In particular, care should be taken when siting guns near roads. Pay particular attention when shooting close to field boundaries. On shoot days, erect information signs on nearby footpaths or bridleways.
- Most shooting of rabbits is done at night with the aid of a spotlight to locate them while they are feeding, or are away from cover. This method relies on the ability of the shooter to approach the animal until it is in shooting range.
- When shooting at night it is essential that the appropriate firearms and ammunition are used. Correct range judging is also essential to ensure effective shooting.
- Rabbits must NOT be shot from a moving vehicle or other moving platform as this can significantly detract from the shooters' accuracy. Ensure you are in a firm, safe and stable position before taking a shot.
- Shooting over the top of hills or ridges produces unacceptable risk. Be aware that the spotlight only illuminates a small portion of the danger zone and only a fraction of the projectile's range.

• When illuminated by the spotlight, rabbits have a pink/red eye shine. However, shooters should never shoot at a pair of eyes (binoculars may appear as 'a pair of eyes', particularly when reflecting red filtered light), but must instead identify the body of the animal.

Shooting in the day

• Where appropriate, notify landowners, tenants and neighbours about the planned shoot. Ensure that shooting does not obstruct, or cause danger or alarm to users of, the public highway, including roads, bridleways, footpaths and other rights of way. In particular, care should be taken when siting guns near roads. On shoot days, erect information signs on nearby footpaths or bridleways.
• Rabbit activity is mainly nocturnal or crepuscular, so shooting during the day is less effective than shooting at night with a spotlight.

Target animal and point of aim

• The objective is to fire at the closest range practicable in order to reduce the risk of non-lethal wounding. Accuracy is important to achieve a humane death. One shot should ensure instantaneous loss of consciousness and rapid death without resumption of consciousness.
• A rabbit should only be shot at when:
  - It can be clearly seen and recognised;
  - It is within the effective range of the firearm and ammunition being used; and
  - A humane kill is probable. If in doubt, do NOT shoot.

• The shooter must aim either at the head, to destroy the major centres at the back of the brain near the spinal cord or, at the chest, to destroy the heart, lungs and great blood vessels. This can be achieved by one of the following methods (and see diagrams in Appendix):

<u>Head Shot</u> (this is the preferred point of aim)

Frontal position (front view)

The firearm is aimed at the centre of the head between the eyes.

Temporal (side view)

The firearm is aimed at a point between the eye and the base of the ear directed towards the opposite eye.

STANDARD OPERATING PROCEDURE

<u>Chest Shot</u>

Side view

The firearm is aimed horizontally slightly to the rear of the shoulder.

• When using a rifle, the target animal must be stationary and within a range that permits accurate placement of the shot. Shots to the head are preferred over chest shots.

• When using a shotgun, the target animal may be stationary or mobile, but must be no more than 20 metres from the shooter and no less than two metres from a burrow.

The pattern of shot should be centred on the head or chest. It is essential that the distance to the target animal is accurately judged. To achieve adequate penetration of shot, the animal must be in range. It is recommended that shooters practice estimating distances before a shooting operation.

• The target animal should be retrieved and checked to ensure it is dead before moving on to the next animal. Shooting should not occur where quarry cannot be retrieved. Death of shot animals should always be confirmed by observing the following:

  - Absence of rhythmic, respiratory movements;
  - Absence of eye protection reflex (corneal reflex) or 'blink';
  - A fixed, glazed expression in the eyes; and
  - Loss of colour in mucous membranes (become mottled and pale without refill after pressure is applied).

• If death cannot be verified, wounded quarry should be despatched immediately to minimise suffering, either with a second shot or a sharp blow to the base of the skull.

Assessing effectiveness

• Rabbit damage levels should be assessed regularly to determine whether further control is necessary.

References

This SOP was adapted from RAB009 ground shooting of rabbit warrens, prepared by Trudy Sharp (2012).

BASC (2008) Code of Good Shooting Practice. http://www.basc.org.uk/en/codes-of-practice/code-of-good-shooting-practice.cfm.

BASC (2010) Lamping (Night Shooting); A Code of Practice. http://www.basc.org.uk/en/codes-of-practice/lamping.cfm.

BASC (2010) Respect for Quarry; A Code of Practice. http://www.basc.org.uk/en/codes-of-practice/respect-for-quarry.cfm.

BASC (2010) Shotgun Code of Practice. http://www.basc.org.uk/en/codes-of-practice/shotgun-code-of-practice.cfm.

Defra (2005) Report of the Independent Working Group on Snares http://archive.defra.gov.uk/wildlife-pets/wildlife/management/documents/snares-iwgs-report.pdf.

Forestry Authority (1998) The Prevention of Rabbit Damage to Trees in Woodland; FCPN002 http://www.forestry.gov.uk/PDF/fcpn2.pdf/$FILE/fcpn2.pdf.

Natural England (2011) Rabbits: management options for preventing damage; TIN003 http://publications.naturalengland.org.uk/publication/19005?category=41004.

Rees, W.A., Ross, J., Cowan, D.P., Tittensor, A.M. and Trout, R. (1985) Humane Control of rabbits. In *Humane Killing of Animals*. pp 96-104. 4th Edition. Universities Federation for Animal Welfare, Potters Bar, England, UK.

Sharp T (2012) RAB009: Ground shooting of rabbits; PESTSMART Standard Operating Procedure. Invasive Animals Co-operative Research Centre, Australian Government. http://www.pestsmart.org.au/wp-content/uploads/2013/08/RAB009_ground-shooting.pdf

Appendix: Recommended shot placements for rabbits (from Sharp 2012, with kind permission of New South Wales Department of Primary Industries).

Recommended shot placements - Rabbit

Diagram 1

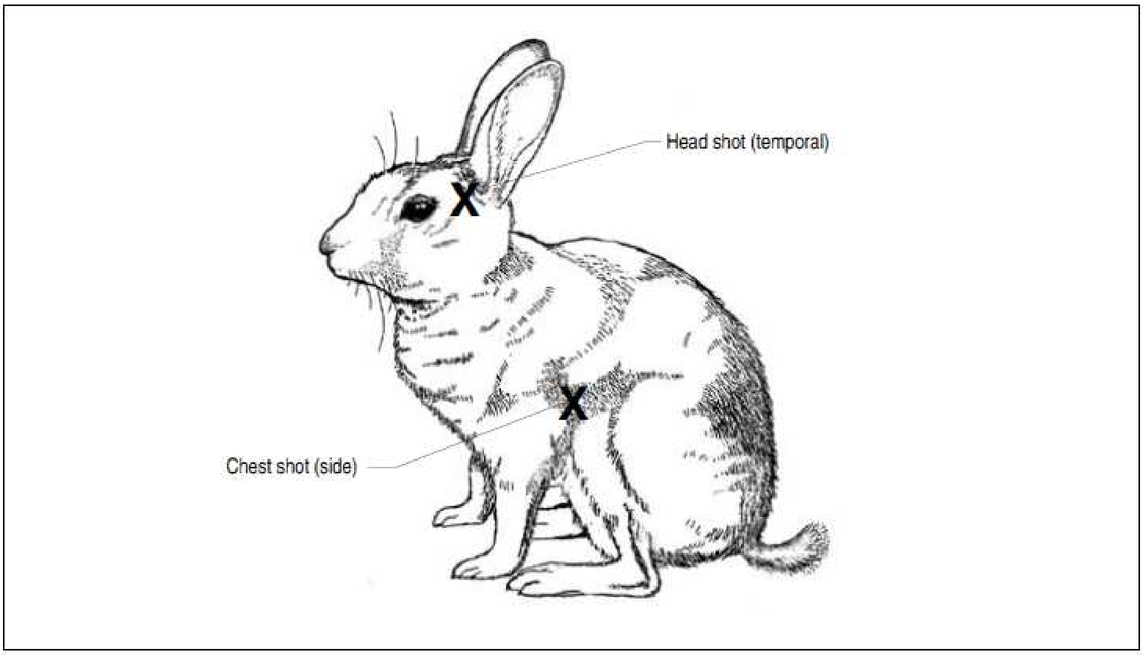

Diagram 2 - Side view (skeleton)

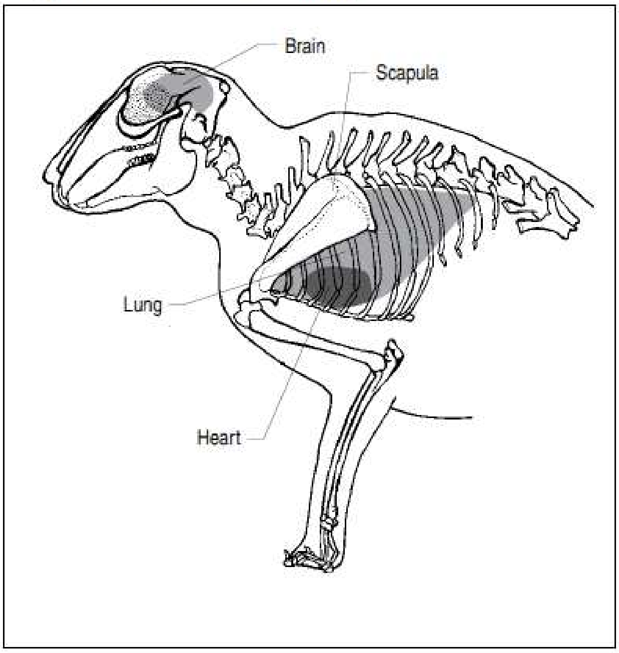

Diagram 3 - Head shot (frontal)

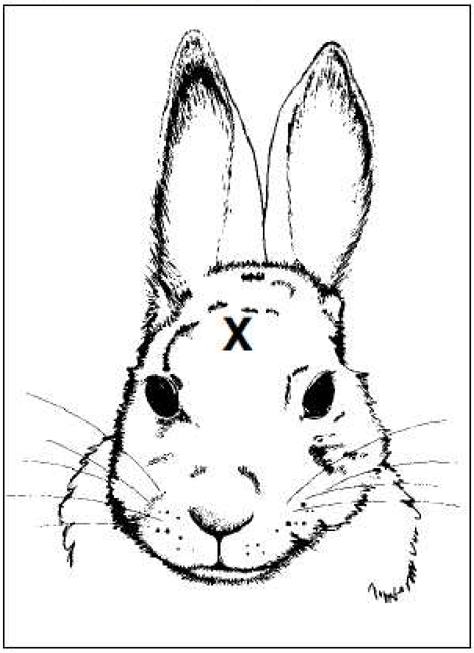

Supplement: S1 SOP — (PDF) [file pone.0146298.s001.pdf]
