# Supplementary material for: Assessing Animal Welfare Impacts in the Management of European Rabbits (Oryctolagus cuniculus), European Moles (Talpa europaea) and Carrion Crows (Corvus corone)
Source: PLoS One. 2016 Jan 4;11(1):e0146298. doi: 10.1371/journal.pone.0146298 (PMC4699632; doi:10.1371/journal.pone.0146298)
Supplement: S2 SOP — (PDF) [file pone.0146298.s002.pdf]

# Fencing rabbits from crops

## Background

Fencing is recommended as a humane and environmentally acceptable means of excluding European rabbits (*Oryctolagus cuniculus*) to reduce damage to agricultural, horticultural or forestry interests in the UK. Other rabbit control methods include shooting (including lamping), spring trapping, live trapping (cage or drop-box), snaring, ferreting, hunting with dogs, stalking or flushing with dogs and shooting, long-netting, gassing with phosphine, warren and harbour destruction, habitat management, chemical repellents, tree-guards and shelters, and repellents.

Permanent wire-mesh is traditionally used to deny rabbits access to vulnerable areas and while electric fencing is cheaper to purchase and erect, maintenance costs are greater. This Standard Operating Procedure (SOP) covers both wire-mesh and electric fencing for the protection of growing crops in an agricultural environment. This SOP is a guide only; it does not replace or override the legislation and should only be used subject to the applicable legal requirements.

## Application

• Owners or occupiers of land may be legally obliged to carry out rabbit control at any time of the year under the Pests Act 1954, the Agricultural Act 1947 and the Agriculture (Scotland) Act 1948. These are likely to apply where horticultural or agricultural crops require protection.
• Fencing is generally cost effective for large areas but expensive for protecting small areas. Fencing can be an effective method of protecting crops from rabbits but it can also be expensive to install. However, fencing has a useful lifetime of about 10 years and is often more cost-effective than control methods undertaken year after year. Some reduction of rabbit numbers may also be necessary if populations are high. A breach can put the whole planted area at risk.
• Electric fencing has a low capital cost and is reusable. Effective electric fencing is dependent on intensive checking and maintenance and requires a reliable power source and earthing; breach or loss of power renders the whole fence-line ineffective.
• Fencing may be particularly useful when burrows are difficult to access for gassing or terrain unsuitable for shooting. Fencing is useful where rabbit harbourage makes other techniques impractical, or when complete exclusion is required.

STANDARD OPERATING PROCEDURE

• Fences must be regularly inspected and repaired, and in the case of electric fencing vegetation needs to be regularly trimmed.

• The maximum manageable size of enclosure will depend on the number of rabbits present, but may be as small as 25 ha on the most heavily infested areas (40 rabbits per hectare or more). Ideally fencing would surround the entire vulnerable area or if that is not practical a strip fence extending ≥150 m beyond either end of the problem area may be used.

• Fences may be erected along the boundary between the field to be protected and the infested harbourage.

• Fencing should be combined with a reduction of rabbit harbourage.

• Where rabbit-proof fencing has been used to encircle an area or along the boundary between woodland and adjacent harbourage, cage traps can be used to remove rabbits remaining within that area or that subsequently breach the fence (around/under).

• Both traditional wire-mesh and electric fencing can be used to exclude rabbits. Temporary electric fences (netting or multi-strand strained wire systems) have recently become popular for crop protection.

• In trials, wire-mesh or electric netting fences were erected along woodland-field boundaries to exclude heavy infestations of rabbits in woodland from arable fields, and extended by 150m at each end after the crop had been planted. Both types of fence resulted in an 80% reduction in the number of rabbits counted in the protected fields. When correctly erected and maintained, wire-mesh and electric fences can reduce rabbit numbers on protected fields by 85% to 90%.

• Wire mesh is probably more suitable if year round protection is required for several years in the same place. Electric fencing may be more cost-effective and convenient if protection is required for part of the year, where the crop for protection will be grown in different places each year or where there are game interests, because it interferes less with pheasant drives and chick rearing.

• Electric fencing is cheaper to purchase and erect but maintenance costs are higher than for wire mesh fencing. Wire-mesh requires regular monthly inspections, whereas electric fencing needs inspecting every few days but can later be extended to 2-3 week intervals.

• Two types of electric fence are in common use: electric netting and electric strained wire (a scaled-down version of the kind more commonly used to manage sheep). Choosing between them is to some extent a matter of preference, although on balance strained-wire may be better. Electric netting is much quicker to erect and take down and about 5 to 10% cheaper, depending on the length to be erected. Strained-wire fences, by contrast, use materials which many farmers and growers may already have on the farm to manage domestic stock, which

would therefore make them cheaper than electric netting fences. They are also likely to last longer and can carry higher voltages over equivalent distances.

## Animal Welfare Considerations

### Impact on target animals

• Unlike some other control methods, fencing between harbourage and cropped fields will target only those rabbits attempting to enter the crop. Rabbits fenced into the field may need to be cage trapped.
• In a trial of electric netting and electric strained wire fencing to exclude rabbits, each type was tested at six different sites per year over six years, during which five rabbits were found entangled and killed in electric netting fences (which are close to the ground), but these problems were not observed with electric strained wire fencing.
• The impact on rabbits of electrocution from electric fencing appears small and transient. In experiments rabbits were observed to investigate electric fences by touch with their noses and on receiving a shock to move varying distances away from the fence. Avoidance of fences increased considerably over the first week but rabbits continued to approach fences and receive shocks throughout the 4 week trial period.
• Fencing of any kind, if effective at excluding rabbits, may have an effect on food availability, depending on the time of year, and the presence and proximity of alternative food sources. Fencing will exclude rabbits from some potential feeding areas but once established, long-term fencing will not create a sudden reduction of food availability. Erecting fencing before the crop is planted should help to minimise any food shortage effects.

### Impact on non-target animals

• Fencing is not target species-specific, unlike some other methods.
• In a trial, frogs, toads and hedgehogs were entangled and killed in electric netting rabbit fences (which are close to the ground), but these problems were not observed with electric strained wire rabbit fencing. Deaths were relatively uncommon except where fences were erected near to ponds containing frogs or toads and it may be advisable not to use electric fences close to such habitats.
• In trials testing electric strained-wire fencing with badgers, most learnt to avoid the fence following the night of erection and the fence had no effect on the length of time badgers were active at night or where they slept during the day.
• Wildlife such as deer can become entangled in electric netting if power is disconnected.

• Electric fences are lower than wire mesh so adult pheasants will fly over them more readily and chicks can pass through them

## Health and Safety Considerations

• Electric fence installation
  - o Electric fence controllers should conform to the current BS EN standards.
  - o Mains operated units should be installed by a competent electrical contractor and must be fitted with suitable electrical protection.
  - o Where possible avoid erecting in the vicinity of power lines.
  - o Electric fencing close to public right of ways should be fitted with warning notices (fences and supply box).
  - o For safety reasons, two energisers should never be attached to a single length of electric fence.

• While erecting wire fencing assess the area for the location of overhead lines, do not run the fence parallel to a power line as this could result in voltages being induced in the fence.

• If a fence has to be erected close to a power line, plan a route at right angles to the power line.

## Equipment Required

Wire mesh fencing

• 18 gauge, 31 mm hexagonal mesh. The wire mesh must conform to the British Standard and not just to the European DIN Standard. The latter specifies 31 mm mesh but allows a tolerance up to 36 mm which is too large to stop all but fully-grown rabbits. 19 gauge (= 1.0 mm diameter wire) netting is too lightweight and should not be used because rabbits can bite through the wire.

• 750-900 mm high plus 150 mm lapped on ground surface towards rabbit harbourage with turfs placed on the lapped netting at 1 m intervals to secure in place

• Two 2.65 mm high tensile spring steel straining wires

• Wooden stakes 1.8 m (≤m apart) to support straining wires

• End posts 2 m x 100 mm braced by struts 2 m x 80 mm

• Local site conditions or other considerations may demand a variation to these specifications

Electric fencing

• Electric netting
  - o Heavy-duty polythene twine mesh, horizontal strands interwoven with electrically conductive stainless steel wire

    - Between 0.5-0.75 m high and supplied in 25 m or 50 m rolls with fence posts already fitted
- Electric strained-wire system
    - Seven parallel electrified conducting wires (7-strand, 16 gauge medium tensile mild steel) at heights between 5-40cm above ground – top 6 wires live.
    - Metal stakes
    - Adjustable plastic insulators
    - Reel post for tensioning the whole system
- Energiser capable of producing an output of ≥1 joule when measured into a 500 ohm resistance
- Suitable leisure batteries
- Earth stake and connectors

Other Equipment:

- Tools
- Warning signs

# Procedures

## Assessment of site and estimation of rabbit numbers

- The site should be visited in advance to assess rabbit numbers, to check that the type of fence planned is appropriate and to decide the position of the fence. The proposed route of the fence should be inspected to confirm that it is feasible. The proximity of habitats such as ponds which may contain vulnerable non-target species should also be taken into account.
- Ideally a fence would be erected to surround the entire vulnerable area, but if this is not practical, a strip fence extending ≥150 m beyond either side of the area may be used.
- The maximum manageable size of enclosure will depend on the number of rabbits present, but may be as small as 25 ha on the most heavily infested areas (40 rabbits per hectare or more).
- Discuss any proposed changes to the specification with one of Natural England's Wildlife Management Advisers.

## Wire mesh fence erection

- Attach mesh to two 2.65 mm high tensile spring steel straining wires (bottom and top of fence) with galvanised fence rings so that fence is 750-900 mm high

STANDARD OPERATING PROCEDURE

• Lap 150 mm on ground surface towards rabbit harbourage with turfs placed on the lapped netting at 1 m intervals to secure in place

• Where rabbit numbers are high and they are known to be fence-climbing, or where it is especially important to prevent invasion by rabbits, the fence specification can be improved by projecting the top 150 mm of the fence outwards at 45° towards harbourage

• Support straining wires with wooden stakes up to 15 m apart.

• End posts 2 m x 100 mm, braced by struts 2 m x 80 mm, should be placed at the ends of the fence and at bends

• Gates in fence should be kept to a minimum as they create maintenance difficulties. Dig a wooden sill into the ground to prevent burrowing underneath

• Contact Natural England's Wildlife Management Advisors to discuss any proposed changes to the specification.

Electric fence erection

• Prior to erection, mow a 450-600 mm wide strip along the fence line or kill off vegetation using an approved herbicide to ensure that conducting wires are kept clear of vegetation that might short-circuit the system.

• Erect electric netting on posts included so that fence is 0.5-0.75 m high.

• Erect electric strained-wire fencing on metal stakes (≤7 m apart) using plastic insulators such that the seven wires are at heights between 5-40 cm from the ground, with the top six wires live. Tension the whole system using the reel post at the end of the fence.

• Earth the fence using stake and connectors.

• Connect and switch on energiser unit.

• Check that ≥2.5 kV is maintained throughout the fenceline.

• Adjust earthing if necessary.

• Erect warning signs if close to public access.

• For guidance on the use of electric fences contact Natural England's Wildlife Licensing Unit. Contact Natural England's Wildlife Management Advisors to discuss any proposed changes to the specification.

Fence maintenance

• Inspect wire mesh fences monthly and repair / maintain as necessary.

• Inspect electric fences initially every few days but later at 2-3 week intervals. Batteries need regular changing (a fully charged 70 Ah battery will need to be changed every 2 to 3 weeks). Check for short-circuits and that fence is maintaining voltage.

### Removal of remaining rabbits from fenced area

• Any rabbits remaining inside the fenced area can be cage trapped before the crop is planted. Some internal, sub-dividing fencing may be necessary to help in the removal of rabbits in heavily infested areas.

### Assessing effectiveness

• The effectiveness of fencing should be assessed by making rabbit counts in the crop edge after installing the fence for comparison with pre-fencing counts. This is probably best done at dawn or dusk and should be conducted regularly to ensure rabbits are not breaching the fence or going around the end of the fence where the vulnerable area is not completely enclosed by the fence. Rabbit counts could be combined with fence checking / maintenance visits.

• Contact Natural England's Wildlife Management Advisors for more information and advice on site assessment and monitoring of rabbit numbers.

## Procedural Notes

See *Safety for farming and horticulture* by Power Systems (http://www.sppowersystems.co.uk/safety/pdf/farmers_booklet.pdf).

## References

This SOP was adapted from RAB005 diffusion fumigation of rabbit warrens, prepared by Trudy Sharp (2012).

Defra (2005) *Report of the Independent Working Group on Snares*. http://archive.defra.gov.uk/wildlife-pets/wildlife/management/documents/snares-iwgs-report.pdf.

Forestry Authority (1998) *The Prevention of Rabbit Damage to Trees in Woodland*, FCPN002. http://www.forestry.gov.uk/PDF/fcpn2.pdf/$FILE/fcpn2.pdf.

Forestry Authority (1998) *The Prevention of Mammal Damage to Trees in Woodland*, FCPN003. http://www.forestry.gov.uk/PDF/fcpn3.pdf/$FILE/fcpn3.pdf.

Natural England (2011) *Rabbits: management options for preventing damage*, Technical Information Note 3. http://publications.naturalengland.org.uk/publication/19005?category=41004.

Natural England (2011) *Rabbits: use of cage-trapping to prevent agricultural damage*, Technical Information Note 24. http://publications.naturalengland.org.uk/publication/22011?category=41004.

Natural England (2011) *Rabbits: use of fencing to prevent agricultural damage*, Technical Information Note 23. http://publications.naturalengland.org.uk/publication/25007?category=41004.

McKillop, I.G., Butt, P., Lill, J., Pepper, H.W. and Wilson, C.J. (1998) Long-term cost effectiveness of fences to manage European wild rabbits. *Crop Protection*, **17**(5): 393-400.

McKillop, I.G., Ginella, S.G.V., Wilson, C.J., Hanlon, A.J. and Pugh, B.D. (1993) The effects of power failure on the behaviour of European wild rabbits at electric fences. *Applied Animal Behaviour Science,* **35**: 277-290.

McKillop I.G.,Pepper, H.W. & Wilson, C.J. (1988) Improved specifications for rabbit fencing for tree protection. *Forestry*, **61**(4): 359-368.

McKillop I. G., Phillips K. V. and Ginella S. G. V. (1992) Effectiveness of two types of electric fences for excluding European wild rabbits. *Crop Protection*, **11**: 279-285.

McKillop I.G. and Wilson C.J. (1987) Effectiveness of fences to exclude European rabbits from crops. *Wildlife Society Bulletin*, **15**: 394-401.

McKillop I.G. and Wilson C.J. (1999) The behaviour of free-living European wild rabbits at electric fences. *Crop Protection,* **18**: 193-197.

Poole D.W., McKillop I.G. (1999) Comparison of the effectiveness of two types of electric fences to exclude badgers. *Crop Protection*, **18**(1): 61-66.

Power Systems (unknown date) *Safety for farming and horticulture*. http://www.sppowersystems.co.uk/safety/pdf/farmers_booklet.pdf

Sharp T (2012) RAB005 diffusion fumigation of rabbit warrens; Standard operating procedure. Invasive Animals Co-operative Research Centre, Australian Government. http://www.feral.org.au/wp-content/uploads/2013/08/RAB005_fumigation.pdf
