# Supplementary material for: Assessing Animal Welfare Impacts in the Management of European Rabbits (Oryctolagus cuniculus), European Moles (Talpa europaea) and Carrion Crows (Corvus corone)
Source: PLoS One. 2016 Jan 4;11(1):e0146298. doi: 10.1371/journal.pone.0146298 (PMC4699632; doi:10.1371/journal.pone.0146298)
Supplement: S3 SOP — (PDF) [file pone.0146298.s003.pdf]

Spring trapping moles

Background

Spring trapping is the most widely used method of controlling moles in the UK now that strychnine poison is no longer approved for this purpose. Spring traps are favoured for controlling mole activity on farms and on amenities such as golf courses, racecourses, sports fields, parks and ornamental gardens. Spring trapping and fumigation using phosphine are the mole control methods suggested by Natural England. Alternatives include using pesticides approved for controlling earthworms and other soil invertebrates to reduce the food available for moles (this is allowed on managed amenity turf only), sonic deterrents or live-trapping and translocation. However there is no evidence that sonic deterrents are effective and Natural England does not recommend the relocation of live-trapped moles on welfare grounds. No poisons or repellents are approved for mole control.

Moles are fossorial and live and feed in an underground network of feeding tunnels. Mole traps are set in these underground tunnels and are designed to catch moles around the body when a trigger plate or wire is pushed, releasing the killing mechanism. Mole traps do not currently require approval in the UK, but this Standard Operating Procedure (SOP) assumes that existing mole traps meet the minimum standard for approval in other spring traps. This SOP is a guide only; it does not replace or override the legislation and should only be used subject to the applicable legal requirements.

Application

• Spring-trapping is thought to be one of the most effective methods of controlling moles when conducted by an experienced operator in small-scale applications. However, the deployment of mole traps can be labour-intensive.
• Effective trapping relies on locating suitable runs.
• Two main types of spring trap are available in the UK, the scissor (pincer) trap and the Duffus (half-barrel) trap. These offer different advantages in terms of ease of setting and monitoring, visibility above ground, and suitability for use in different situations. A third type, the talpex-style trap, has become available more recently too.

• Scissor traps are best set in deep tunnels and are difficult to set in loose soil or shallow runs. A scissor trap consists of a pair of spring-loaded jaws which, when the trap is set, are held apart by a metal trigger plate. When the plate is disturbed by a mole it drops out of place, allowing the jaws to come together, to catch the mole around the thorax and crushing it to death.

• Duffus traps can be set in loose soil or shallow runs. The trap consists of a half-barrel of metal from which are suspended two spring-activated wire catching-loops and two triggers. Two moles may be caught in the same trap, one at each end. When a mole touches one of the triggers the related catching loop is drawn up tightly around the body of the mole.

• Trapping can be targeted where damage arises and it is relatively safe for non-targets, users and other people.

• Mole control can be carried out at any time of year, however, moles are most noticeable between October and April when they are actively digging new tunnels, and it is recommended that mole control takes place during this period. This will target the individuals that are most active.

• There is a strong bias towards male captures in winter and spring, and reproduction in mammals is generally limited by the number of breeding females. Therefore, a long-term reduction in mole numbers might be best achieved by trapping during April and May, when females are most likely to be pregnant or lactating, and after the breeding peak in male activity - trapping earlier is likely to result in mainly male captures.

Animal Welfare Considerations

Impact on target animals

• Previous research questions the humaneness of spring-traps, and they are banned in some US states on welfare grounds.

• The Pests Act 1954 made it an offence, in England, Wales and Scotland, to use a spring trap for killing or taking animals, other than one approved by an Order of the Secretary of State. However, mole spring traps are exempt under The Small Ground Vermin Traps Order 1958.

• Traps for other species which do require approval are approved if 80% of twelve tests cause irreversible unconsciousness in the target animal within 5 minutes.

• Any mole caught in a trap becomes a Protected Animal under the Animal Welfare Act 2006. The person deemed responsible for a Protected Animal is obliged not to cause it unnecessary suffering which could reasonable have been avoided or reduced. An offence is committed whether through an act, or a failure to act, and it is also an offence not to provide for an animal’s needs, such as food, environment and protection from pain, suffering, injury and disease.

STANDARD OPERATING PROCEDURE

Because a trapped mole may not necessarily be killed quickly (for example if it is caught by a limb), traps need to be visited regularly. Defra recommends that spring traps for rats and mice are visited at least once per day, although more frequent visits are recommended for moles because they have a high metabolic rate.

• Moles are thought to die relatively quickly in spring traps, most likely through acute haemorrhage leading to acute haemorrhagic shock. However because mole traps do not require approval, the time to irreversible unconsciousness and death have not been measured.

• Any trapped moles found alive should be humanely dispatched as soon as possible.

• Moles can legally be trapped at any time of year. There are welfare implications of trapping while females have dependent young because this results in young moles being left to starve to death. If lactating females are caught in a trap, reasonable efforts should be made to humanely destroy dependent young, otherwise they will die a slow death from starvation.

• The welfare impact of mole trapping could be reduced by implementing a close season of 2-3 months during which mole control was banned to protect breeding females and their dependent young. However such a ban would effectively mean an embargo on control at what might be the most effective time of year for long-term population reduction.

Impact on non-target animals

• Because mole spring traps are used beneath ground within mole tunnel systems, they are likely to be very species-specific than other spring traps. However it is possible that individuals of other small mammal species using mole tunnels might be killed or injured by a mole trap.

• If a trapped mole is eaten by a predator, there is no secondary threat to the predator as is the case with poisoning. However it is possible that a non-target animal could be injured in a trap while digging in the vicinity of a set trap.

• Live non-target animals caught in traps must be examined for injuries and signs of illness or distress and dealt with as follows:

  - Animals which are unharmed or have only received minimal injuries such as minor cuts or abrasions should be immediately released at the site of capture.
  - Animals which have more severe injuries or which are suffering from thermal stress should either be euthanised (unless protected) or should receive appropriate attention. An animal suffering from thermal stress can initially be placed in a suitable quiet holding area which provides warmth or shade to allow recovery before release. Animals with treatable injuries that cannot be immediately released or those failing to recover from thermal stress should be presented to a veterinarian or a registered wildlife carer for treatment.

- Animals that have injuries which are untreatable or which would compromise their survival in the wild should be euthanased using a technique that is suitable for the species.

• If a domestic pet is caught, it should be taken to the nearest vet, animal shelter or council pound where it can be examined for injuries, scanned for a microchip and the owner contacted, or assessed for suitability for re-homing.

Health and Safety Considerations

• Operators should be wary of the risks of injury when placing and setting traps. Protective leather gloves may help prevent injuries from trap jaws but may also hinder trap-setting.

• Operators must be protected by tetanus immunisation in case of infection of scratches and bites.

• Good personal hygiene is encouraged when handling wild animals. Routinely wash hands and other skin surfaces contaminated with faeces, blood and other body fluids.

Equipment Required

Spring traps

• Scissor, Duffus or talpex-style mole traps are available from agricultural suppliers, pest control merchants, garden centres, ironmongers and online.

• Traps should be well-maintained, not rusty and should operate smoothly and swiftly when triggered.

Other Equipment

• Probe for locating burrows, e.g. a 10mm x 600 mm steel bar or large screwdriver

• Pliers for adjusting traps.

• Knife for cutting roots etc.

• Trowel.

• Runner for smoothing tunnel floor before positioning trap.

Procedures

Locating mole runs

• Effective trapping relies on locating suitable runs. These are the main tunnels which are usually at least 150 mm below ground.

STANDARD OPERATING PROCEDURE

• Identify potential run positions and push the probe into the ground slowly and firmly. A sudden ‘give’ will be felt when the probe enters a run.

• Once a suitable run is identified, probe around it to determine its direction; straight sections of run are best.

Setting and placing traps

• Rub soil into hands before starting to help mask human odours.

• Carefully pre-set traps according to the manufacturer’s instructions before beginning to dig them in.

• Once a suitable run has been located, use a trowel or knife to dig a hole the size of the trap in the roof of the run. Remove loose soil or other material taking care not to disturb the tunnel more than necessary. Smooth sides and floor of a tunnel indicate it is in use. Use a runner or hand to smooth down the sides and floor before positioning the trap.

• Align the set trap with the bore of the tunnel and place it into the hole with the lowest part of the mechanism lightly pressed into the tunnel floor.

• Exclude light from around the trap using turf, vegetation and soil. Do not let this material fall into the tunnel or prevent the trap from operating.

• Traps should be checked at least once a day to reset any that are sprung and to remove dead moles.

• Continue trapping until all mole activity in the area ceases.

• Contact Natural England’s Wildlife Management Advisors for more information and advice on site assessment and monitoring of mole numbers.

Assessing effectiveness

• Revisit the site regularly to check for new activity. Flattened molehills and press down surface tunnels; this will make it easier to detect the continuing presence of moles.

References

This SOP was adapted from RAB008 trapping of rabbits using padded-jaw traps, prepared by Trudy Sharp (2012).

Atkinson, R.P.D., Macdonald, D.W. & Johnson, P.J. (1994) The status of the European mole Talpa europea L. as an agricultural pest and its management. *Mammal Review*, **24**(2): 73-90.

Baker, S.E., Shaw, R.F., Atkinson, R.P.D. & Macdonald, D.W. (in prep) The welfare implications of kill-trapping European moles (Talpa europaea) in Britain: a post-mortem study.

Defra (2009) *Code of Practice for the prevention and control of rodent infestations on poultry farms*. (http://archive.defra.gov.uk/foodfarm/farmanimal/diseases/atoz/zoonoses/documents/reports/salrodent.pdf).

Gorman, M.L. & Stone, R.D. (1990) *The Natural History of Moles*. Helm, London.

Natural England (2011) *Moles: options for management and control*. *Technical Information Note TIN033*. http://publications.naturalengland.org.uk/publication/34015?category=41004.

Nicholls, J. (2010) *Mole Catching; A Practical Guide*. Crowood, Marlborough, UK.

Quy, R. & Poole, D. (2004) *A review of methods used within the European Union to control the European mole,* Talpa europea*.* Defra. http://www.naturalengland.org.uk/Images/molereview_tcm6-4393.pdf.

Rudge, A.J.B. (1963) A study of mole-trapping. *Proceedings of the Zoological Society of London*, **149**, 330-334.

Sharp T (2012) RAB008 trapping of rabbits using padded-jaw traps; Standard operating procedure. Invasive Animals Co-operative Research Centre, Australian Government. http://www.feral.org.au/wp-content/uploads/2013/08/RAB008_trapping-rabbits.pdf
