# Supplementary material for: Assessing Animal Welfare Impacts in the Management of European Rabbits (Oryctolagus cuniculus), European Moles (Talpa europaea) and Carrion Crows (Corvus corone)
Source: PLoS One. 2016 Jan 4;11(1):e0146298. doi: 10.1371/journal.pone.0146298 (PMC4699632; doi:10.1371/journal.pone.0146298)
Supplement: S4 SOP — (PDF) [file pone.0146298.s004.pdf]

Live trapping and relocating moles

Background

Moles are reported to be pests on farms, amenities and gardens in the UK. Spring trapping and phosphine gassing are the mole control methods most widely used on farms and amenities. However, there is evidence that some amenity managers and domestic gardeners in particular are interested in live-trapping and relocating moles as a non-lethal alternative, but Natural England does not recommend the relocation of live-trapped moles on welfare grounds. Other non-lethal alternatives include using pesticides approved for controlling earthworms and other soil invertebrates to reduce the food available for moles (this is allowed on managed amenity turf only) or sonic deterrents, but there is no evidence that sonic deterrents are effective. No repellents are approved for mole control.

Moles are fossorial and live and feed in an underground network of feeding tunnels. Live capture mole tube traps are designed to be set in these tunnels and to catch moles alive when they push into the trap through a one-way swing door. Such traps do not incorporate a spring and so would be outside the spring traps approval legislation even if mole spring traps did require approval. This Standard Operating Procedure (SOP) is for live-trapping with a plastic mole tube trap followed by relocation to another site. This SOP is a guide only; it does not replace or override the legislation and should only be used subject to the applicable legal requirements.

Application

• Plastic mole tube traps are now available, often marketed as ‘humane’ mole traps. Each trap consists of two short plastic tubes which fit together to produce a tube approximately 26 cm long. There is a light metal swing door at either end allowing the trap to be entered from either end. The doors are hinged, allowing passage in one direction only – into the trap. Tube traps are set underground, in mole runs, in a similar way to mole spring traps.

• Because tube traps do not incorporate a spring they are not covered by the spring traps approval process. If they did have a spring they would anyway be exempt from the process under the The Small Ground Vermin Traps Order 1958, along with all mole spring traps, lethal or non-lethal.

• Effective trapping relies on locating suitable runs.
• Moles can be relocated using a soft-release method but identification of a suitable site that is not already inhabited by moles and at which moles are welcome is often difficult.
• Trapping can be targeted where damage arises and it is safe for non-targets, users and other people.
• Mole control can be carried out at any time of year, however, moles are most noticeable between October and April when they are actively digging new tunnels, and it is recommended that mole control takes place during this period. This will target the individuals that are most active.
• There is a strong bias towards male captures in winter and spring, and reproduction in mammals is generally limited by the number of breeding females. Therefore, a long-term reduction in mole numbers might be best achieved by trapping during April and May, when females are most likely to be pregnant or lactating, and after the breeding peak in male activity - trapping earlier is likely to result in mainly male captures.

Animal Welfare Considerations

Impact on target animals

• Any mole caught in a trap becomes a Protected Animal under the Animal Welfare Act 2006, making it an offence to cause unnecessary suffering. The person deemed responsible for a Protected Animal is obliged not to cause it unnecessary suffering which could reasonably have been avoided or reduced. It is also an offence not to provide for an animal's needs, such as food, environment and protection from pain, suffering, injury and disease. An offence is committed whether through an act, or a failure to act.
• Moles are sometimes known to die in Friesian live-traps (which have a nest chamber), even when provisioned with bedding and food.
• Tube traps have internal dimensions of 22 cm long and 48 mm diameter. There is no room inside to provide adequate bedding. Being small with a high metabolic rate moles are susceptible to chilling. Because tube traps are narrow they allow little movement and normal behaviour and interactions will be prevented while the mole is in the trap. The one-way swing doors at either end of a tube trap potentially allow more than one mole to become trapped together; this might lead to fighting which could cause injury or death.
• Moles are insectivores and exhibit three bouts of feeding and other activity in a 24-hour period, so traps should be provisioned with suitable food, e.g. earthworms.
• Trapping should be avoided in very cold weather conditions.

STANDARD OPERATING PROCEDURE

• The Animal Welfare Act does not specify how frequently live traps should be checked. However, The British Association for Shooting and Conservation (BASC) advises that cage traps for mammals should be inspected at least once a day. Elsewhere more frequent visits have been recommended, e.g. every 4 hours, as moles have a high metabolic rate and may die if left in a trap without food for any length of time.

• Natural England does not recommend the relocation of moles on welfare grounds. Moles are highly territorial and release into another mole's territory may result in fighting between individuals and even death. Release into an area with no existing run system exerts considerable pressure on the mole to obtain sufficient food to survive and may be an offence under the Animal Welfare Act 2006. It is therefore recommended that if a mole is captured it is humanely dispatched.

• The welfare impact of mole trapping could be reduced by implementing a close season of 2-3 months during which mole control was banned to protect breeding females and their dependent young. However such a ban would effectively mean an embargo on control at what might be the most effective time of year for long-term population reduction.

• A ‘soft’ release approach, including measures to assist animals in adapting to a new environment, increases the success of relocations. A soft approach is particularly important for moles because their biology and behaviour is not well adapted to being relocated. If live trapped moles are to be relocated they should be taken to a pre-prepared release site using a soft release method involving provision of a nest chamber with bedding and sufficient food for 4 days.

Impact on non-target animals

• Because mole traps are used underground within mole tunnel systems, they are highly species-specific in terms of capture, and safe for predators or people that might dig them up.

• Moles can legally be trapped at any time of year. There are welfare implications of trapping while females have dependent young because this results in young moles being left to starve to death. If lactating females are caught in a trap, reasonable efforts should be made to humanely destroy dependent young, otherwise they will die a slow death from starvation.

• Live non-target animals caught in traps must be examined for injuries and signs of illness or distress and dealt with as follows:

- Animals in good condition should be immediately released at the site of capture.
- Animals suffering from hunger, dehydration or thermal stress should either be euthanised (unless protected) or should receive appropriate attention.

Health and Safety Considerations

• Operators must be protected by tetanus immunisation in case of infection of scratches and bites.

• Good personal hygiene is encouraged when handling wild animals. Routinely wash hands and other skin surfaces contaminated with faeces, blood and other body fluids.

Equipment Required

Mole tube traps

• Plastic tube traps are available from pest control merchants, garden centres and online.

Other trapping equipment

• Probe for locating burrows, e.g. a 10mm x 600 mm steel bar or large screwdriver
• Pliers for adjusting trap doors.
• Knife for cutting roots etc.
• Trowel.
• Runner for smoothing tunnel floor before positioning trap.
• Earthworms.

Release site preparation equipment

• Spade for digging release chamber.
• Hay for bedding.
• Small piece of plywood.
• Probe as above for making access tunnel.

Relocation and release equipment

• Bucket with tight-fitting cover.
• Hay for cover during transport.
• Earthworms.

Procedures

Selection and setting up of the release site

• Release sites should be fully prepared before attempting to catch a mole for relocation.

STANDARD OPERATING PROCEDURE

• Sites should be selected carefully such that they are not already occupied by a mole, they provide suitable conditions for moles and the owner / occupier has given their permission to release the mole there.

• To maximise the suitability of release sites, select points that are close to an area with molehills present and with similar soil type and vegetation, but that are not within 20 m of a molehill.

• The availability of suitable mole food at a proposed release site can be checked by digging a number of quadrats (25 cm x 25 cm to a depth of 30 cm) at both the trapping and proposed release sites. The soil should be removed and sieved, and the number of earthworms and other soil invertebrates counted at each site. Food availability can then be compared between the trapping and release sites.

• Once a suitable site has been chosen, a soft-release chamber can be constructed in the following way. Dig down approximately 35 cm to avoid frost. At the base of the hole, create a nest chamber 15-20 cm across, with three small chambers scooped out from the sides. Fill the main chamber with clean, dry hay. Worms can be provided later when a mole is released into the chamber. Cover the chamber with a small piece of plywood and replace the soil on top. Create an access route at an angle between the chamber and the soil surface using a probe or metal bar to facilitate exit should the mole choose to forage on the surface or escape.

Trapping

• Locating mole runs
  - Effective trapping relies on locating suitable runs. These are the main tunnels which are usually at least 150 mm below ground.
  - Identify potential run positions and push the probe into the ground slowly and firmly. A sudden ‘give’ will be felt when the probe enters a run.
  - Once a suitable run is identified, probe around it to determine its direction; straight sections of run are best.

• Setting and placing traps
  - Rub soil into hands before starting to help mask human odours.
  - Fit the parts of the tube trap together and place ten earthworms inside. Worms should be cut in half.
  - Once a suitable run has been located, use a trowel or knife to dig a hole the size of the trap in the roof of the run. Remove loose soil or other material taking care not to disturb the tunnel more than necessary. Smooth sides and floor of a tunnel indicate it is in use. Use a runner or hand to smooth down the sides and floor before positioning the trap.

- Align the set trap with the bore of the tunnel and place it into the hole with the lower side pressed lightly into the tunnel floor.
- Exclude light from around the trap using turf, vegetation and soil. Do not let this material fall into the tunnel or prevent the doors from operating.
- Traps should be checked at least every 4 hours.
- Continue trapping until mole activity in the area ceases.
- Contact Natural England's Wildlife Management Advisors for more information and advice on site assessment and monitoring of mole numbers.
- When a mole is caught either dispatch it humanely or place it quickly into a bucket (one mole per bucket) containing hay and a few earthworms. Quickly secure the tight-fitting lid and transport the bucket quickly and quietly to the prepared release site.

Release

• Open the lid of the release chamber. Place ten earthworms in each side chamber and ten in the main chamber. Worms should be cut in half.

• Quickly and quietly release the mole into the chamber and immediately replace the plywood cover and soil.

Assessing effectiveness

• Revisit the trapping site regularly to check for new activity. Flatten molehills and press down surface tunnels; this will make it easier to detect the continuing presence of moles.

• Revisit the release site regularly to monitor the appearance of molehills. After two weeks, if there is no sign of a territory being established, re-open the nest chamber and investigate for signs of tunnelling out or the presence of a dead mole.

References

This SOP was adapted from RAB008 trapping of rabbits using padded-jaw traps, prepared by Trudy Sharp (2012).

Atkinson, R.P.D., Macdonald, D.W. & Johnson, P.J. (1994) The status of the European mole Talpa europea L. as an agricultural pest and its management. *Mammal Review*, **24**(2): 73-90.

Baker, S.E. & Macdonald, D.W. (2012) Not so humane mole tube traps. *Animal Welfare*, **21**(4): 613-615.

British Association for Shooting and Conservation (????) *Trapping Pest Mammals*. (http://www.basc.org.uk/en/codes-of-practice/trapping-pest-mammals.cfm)

Gorman, M.L. & Stone, R.D. (1990) *The Natural History of Moles*. Helm, London.

Massei, G., Quy, R.J., Gurney, J. & Cowan, D.P. (2010) Can translocations be used to mitigate human-wildlife conflicts? *Australian Journal of Wildlife Research*, **37**: 428-439.

Morris, P. (1966) The mole as a surface dweller. *Journal of Zoology,* **149**: 46-49.

Natural England (2011) *Moles: options for management and control*. *Technical Information Note TIN033*. http://publications.naturalengland.org.uk/publication/34015?category=41004.

Nicholls, J. (2010) *Mole Catching; A Practical Guide*. Crowood, Marlborough, UK.

Quy, R. & Poole, D. (2004) *A review of methods used within the European Union to control the European mole,* Talpa europea. Defra. http://www.naturalengland.org.uk/Images/molereview_tcm6-4393.pdf.

Rudge, A.J.B. (1963) A study of mole-trapping. *Proceedings of the Zoological Society of London*, **149:** 330-334.

Rudge, A.J.B. (1966) catching and keeping live moles. *Journal of Zoology*, **149**(1): 42-45.

Shaw, R.F., Baker, S.E., Harrington, A.L., Macdonald, D.W. & Atkinson, R.P.D. (in prep) Preliminary observations on translocation as a method of non-lethal mole (*Talpa europaea*) control.

Sharp T (2012) RAB008 trapping of rabbits using padded-jaw traps; Standard operating procedure. Invasive Animals Co-operative Research Centre, Australian Government. http://www.feral.org.au/wp-content/uploads/2013/08/RAB008_trapping-rabbits.pdf
