# Supplementary material for: Assessing Animal Welfare Impacts in the Management of European Rabbits (Oryctolagus cuniculus), European Moles (Talpa europaea) and Carrion Crows (Corvus corone)
Source: PLoS One. 2016 Jan 4;11(1):e0146298. doi: 10.1371/journal.pone.0146298 (PMC4699632; doi:10.1371/journal.pone.0146298)
Supplement: S5 SOP — (PDF) [file pone.0146298.s005.pdf]

STANDARD OPERATING PROCEDURE

Managing molehills and tunnels

Background

Moles are reported to be pests on farms, amenities and gardens in the UK. Moles are fossorial animals, each living and feeding in an underground network of tunnels. Moles produce molehills (or spoil-heaps) as they excavate soil during production of tunnels and nest chambers. These molehills and tunnels are sometimes considered to be damage - financial, aesthetic or otherwise. Where control of any sort is considered necessary, the choice of method will be at least partly determined by the motivation for control. Moles are highly territorial, occupying exclusive individual territories that range between 300 and 3,000 m^2, depending on habitat quality. During the breeding season, however, male moles leave their home territories in search of mating opportunities and for a short while molehills may appear in places where they do not normally occur.

If moles are removed from an area, eg by trapping or phosphine fumigation, this might achieve a short-term population reduction, but new individuals, eg dispersing juveniles, are likely to invade the vacant territories at some point in the future. In some circumstances removal of moles may not be justified or necessary at all, and there may be ways of mitigating mole damage indirectly. In many cases, simply flattening or removing molehills or raised surface tunnels may provide a satisfactory solution, for example, where temporary removal of the molehills is adequate (eg to reduce risk of silage contamination with soil bacteria during silage cutting), where new molehills appear during the breeding season or where mole activity occurs where some damage can be tolerated. This Standard Operating Procedure (SOP) covers the management of molehills and tunnels, without attempting to manage the moles themselves. This SOP is a guide only; it does not replace or override the legislation and should only be used subject to the applicable legal requirements.

Application

• Killing moles in an effort to reduce perceived mole damage may not much affect mole numbers in the long-term or mole damage in the short-term. In some cases non-lethal ways of managing the perceived damage itself may be all that is needed, and may sit better with the ethos of the landowner or landowning organisation.

• Harrowing, rolling or otherwise managing mole hills and surface mole tunnels may be used to mitigate conflict without removing or killing the moles themselves. Removing mole damage in this way will not affect mole population sizes but if carefully targeted may achieve the desired outcome.
• Harrowing and rolling agricultural fields where moles are present, in grass being grown for silage or hay, may help to reduce contamination of the crop with soil bacteria. Molehill production is at its peak in spring, and treatment needs to take place late enough to destroy the majority of molehills that have accumulated in spring and early enough to allow the crop to recover before being cut. Rolling a growing silage crop provides additional benefits, such as increased tillering. Soil contamination of silage or hay can be further reduced by raising the cutting blades to avoid 'scalping' the soil surface and allowing the cut grass time to wilt before baling.
• Harrowing and rolling, or otherwise managing, molehills and surface tunnels is also used on some amenity grassland to reduce the visible signs of mole activity and potential risks of injury to people and animals.
• Molehills may also be managed in domestic gardens, either for aesthetic reasons, or to facilitate mowing without blunting mower blades on molehill soil and associated stones, or to make a level surface for walking or playing.
• Where the area to be treated is relatively small, it may be better to remove molehill soil and redistribute it over a wider area of grass, or use it elsewhere (it is reputed to be good for growing potted plants), than to attempt to flatten molehills directly. Where molehill soil is to be redistributed, this can be mixed with lawn sand and carefully brushed in as a top-dressing.
• The soil in a molehill will have come from the excavation of a length of tunnel underground, and so attempting to flatten it is likely to leave a raised area. Flattening or rolling molehills / tunnels will also compact the soil, which may then need aeration and reseeding. Instead, molehill soil can either be lifted off with a shovel or hosed back down the adjoining tunnel. However the latter is likely to result in the mole re-excavating its tunnel and making a new hill in a short space of time. If the tunnel system beneath ground can be left intact, there is less likelihood of new damage appearing straight away. Where surface tunnels cannot be tolerated they can be gently trodden or rolled down.
• If molehills are allowed to remain intact on top of grass, this may kill the turf underneath in a few days.

Animal Welfare Considerations

Impact on target animals

• Using simple measures, eg rolling, harrowing etc, to flatten molehills and surface tunnels, is considered a relatively benign, non-lethal method of managing mole damage. There is potential risk of moles being injured during harrowing, rolling or other flattening treatment, if they are very close to the surface or above ground during treatment. However they are most likely to be underground and to retreat to deeper parts of the tunnel system and therefore be unharmed.
• Where a feeding tunnel network is damaged, the mole will have to re-excavate the tunnel and could potentially suffer restricted food supplies until this is complete. Moles are insectivores with a high metabolic rate, and if a very large proportion of its feeding tunnel system is destroyed the mole could potentially starve before re-excavating it.
• If a mole was driven out of its territory, or forced above ground, during the harrowing or rolling treatment, it could potentially be killed by another mole, or starve to death.

Impact on non-target animals

• Harrowing, rolling or other flattening work must not take place over or close to badger setts, as interfering with a badger sett is illegal.
• Young moles could potentially be injured if the soil above their nest is heavily flattened, or they may be cut off from their mother if serious tunnel damage occurs, and starve as a result.

Health and Safety Considerations

• Harrowing or rolling work involving heavy machinery, eg on agricultural or amenity land, should only be conducted by trained and experienced operators, and the usual precautions should be taken regarding the safety of operators, other people and animals.
• Sensible precautions should also be taken when conducting small-scale molehill management by hand.

Equipment Required

Large-scale management of moles in a silage or hay crop

• Tractor
• Harrow
• Roller

Large-scale management of moles on amenity grassland

• Tractor and roller, or sit-on roller

Small-scale management of moles in lawns

• Shovel or spade

Procedures

Assessment of site and damage

• Walk the site to determine extent and scale of damage.

• Decide which areas require treatment and which approach to use.

• Contact Natural England's Wildlife Management Advisors for more information and advice on site assessment and monitoring of mole activity.

Large-scale management of moles in a silage or hay crop

• Harrow the grass crop early in the season to break up molehills and tunnels on the soil surface and to bring to the surface any partly exposed large stones.

• Follow this by rolling, when soil conditions allow, flattening any raised soil and pushing stones into the soil.

• Roll as necessary thereafter.

• Ideally this work will be conducted late enough to remove the molehills and tunnels that have accumulated over the winter / spring, when peak molehill activity occurs, but not too late to allow the grass to recover before cutting.

Large-scale management of moles on amenity grassland

• Harrow and then roll the affected area after the peak in mole activity, to remove the molehills and tunnels that have accumulated over the winter / spring.

• Roll as necessary thereafter.

Small-scale management of moles in lawns

• Carefully lift off molehill soil using a shovel or spade, and either redistribute it thinly over a wider area of grass, or use it elsewhere.

• Where any surface tunnels cannot be tolerated, gently tread these down.

Assessing effectiveness

• Revisit sites 4-7 days after treatment. Check for signs of new or continued activity. Always walk the perimeter of the treated area, including any adjacent banks, woods or fields, looking for fresh workings, as these areas provide the earliest evidence of re-infestation. New activity should be treated until no more occurs.

Procedural notes

• More detailed information on diffusion fumigation using phosphine can be found on approval labels, in HSE Agriculture Information Sheet No 22 Gassing of rabbits and vertebrate pests and relevant legislation.

References

This SOP was adapted from RAB005 diffusion fumigation of rabbit warrens, prepared by Trudy Sharp (2012).

Baker et al. (in prep) Moles and mole management in Britain; a questionnaire survey.

EBLEX (2011) Making grass silage for better returns; beef and sheep BRP manual 5. Better Returns Programme

Gorman, M.L. & Stone, R.D. (1990) *The Natural History of Moles*. Helm, London.

Lodal J. (1999) *The mole: a matter of control or conservation?* In: P.D. Cowan & C.J. Feare (eds) Advances in Vertebrate Pest Management. pp 43-56. Filander Verlag, Furth, Germany.

MAFF (1977) *Silage*. ADAS Bulletin 37, HMSO, London.

MAFF (1986) *Ensiling Grass*. ADAS Pamphlet 482, HMSO, London.

Natural England (2011) *Moles: options for management and control. Technical Information Note TIN033*. http://publications.naturalengland.org.uk/publication/34015?category=41004.

Quy, R. & Poole, D. (2004) *A review of methods used within the European Union to control the European mole,* Talpa europea. Defra. http://www.naturalengland.org.uk/Images/molereview_tcm6-4393.pdf.

Sharp T (2012) RAB005 diffusion fumigation of rabbit warrens; Standard operating procedure. Invasive Animals Co-operative Research Centre, Australian Government. http://www.feral.org.au/wp-content/uploads/2013/08/RAB005_fumigation.pdf
