# Supplementary material for: Assessing Animal Welfare Impacts in the Management of European Rabbits (Oryctolagus cuniculus), European Moles (Talpa europaea) and Carrion Crows (Corvus corone)
Source: PLoS One. 2016 Jan 4;11(1):e0146298. doi: 10.1371/journal.pone.0146298 (PMC4699632; doi:10.1371/journal.pone.0146298)
Supplement: S6 SOP — (PDF) [file pone.0146298.s006.pdf]

Shooting corvids

Background

Corvids, such as rooks and crows, may cause agricultural or horticultural damage by feeding on cereals, fruit and other crops. Corvids may be shot or cage-trapped, either in Larsen traps or multi-catch traps, either for the purpose of preventing serious damage to crops, vegetables, fruit, foodstuffs for livestock and certain other enterprises, or for preventing the spread of disease. Alternative methods of corvid management include visual or auditory scarers, habitat manipulation, diversionary feeding and exclusion netting.

The aim of shooting is to reduce corvid numbers in order to minimise damage. Shooting is used either to reduce bird numbers directly through killing or as part of a scaring or dispersal strategy. Shooting may have short-term advantages but the technique is often labour intensive, opportunistic and may have limited value in bird control. Corvids may be shot using a rifle, a shotgun or an air rifle. However, this Standard Operating Procedure (SOP) covers shooting corvids with a shotgun, which is the most suitable approach. This Standard Operating Procedure (SOP) is a guide only; it does not replace or override the legislation and should only be used subject to the applicable legal requirements.

Application

• All wild birds, their eggs and nests are protected under The Wildlife and Countryside Act 1981. However, there is a General Licence under the Act (WML - GL04), which allows crows, rooks, jackdaws, magpies and jays to be killed or taken to prevent serious damage or disease. This may only be undertaken by an ‘authorised person’ under the licence. The licence requires that the authorized person is satisfied that appropriate legal methods of resolving the problem such as scaring and proofing are either ineffective or impracticable.
• Shooting should only be used in a strategic manner as part of a coordinated programme designed to achieve sustained effective control.
• Shooting as a lethal method can be effective in reducing localised populations of birds when low numbers are involved. However, it is labour intensive, costly and rarely effective in achieving long-term reductions in bird numbers or associated damage. Other birds will often

move into an area to take the place of those that are killed. Also, some species of bird, particularly parrots, learn to avoid shooters.

• Shooting may be used as a scaring strategy to train birds to associate the sudden, sharp noise with real danger and subsequently, a fear of humans and human activities. Birds can be frightened away without attempts to kill them although small numbers of birds are usually killed with a view to enhance the scaring effect.

• Shooting may actually increase the damage levels in some crops, where birds may drop the seed head they are feeding on when scared off, and then attack a new one on their return.

• With widespread and common species damage control is best achieved by action targeted at problem areas.

• A trained gundog may be used to locate and recover dead or wounded birds as quickly as practicable. For further information on the use of gundogs refer to the BASC Code of Practice on this subject.

• Shooting of pest birds should only be performed by skilled operators who have the necessary experience with firearms and who hold the appropriate licences and accreditation. Storage and transportation of firearms and ammunition must comply with relevant legislation requirements.

• The optimum time for shooting will depend on the species of bird and the type of crop being protected. During the breeding season, corvids are territorial and so shooting may be less effective. At other times of the year, particularly during autumn/winter when food is less abundant, rooks, jackdaws and carrion crows may form large flocks; this may provide the best opportunity for shooting them. However, the efficacy of shooting in terms of reduced density or damage also needs consideration. Removing birds during or just prior to the breeding season may cause greater reductions in density in the long term or for the approaching season.

Animal Welfare Considerations

Impact on target animals

• Shooting is not a single, standard technique; there are many factors that can vary and influence the humaneness of the method. These include the type of weapon, calibre, choke, size and number of shot and load, range, ability of the shooter, movement and direction of the bird, exposure time, terrain and weather.

• Humaneness of shooting as a control technique depends almost entirely on the skill and judgement of the shooter. If properly carried out, it is one of the most humane methods of destroying pest birds. On the other hand, if inexpertly carried out, shooting can result in wounding which may cause considerable pain and suffering.

STANDARD OPERATING PROCEDURE

• Shooting must be conducted in a manner which maximises its effect thus causing rapid death. This requires the use of appropriate firearms and ammunition.

• Shooters should not shoot at a bird unless it is clearly visible and they are confident of killing it with a single shot.

• The shooter should aim to have the bird in the centre of the pattern at the point of impact.

• Only one bird should be targeted at a time. Shooting with a shotgun at a group of birds flying overhead often results in welfare problems as the birds aligned with the central cluster of pellets will usually be fatally injured, but those at the perimeter of the volley may only be hit be one or two pellets and stand a good chance of surviving. These birds are likely to experience suffering.

• Wounded birds must be located and killed as quickly and humanely as possible with either a second shot preferably directed to the head or in restrained or immobile birds, a blow to the rear of the skull to destroy the brain. If left, wounded birds can suffer from the disabling effects of the injury, from sickness due to infection of the wound, from pain created by the wound or from thirst or starvation if unable to drink or eat. Wing fractures, which increase the likelihood of being taken by a predator, are common in wounded birds.

• Any gundog used must be adequately controlled to prevent it from chasing or catching live birds that are not wounded. Dogs should only be trained to retrieve wounded or dead birds, under the direction of the handler, without causing physical injury to the bird.

• To minimise the animal welfare implications of leaving dependent nestlings and chicks to die from starvation it is preferable not to undertake shooting during the nesting season. If shooting must occur during nesting, reasonable efforts should be made to find nests containing young birds so they can be killed quickly and humanely.

Impact on non-target animals

• Shooting is relatively target specific and does not usually impact on other species. However, there is always a risk of injuring or killing non-target animals, including protected birds that have been mistaken for a pest bird. Only shoot at the target bird once it has been positively identified and never shoot over the top of hills or ridges as other animals or people may be out of sight beyond the hill in the danger zone.

• Shooting should be used with caution around lambing paddocks as it may disturb the lambing flock and cause mis-mothering. Also, avoid paddocks containing horses or deer. They are easily frightened by spotlights and gunshots and may injure themselves by running into fences and other obstacles.

Health and Safety Considerations

• It is an offence (except in certain circumstances) to possess, purchase or acquire a firearm or ammunition without an appropriate firearms certificate or temporary police permit.
• Firearms are potentially hazardous. Users should observe all relevant guidelines on ownership, possession and use. Users should follow BASC's Best Practice guidelines on shotgun use, e.g. when carrying, transporting, holding, passing, using, maintaining and storing a gun. Always handle a gun as if loaded, carry it empty, in a case or open and over the crook of the arm, keep the safety catch on until the shot is about to be taken and only touch the trigger when firing a shot. It is an offence to be in a public place with a loaded firearm under The Firearms Act 1968.
• Maintain guns in good condition.
• Ensure cartridges are compatible with the gun and of an appropriate type and shot size for shooting corvids. Do not mix cartridges of different bores.
• When not in use, firearms must be stored securely in a purpose-built firearms cabinet that meets UK legal requirements, to prevent access by unauthorised persons. Ammunition must be stored in a locked container in cool, dry conditions, separate from firearms.
• It is advisable to have adequate legal liability (third party) insurance when shooting.
• Take measures to ensure the safety of people not involved in shooting, including those using roads, footpaths, bridleways etc. Do not shoot across public rights of way. Take special care to warn riders using rights of way in earshot as horses may be easily frightened by loud noises, potentially threatening the safety of riders, horses, road users and others nearby.
• Never shoot at or near overhead power lines or insulators.
• When a bird is being shot, any other people should stand well behind the shooter. The line of fire must be chosen to prevent accidents or injury from stray bullets or ricochets.
• Shooting from a vehicle is potentially dangerous – never shoot when the vehicle is moving. An agreed safety procedure between the shooter and others in the vehicle must be in place to ensure that people do not enter the field of fire or disturb the taking of a shot.
• Only take safe shots, when there is no risk of injury to other people and when the target can be identified with certainty as a member of the relevant corvid species.
• Ear defenders should be worn by the shooter and others in the immediate vicinity of the shooter. Repeated exposure to firearm noise can cause irreversible hearing damage.
• Safety glasses or goggles are recommended to protect eyes from gases, metal fragments and other particles.

• Always carry a mobile phone, and where possible a six-figure grid reference of the area for emergency purposes.

• Warm, comfortable clothing and stout footwear to ensure a good grip and traction when taking up a firing position is recommended, especially when shooting at night.

• Care must be taken when handling birds (especially pest species) as they may carry diseases such as psittacosis (chlamydiosis), aspergillosis, erysipelas, yersiniosis and salmonellosis that can affect humans and other animals. Routinely wash hands after handling all birds. Personal protective equipment, especially face masks, are recommended when handling bird carcases to reduce the risk of contracting disease.

Equipment Required

Firearms and ammunition

• A twelve gauge shotgun and shot sizes between No. 4 and No. 6 are recommended for corvids at a distance of 30 metres.

• Non-toxic shot (e.g. tungsten-bismuth-tin, bismuth, tungsten-iron, steel, bismuth-tin, zinc etc) is preferable. Lead shot is potentially toxic to a range of species and it is illegal to use lead shot over wetland areas in Britain. Animals may be poisoned by lead in one of two general ways:

- Species such as waterfowl mistake spent shot for food or grit and ingest it from wetland or terrestrial environments.
- Other species, especially eagles and other raptors, and scavengers, ingest pellets when they consume prey that have been shot with shotgun ammunition and are carrying shot pellets embedded in their tissues.

• If intending to use steel shot ensure that it is safe and effective to do so in your gun. Steel pellets should only be discharged in modern guns that are capable of withstanding the extra stresses produced.

• Ensure that the choke configuration of a shotgun delivers a dense pattern on the target within the specified distances.

• The accuracy and precision of firearms should be tested against inanimate targets prior to the commencement of any shooting operation. Pattern your chosen gun/cartridge/choke combination before shooting to check your accuracy and that the pattern is adequate for the intended target bird.

Other equipment:

• First Aid kit.
• Gloves.
• Lockable firearm box.
• Lockable ammunition box.

Procedures

Assessing the need to shoot

• Corvids may only be shot under General Licence WML - GL04 if the authorized person is satisfied that appropriate legal methods of resolving the problem, such as scaring, are either ineffective or impracticable (see SOPs UKBIR002 and UKBIR004 for scaring birds using gas guns and visual devices respectively). Assess damage and the need for shooting by regular monitoring of the site during the time of year that crops are vulnerable. The species causing damage should be identified.
• Assess the suitability of the area for shooting before shooting begins. During daylight hours, shooters should identify suitable shooting points, firing directions and back-stops, and familiarise themselves with the terrain they are to cover, taking note of potential hazards and any landmarks that may help with navigation.
• Contact Natural England’s Wildlife Management Advisors for more information and advice on site assessment and monitoring of corvid numbers.

Identification of birds

• Shooters should have sufficient knowledge and skill to identify the bird species causing the damage. If the identification of the bird is in doubt it must not be shot.

Conduct of shooting

• Shooting should only be conducted during daylight hours. Shooting in poor light conditions makes it difficult to correctly identify birds and to search for wounded birds. Also, accurate marksmanship may be compromised.
• Shooting should not be conducted in adverse weather conditions where birds cannot be shot and located/retrieved in a safe and humane manner.
• Birds must NOT be shot from a moving vehicle or other moving platform. Ensure you are in a firm, safe and stable position before taking a shot.

Target bird and point of aim

• Only one bird should be targeted at a time. The shooter should aim to have a single bird in the centre of the shot pattern at the point of impact. Shooting at a flock is not an acceptable practice.

• The objective is to fire at the closest range practicable in order to reduce the risk of non-lethal wounding. Accuracy is important to achieve a humane death. One shot should ensure instantaneous loss of consciousness and rapid death without resumption of consciousness.

• A pest bird should only be shot at when:

- It can be clearly seen and identified;
- It is within the effective range of the firearm and ammunition being used; and
- A humane kill is highly probable. If in doubt, do NOT shoot.

• For corvids, the point of aim should be the centre of the birds' chest.

• When using a shotgun, the target bird may be stationary or mobile, but must be no more than 30 metres from the shooter. The pattern of shot should be centred on the chest for corvids. It is essential that the distance to the target bird is accurately judged. To achieve adequate penetration of shot, the bird must be in range. It is recommended that shooters practice estimating distances before a shooting operation.

• The target bird should be checked to ensure it is dead before moving on to the next bird. When targeting multiple birds in a flock, a number of birds will need to be shot in rapid succession. In this case, the birds in the group should be checked to ensure they are dead before moving on to the next group. A gundog may be used to retrieve wounded or dead birds. Death of shot birds should always be confirmed by observing the following:

- Absence of movement
- Absence of rhythmic, respiratory movements.
- Absence of heart beat – feel the chest between thumb and forefinger
- Absence of eye protection reflex (corneal reflex) or 'blink'.

• If death cannot be verified, a second shot to the head should be taken immediately or the bird killed with a blow to the skull using a heavy instrument to destroy the brain.

• Killed birds must be collected and disposed of lawfully.

Assessing effectiveness

• The effectiveness of a shooting operation in reducing bird numbers and damage should be monitored at intervals after treatment for comparison with figures before treatment.

Procedural Notes

• More detailed information on cage trapping corvids to prevent serious damage or disease can be found in the General Licence WML - GL04.

References

This SOP was adapted from BIR001 shooting of pest birds, prepared by Trudy Sharp (2012).

BASC (2009) *Gundogs; A Code of Practice*. http://www.basc.org.uk/en/codes-of-practice/gundogs.cfm.

BASC (2008) *Code of Good Shooting Practice*. http://www.basc.org.uk/en/codes-of-practice/code-of-good-shooting-practice.cfm.

BASC (2010) *Respect for Quarry; A Code of Practice*. http://www.basc.org.uk/en/codes-of-practice/respect-for-quarry.cfm.

BASC (2010) *Shotgun Code of Practice*. http://www.basc.org.uk/en/codes-of-practice/shotgun-code-of-practice.cfm.

Natural England (2012) *Licence (General): To kill or take certain wild birds to prevent serious damage or disease*. http://www.naturalengland.org.uk/Images/wlc-gl04_tcm6-24149.pdf.

Sharp T (2012) *BIR001 shooting of pest birds; standard operating procedure*. Invasive Animals Co-operative Research Centre, Australian Government. http://www.feral.org.au/wp-content/uploads/2013/03/BIR001_shooting-pest-birds.pdf
