# Supplementary material for: Assessing Animal Welfare Impacts in the Management of European Rabbits (Oryctolagus cuniculus), European Moles (Talpa europaea) and Carrion Crows (Corvus corone)
Source: PLoS One. 2016 Jan 4;11(1):e0146298. doi: 10.1371/journal.pone.0146298 (PMC4699632; doi:10.1371/journal.pone.0146298)
Supplement: S7 SOP — (PDF) [file pone.0146298.s007.pdf]

STANDARD OPERATING PROCEDURE

Cage trapping and cervical dislocation of corvids

Background

Corvids, such as rooks and crows, may cause agricultural or horticultural damage by feeding on cereals, fruit and other crops. Corvids may be cage-trapped, either in Larsen traps or multi-catch traps, for the purpose of preventing serious damage to crops, vegetables, fruit, foodstuffs for livestock and certain other enterprises, or for preventing the spread of disease. Alternative methods of corvid management include shooting, visual or auditory scarers, habitat manipulation, diversionary feeding and exclusion netting.

The aim of trapping is to reduce bird numbers in order to minimise damage. However trapping is often labour intensive, opportunistic and may have limited value in bird control. After trapping, pest birds are humanely killed. This Standard Operating Procedure (SOP) is a guide only; it does not replace or override the legislation and should only be used subject to the applicable legal requirements.

Application

• All wild birds, their eggs and nests are protected under The Wildlife and Countryside Act 1981. However, there is a General Licence under the Act (WML - GL04), which allows crows, rooks, jackdaws, magpies and jays to be killed or taken to prevent serious damage or disease. This may only be undertaken by an ‘authorised person’ under the licence. The licence requires that the authorised person is satisfied that appropriate legal methods of resolving the problem such as scaring and proofing are either ineffective or impracticable.
• The General Licence also allows the use of crows, rooks, jackdaws, magpies and jays as live decoys in Larsen traps, which are generally prohibited under the 1981 Act. However it is illegal to use sound recordings or tethered or disabled live decoys.
• The General Licence also allows crows, rooks, jackdaws, magpies and jays (and certain other species) to be caught in multi-catch traps.
• With widespread and common species damage control is best achieved by action targeted at problem areas.

- The optimum time for trapping will depend on the species of bird and the type of crop being protected. During the breeding season, corvids are territorial and so trapping may be less effective. At other times of the year, particularly during autumn/winter when food is less abundant, some species, e.g. rooks, jackdaws and carrion crows, may form large flocks and many birds can be caught. However, the efficacy of trapping in terms of reduced density or damage also needs consideration. Removing birds during or just prior to the breeding season may cause greater reductions in density in the long term or for the approaching ripening season.
- Confinement in a trap causes fear and distress; therefore traps need to be carefully managed.
- Operators should be competent in bird handling and restraint techniques. This will help to minimise harm to the birds and protect the handler from injury.
- Trapped pest birds should be euthanased after capture.
- It is illegal to sell decoy birds.

Animal Welfare Considerations

Impact on target animals

- Any bird caught in a trap becomes a Protected Animal under the Animal Welfare Act 2006. The person deemed responsible for a Protected Animal is obliged not to cause it unnecessary suffering which could reasonable have been avoided or reduced. An offence is committed whether through and act, or a failure to act, and it is also an offence not to provide for an animal’s needs, such as food, environment and protection from pain, suffering, injury and disease.
- When in use, traps need to be inspected at intervals of no more than 24 hours, except where this is not possible because of severe weather conditions, e.g. heavy snowfall. In such cases the trap should be inspected as soon as possible. Twice daily checks are recommended. Regular inspections will help to prevent captured birds from being harmed by other captured birds or by predators outside of the trap, e.g. foxes, badgers.
- At each inspection any target birds captured must be removed and killed quickly and humanely with minimal delay.
- Dead birds or other animals caught in the trap must also be removed at each inspection.
- Trapped birds are likely to suffer from distress when confined and they can sometimes be injured while trying to escape from the trap or during capture or restraint prior to euthanasia.

STANDARD OPERATING PROCEDURE

• Under the 1981 Act, traps must provide sufficient height, length, and breadth to permit a bird (decoy or target bird) to stretch its wings freely, but the General Licence allows cage traps which do not satisfy this requirement.

• If decoy birds are used, they must be provided with adequate food, water, shelter and a suitable perch, and they must be protected from pain, suffering, injury and disease. The decoy must be removed when the trap is not in use. Traps containing decoy birds must be inspected regularly i.e. for small traps at least once daily, for larger traps at least every two days. Decoy birds that show signs of prolonged distress (see Impact on non-target animals section) should be euthanased.

• Adequate protection from the elements is essential for the humane operation of the trap. Care should be taken in positioning the trap and shade and waterproofing can be incorporated as necessary. Where possible, trapping should be avoided in adverse weather conditions.

• Captured birds must be approached carefully and quietly to reduce panic, further stress and risk of injury.

• Special care and knowledge is necessary for holding or restraining birds, and the most appropriate method should be used for the species concerned.

• Trapped birds are euthanased using cervical dislocation. This involves separation of the skull and the brain from the spinal cord by pressure applied posterior to the base of the skull. The brain stem – which controls respiration and heart activity – is consequently damaged, stopping breathing and reducing blood flow to the brain, leading to death. Studies in rats have shown that electrical activity in the brain persists for around 13 seconds following cervical dislocation. This may represent a period of remaining consciousness.

• To minimise the animal welfare implications of leaving dependent nestlings and chicks to die from starvation it is preferable not to undertake trapping during the nesting season. If trapping must occur during nesting, reasonable efforts should be made to find nests containing young birds so they can be killed quickly and humanely.

• When a cage trap is left in the open but not in use, bait, food and water should be removed and the trap must be rendered incapable of holding or catching birds, e.g. with the door secured in open position.

Impact on non-target animals

• Traps are not target specific; therefore other species, usually birds, may be caught.

• To reduce the impact on non-target species, traps should be placed in areas that are frequented by the target species. Free-feeding can assist in identifying the likelihood of capturing non target species, and appropriate areas for capture.

• Using decoy birds may help to minimise non-target bird capture and improve trap success.

• Non-target birds (any species not included in the licence) caught in traps must be visually inspected for injuries and signs of illness or distress before release. Stressed birds will close their eyes and may also hunch-up their necks and maintain a stiff and unusual looking posture. A rapid heart rate, loss of feathers, change in body temperature, trembling or shaking may also be observed. Birds should be dealt with as follows:

- o Birds which are unharmed should be immediately released at the site of capture. If a bird has been handled, do not release it into mid-air. Turn it right side up and allow it to sit in the ground so that it can become oriented.
- o Birds which are suffering from thermal stress should receive appropriate attention. A bird suffering from thermal stress can initially be placed in a suitable quiet holding area which provides warmth or shade to allow recovery before release. Heat stressed birds will drink sugared water while they are being held in the hand.
- o Birds that are unable to fly may be suffering from a slight strain to the wings. Place them on a perch in good cover and they will usually recover rapidly.
- o Birds with treatable minor injuries that cannot be immediately released or those failing to recover from thermal stress should be presented to a vet or a registered wildlife carer for treatment.
- o Birds that have injuries which are untreatable or which would compromise their survival in the wild should be euthanased using cervical dislocation.

Health and Safety Considerations

• Care must be taken when handling birds (especially pest species) as they may carry diseases psittacosis (chlamydiosis), aspergillosis, erysipelas, yersiniosis and salmonellosis that can affect humans and other animals. Routinely wash hands after handling all birds. Personal protective equipment, especially face masks, are recommended when handling birds to reduce the risk of contracting disease.

• Operators need to be wary of the potential for injury when handling birds. Some species of birds can deliver painful bites and scratches. Protective gloves can be used if required for handling birds, although these may hinder dexterity. A towel is useful to place over the bird’s head.

• Operators must be protected by tetanus immunisation in case of infection of scratches and bites.

• During set-up of traps, operators should be wary of the risks of injury from lifting heavy items.

Equipment Required

Traps

• A Larsen cage trap has a closed compartment for confining a live decoy bird and a spring activated trap door which allows either top or side entry.

• Several types of multi-catch trap are available, the ladder/letterbox design being most popular for controlling crows, rooks and jackdaws. This is a large, permanent or moveable timber framed catcher with the top and sides covered in small wire mesh. Birds are lured in with food and a ladder type arrangement on top of the pen allows corvids to drop into the trap to feed but not to fly back out. A recommended minimum size would be 2m deep x 2m wide x 3m long and the trap should include a door to facilitate daily inspection.

Other equipment

• Bait.

• Handheld nets.

• Calico bird-bags.

• Gloves.

• First aid kit.

Procedures

Assessing the need to trap

Before deciding to set live traps to catch corvids it is important to establish the need to do so in order to prevent serious damage or disease, and that other appropriate legal methods of resolving the problem are either ineffective or impracticable.

Trapping of birds

• An ideal trap site is where the birds are already feeding, but traps can also be placed near roosts and along the route from the roosting area to the feeding ground.

• Traps may need to be tied down in the event of windy weather.

• A period of free-feeding using appropriate bait is recommended prior to the commencement of trapping, to both limit non-target captures and to improve trap success.

• Regular checking of traps ensures provision of clean food, water and shade. Larsen traps need to be checked at least every 24 hours. However, the frequency of trap monitoring will

depend on a number of factors including trap success, presence of predators, number of decoy birds, or if decoy birds are observed not to be eating, or appear unwell or stressed, e.g. through feather loss, lethargy etc. [In multi-capture traps the frequency of checking should increase when many birds are being captured.]

• Remain quiet when checking traps so as not to frighten birds that are in or near the trap.

• To reduce panic and injury to birds, always approach the traps slowly, particularly when there are birds inside. When free-feeding, ensure that birds inside the trap are able to leave it without panic.

• When removing non-target birds from the trap, always remove the larger birds first as their movements can also injure the smaller ones.

• Animals such as dogs and cats and non-essential personnel must be kept away from the area whilst the trap is in operation.

• Avoid setting traps on or near to public footpaths or public rights of way.

Euthanasia of trapped birds and disposal of carcases

Neck (cervical) dislocation:

- This technique requires mastering of technical skills to ensure that loss of consciousness is rapidly induced.
- Carefully remove birds from the trap by hand or using a hand held net.
- Dislocate the neck by taking the birds legs in the left hand (if right handed) and the head between the first two fingers of the right hand with the thumb under the beak. A sharp jerk with each hand, pulling the head backward over the neck will break the spinal cord and carotid arteries.
- Cervical dislocation is not suitable for birds larger than 3 kg as it is difficult to pull the neck quickly. Most pest birds will be below 3 kg in weight.

• Death of euthanased birds should always be confirmed by observing the following:

- Absence of movement
- Absence of rhythmic, respiratory movements.
- Absence of heart beat – feel the chest between thumb and forefinger
- Absence of eye protection reflex (corneal reflex) or ‘blink’.

• If death cannot be verified, a second method should immediately be used to kill the bird. Carcasses should only be discarded once death has been established.

• Bird carcasses should be collected and disposed of in an appropriate manner in accordance with acceptable practices as required by legislation and local authorities.

Euthanasia of nestlings and destruction of eggs

• The most suitable methods of euthanasia for chicks and nestlings are:

- o *Cervical dislocation* – effective and humane
- o *Decapitation* – the instrument used must be sharp and well maintained. In larger chicks the method should be performed after a blow to the head to render the bird unconscious.
- o *Concussion (stunning)* – a blow on the head will usually be sufficient to render the bird insensible. To ensure death stunning must be followed by another method e.g. decapitation or exsanguination (bleeding-out).

• It is believed that in avian embryos greater than half of the way to hatching, the neural tube has developed sufficiently to allow perception of pain. Therefore, it is preferable that eggs are destroyed by cooling or freezing them to < 4ºC for at least 4 hours. However, under field conditions quickly breaking the eggs and decapitation or crushing of the embryo may be a humane and more practical alternative.

Assessing effectiveness

• The effectiveness of a trapping operation should be monitored at intervals by assessing ongoing damage levels and comparing with levels before treatment.

Procedural Notes

• More detailed information on cage trapping corvids to prevent serious damage or disease can be found in the General Licence WML - GL04.

References

This SOP was adapted from BIR002 cage trapping of pest birds, prepared by Trudy Sharp (2012).

Natural England (2009) *A guide to cage-trapping birds in premises to preserve public health or public safety*. Natural England Technical Information Note TIN071. http://publications.naturalengland.org.uk/publication/33010?category=28001.

Natural England (2012) *Licence (General): To kill or take certain wild birds to prevent serious damage or disease*. http://www.naturalengland.org.uk/Images/wlc-gl04_tcm6-24149.pdf

British Association for Shooting and Conservation (????) *Trapping pest birds; a code of practice*. http://www.basc.org.uk/en/codes-of-practice/trapping-pest-birds.cfm.

RSPB website, http://www.rspb.org.uk/.

BTO website. http://blx1.bto.org/birdfacts/.

Sharp T (2012) *BIR002 trapping pest birds; standard operating procedure*. Invasive Animals Co-operative Research Centre, Australian Government. http://www.feral.org.au/wp-content/uploads/2013/03/BIR002_trapping-pest-birds.pdf
