# Supplementary material for: Assessing Animal Welfare Impacts in the Management of European Rabbits (Oryctolagus cuniculus), European Moles (Talpa europaea) and Carrion Crows (Corvus corone)
Source: PLoS One. 2016 Jan 4;11(1):e0146298. doi: 10.1371/journal.pone.0146298 (PMC4699632; doi:10.1371/journal.pone.0146298)
Supplement: S8 SOP — (PDF) [file pone.0146298.s008.pdf]

Scaring corvids using gas guns

Background

Corvids, such as rooks and crows, may cause agricultural or horticultural damage by feeding on cereals, fruit and other crops. A variety of auditory bird scaring measures may be used to deter birds from landing and feeding in vulnerable locations. These include gas guns, pyrotechnics, bio-acoustics (sounds of biological relevance such as recorded distress calls) and other acoustics (such as high frequency noise). Gas guns are mechanical devices that produce loud banging noises by igniting either acetylene or propane gas to imitate the sound of shotgun fire. In many situations lethal control, such as shooting or cage trapping followed by dispatch, may have little effect on damage, and using scarers when crops are vulnerable may be more effective. Some shooting may be used to reinforce and enhance scaring methods. Alternative methods of corvid management include habitat manipulation, chemical repellents, visual deterrents, diversionary feeding and exclusion. This Standard Operating Procedure (SOP) relates to the use of gas guns for deterring corvids from feeding on crops. It is a guide only and does not replace or override the legislation and should only be used subject to the applicable legal requirements.

Application

• All wild birds, their eggs and nests are protected under The Wildlife and Countryside Act 1981. While there is a General Licence under the Act (WML - GL04), which allows corvids to be killed or taken to prevent serious damage (or disease). However, lethal control is only permitted under a General Licence if appropriate non-lethal methods, such as scaring, are either ineffective or impracticable. Scarers likely to injure wild birds are prohibited.

• Limited information is available on the efficacy and best use of bird scaring devices. Auditory scaring techniques such as gas guns are generally considered relatively effective, but effectiveness varies with mode of deployment and the scaring effect is subject to habituation and hence of short-term benefit only. As with most non-lethal wildlife management techniques the effectiveness of gas guns will depend on the availability of alternative feeding areas close by.

• The gas gun ignites a mixture of gas and air under pressure, with the frequency of detonation regulated either by adjusting the gas feed or with an automatic timing device. The unexpected

bang produced causes a ‘startle’ reflex and promotes escape flight. Some guns can be controlled electronically, e.g. using battery power.

• Most gas guns produce a single report of up to 130dB(A), but double and multi-bang guns are also available; the human pain threshold is about 120dB. Other possible features are rotators that aim shots in different directions and electronic timers.

• Varying the position of gas guns and the pattern of sound emitted over time should prolong their effectiveness. Moving the cannon every few days is recommended along with variable firing intervals.

• Gas guns can cause noise nuisance and this must be taken into account in their use. Where it is practical to do so, you should seek to minimise the impact of auditory scarers on neighbours and consider alternative mean of scaring birds, e.g. visual deterrents. Care is required in deciding where to site gas guns, which direction to point them and whether or how to use sound baffles to deflect the sound. The proximity and type of neighbouring buildings, amenities and public rights of way need to be considered and where possible pest management should be coordinated with neighbouring landowners. The times at which firing begins and ends each day, and the pattern of firing, also need to be chosen sensitively.

• Use gas guns as infrequently as possible to minimise habituation and nuisance to other people. Determine when the crop is most vulnerable and plan to use scarers only then, but be prepared to deploy them at other times if a pest problem arises unexpectedly. Where possible take into account seasonal use of nearby amenities.

• Advice on firing frequency varies. It has been recommended that a low firing frequency should be used when bird numbers are low and that firing rates should be increased only if bird numbers increase because starting with a high frequency of firing is thought to encourage habituation. However, it has also been claimed that a shorter firing interval keeps birds vigilant, making them more easily dispersed.

• Where possible use as many different types of effective scarers as possible to maintain their novelty and reduce habituation; other auditory deterrents, visual deterrents and chemical repellents can all have a role to play.

• Combining or alternating the use of gas guns with other techniques, in an integrated control strategy, may be more effective than using them in isolation. While combinations have rarely been scientifically evaluated the general consensus was that they are most effective when used in combination with other scaring techniques. Gas guns that combine other stimuli are available. For example The ‘Rotating Hunter’ consists of two propane guns and a metal silhouette of a person that swivels with the force of each shot. The ‘Falcon Imitator’ has a gun that propels a fringed rubber disk up an eight-metre pole which then parachutes slowly

back down the pole, imitating a falcon chasing a bird.

• It may be possible to reduce the need for scarers and increase the effectiveness of those used by:

- Planting (where crop rotation allows) crops vulnerable to bird damage next to roads or other locations where the birds will be disturbed.
- Locating new areas of valuable crops as far away as possible from buildings where people sleep or where quiet is important, to minimise the impact of gas guns if it is necessary to use them.
- Growing small-scale crops under netting to exclude birds.

Animal Welfare Considerations

Impact on target animals

• Although corvids may be killed under General Licence to prevent damage, scarers likely to injure wild birds are prohibited.

• Gas guns produce extremely loud noise levels, which at close quarters would be considered damaging to human hearing. Unpleasantly loud noise no doubt combines with the startling effect of gas guns to deter birds from feeding on crops.

• Depending on the effect of a scarer, birds could be caused to waste time and energy by continuously approaching the protected field and then retreating when the gas gun fires. However, if birds learn quickly to avoid the protected site, this effect will be minimal.

• If alternative food is in short supply, an effective bird scarer could potentially restrict the food available to birds, or cause them to move on to another site further afield.

• Noise from gas guns could disturb roosting or nesting birds.

Impact on non-target animals

• Gas guns will potentially impact non-target bird species and other animals, by disturbing normal behaviour or encouraging animals to move elsewhere. Care should be taken not to place gas guns close to important areas for sensitive or protected species, e.g. bat roosts, the nests of endangered birds, badger setts etc.

• Horses are easily frightened and can bolt or unseat their riders. Scarers must not be positioned near roads or bridleways usually used by riders.

Health and Safety Considerations

• Gas guns produce loud noises which if experienced at close quarters may be well in excess of those considered acceptable at home or in the workplace. If used incorrectly gas guns have the potential to cause a range of health impacts as well as nuisance. Noise above safe levels may cause hearing damage, annoyance, stress, high blood pressure, sleep loss, the inability to concentrate, the inability to learn and loss of productivity. Gas guns therefore need to be positioned at an acceptable distance from public areas. As an example, the minimum distance at which gas guns can be placed from domestic dwellings in British Columbia, Canada, is 200m.
• The intensity of sound output from gas guns may be highly variable, both between guns and between explosions of an individual device. Noise intensity is also affected by local conditions, such as wind direction and strength; pointing cannons away from houses and constructing simple straw baffles around them allows them to be placed at approximately half the distance of guns without baffles, with no increase in noise nuisance.
• Maximise the distance between gas guns and roads, bridleways or other public rights of way. Point gas guns away from nearby roads or bridleways and where appropriate use baffles to absorb and redirect sound.
• It is important that gas guns are checked and maintained regularly to ensure safe function.
• Erect temporary signs to warn riders and other members of the public. Remove signs when guns are not in use.
• Provide neighbours with the name of a responsible contact in case the control on a gun fails. Display the name and telephone number at the nearest point of public access or inform the local Environmental Health Department where the scarer is located and give them contact details of the person responsible.
• During set-up and handling of gas guns, gas cylinders, ammunition and other associated equipment, operators should be wary of the risks of injury from firearms and from lifting heavy items.
• The Firearms Act 1968 requires a firearms certificate to be obtained, if bird scaring cartridges are used. Ammunition must be stored securely and used according to firearms legislation.
• The Environmental Protection Act 1990 includes powers to deal with nuisance from auditory bird scarers. These have been used successfully to stop offending farmers using such scarers.

Equipment Required

Gas gun

- Gas gun.
- Gas cylinder and attachments (or 12v lead acid battery/rechargeable battery where appropriate).
- Ammunition.

Baffles

- Straw bales or corrugated iron.
- Stakes for securing baffles.
- Spade.

Other equipment

- Pre-prepared, laminated warning signs.
- Ear defenders of the appropriate safety standard.
- First aid kit.

Procedures

Assessing the need for gas guns

- Monitor bird numbers at the site regularly during the time of year that crops are vulnerable to determine the need for scaring devices to protect crops.

Planning the use of gas guns

- Determine the need for using scaring devices to protect crops and the time of year that crops are vulnerable. Where practical consider scaring devices that are less likely to cause a nuisance than gas guns.
- Plan the deployment of gas guns carefully, in advance and, if relevant, in conjunction with neighbouring farmers. Use maps and site visits to check the best locations for siting guns, directions in which to point them, whether or where to use baffles and how to move guns around to maintain scaring effectiveness.

STANDARD OPERATING PROCEDURE

• Discuss plans for using gas guns with potentially sensitive neighbours, e.g. livestock holdings, hospitals, homes or schools and try to accommodate their needs where possible.

• Prepare warning signs in advance of deployment.

Deployment of gas guns

• Place guns as far apart as possible so that their combined effect does not cause a nuisance, taking account of the lie of the land, atmospheric conditions and plant cover. The area protected by a gas cannon can vary depending on the crop or habitat. Placing a cannon within a hide used by shooters and frequently moving it between such hides may prolong the scaring effects of both the shooting and the gas cannon.

• Position guns so that they are pointing downwind (where nuisance is not problem) - even a slight wind can affect the distance sound travels – and take particular care with devices that swivel with the wind.

• Prolong the effectiveness of guns by disguising or hiding them where possible – but not so that they could cause a risk of fire.

• Where practical, aim to minimise the impact of gas guns on neighbours. For example:
Place guns as far away from potentially sensitive neighbours as is practical, point them away from neighbours and use baffles.

• Programme the gas gun timer, interval length, volume and where the function is available the number of bangs at each firing. Test the gun using the test function where available.

• Never use gas guns before sunrise or after sunset. Avoid using them within at least 200m of sensitive buildings before 7.00am, or before 6.00am elsewhere, when sunrise is earlier. Use another method in the early morning and do not use after 10.00pm or later when sunset is later.

• Do not fire gas guns more than four times in an hour (bear in mind situations where several guns protect a single field). Birds can take considerably longer than 15 minutes to regroup. If the intervals are too short, birds will quickly habituate to the scarer. All the reports from a multiple discharge gun should count as one report if heard within 30 seconds.

• Where mechanical timers are used, ensure that they are reset regularly to take account of changing sunrise and sunset times. Where the operation of a gun is controlled by a photoelectric cell, ensure that this is kept clean and free from obstruction. Preferably, ensure that a mechanical timer backs up a photoelectric switch.

• Guns must be properly maintained and checked regularly for malfunctions that could cause complaints.

STANDARD OPERATING PROCEDURE

• Provide neighbours with a contact number in case the control on a gun fails. Display the name and telephone number at the nearest point of public access or inform the local Environmental Health Department where gas guns are located and provide them with contact details.

• Use reflective or absorbent baffles, e.g. corrugated iron or straw bales (2 bales high and 5 bales long in a wide, 90^0 U-shape around the back of the gun), to concentrate the sound on to your field and away from neighbours wherever nuisance could be caused.

• Avoid using gas guns on Sundays. Consider an alternative instead.

• Increase the effectiveness of gas guns by:

  - Reinforcing scaring with shooting, so that the noise is associated with real danger.
  - Using colour association or dummy gas guns to extend the effective area of the scaring operation. For example, place the gun inside a brightly-coloured container and deploy several similar, but empty containers in the field. Occasionally move the gun from one container to another.

• As soon as a gas gun loses effectiveness, replace it with another scarer of a different type, before it begins to signal a good feeding area.

Assessing effectiveness

• Monitor bird numbers and scaring success at regular intervals and adapt the scaring programme or introduce alternative scaring devices, or other control measures as necessary. Discontinue use of the gas gun if it becomes ineffective.

Procedural Notes

• Consult the National Farmers Union Code of Practice on using bird scarers: *Bird deterrents and bird scarers: protecting your crop*.

References

This SOP was adapted from BIR002 trapping of pest birds, prepared by Trudy Sharp (2012).

Ban the cannons (????) *Guidelines for the Use of Propane Cannons on Blueberry Farms in British Columbia*. http://www.banthecannons.com/guidelines.html

NFU (????) *Bird deterrents and bird scarers: protecting your crop – NFU Code of Practice.* http://www.birdscaring.co.uk/information/nfu-bird-scarers-code-of-practice_2006.htm

Bishop, J., McKay, H., Parrott D. & Allan J. (2003) *Review of international research literature regarding the effectiveness of auditory bird scaring techniques and potential alternatives*. *A report by the Central Science Laboratory to Defra*. http://archive.defra.gov.uk/environment/quality/noise/research/birdscaring/birdscaring.pdf, http://archive.defra.gov.uk/environment/quality/noise/research/birdscaring/references.pdf.

Nakamura, K. (1997) Estimation of Effective Area of Bird Scarers. *Journal of Wildlife Management*, **61**(3): 925-934.

Sharp T (2012) *BIR002 trapping pest birds; standard operating procedure*. Invasive Animals Co-operative Research Centre, Australian Government. http://www.feral.org.au/wp-content/uploads/2013/03/BIR002_trapping-pest-birds.pdf

Truecraft (????) *Bird scarer operating instructions*. http://www.spaldings.co.uk/Assets/PDFs/06218-OpInst-web10.pdf.
