# Supplementary material for: Assessing Animal Welfare Impacts in the Management of European Rabbits (Oryctolagus cuniculus), European Moles (Talpa europaea) and Carrion Crows (Corvus corone)
Source: PLoS One. 2016 Jan 4;11(1):e0146298. doi: 10.1371/journal.pone.0146298 (PMC4699632; doi:10.1371/journal.pone.0146298)
Supplement: S1 Table — From Sharp and Saunders (2011). (PDF) [file pone.0146298.s009.pdf]

DOMAIN 1: WATER DEPRIVATION, FOOD DEPRIVATION, MALNUTRITION

Impact category	Description of impact	Examples
NO IMPACT	No effect on food/water intake	
MILD IMPACT	Short-term water or food restrictions that are within usual tolerance levels for the species.	An animal has a few hours without water, in shade conditions.<br>Short-term deprivation of food.
MODERATE IMPACT	Water or food restrictions which cause serious short-term or moderate long-term effects on physiological state or body condition, but such effects remain within the capacity of the body to respond to nutritional variations and allow spontaneous recovery after restoration of a good quality diet.	An animal has a few hours without water, in hot, sunny conditions.<br>Deprivation of food long enough to bring about mobilisation of body fat stores.
SEVERE IMPACT	Severe restrictions on food/water intake that lead to significant levels of debility.	An animal has many hours without water.<br>Deprivation of food for many days resulting in severe loss of body weight.
EXTREME IMPACT	Extreme restrictions on food/water intake that would likely result in the animal dying from dehydration or starvation.	An animal has many days without water and /or food and dies from severe dehydration and/or starvation.
