# Supplementary material for: Assessing Animal Welfare Impacts in the Management of European Rabbits (Oryctolagus cuniculus), European Moles (Talpa europaea) and Carrion Crows (Corvus corone)
Source: PLoS One. 2016 Jan 4;11(1):e0146298. doi: 10.1371/journal.pone.0146298 (PMC4699632; doi:10.1371/journal.pone.0146298)
Supplement: S2 Table — From Sharp and Saunders (2011). (PDF) [file pone.0146298.s010.pdf]

DOMAIN 2: ENVIRONMENTAL CHALLENGE

Impact category	Description of impact	Examples
NO IMPACT	Exposure to environmental challenge is not a feature of or consequence of the mode of action.	Exposure to ambient conditions that are within an animals' thermoneutral range.
MILD IMPACT	Short term exposure to environmental conditions which are outside the normal range encountered by the animal but remain within their physiological adaptive capacity.	Exposure to levels of heat or cold which are outside the thermoneutral range, but which do not lead to debility in the long-term.
MODERATE IMPACT	Marked short-term or moderate long-term environmental challenges that elicit body responses beyond the physiological adaptive capacity of the animal, but where the untoward effects are readily reversed by restoration of normal ambient conditions.	Short-term heat stress caused by exposure to high ambient temperatures combined with exercise.
SEVERE IMPACT	Severe environmental challenges that lead to serious physiological compromise or permanent dysfunction, injury or illness.	An animal is exposed to severe heat or cold which could possibly lead to failure of thermoregulation and collapse.
EXTREME IMPACT	Long-term exposure to extremes of heat or cold that bring about the death of the animal from hyper- or hypothermia.	Animals that are left in leg-hold traps, cage traps or yards in extremes of heat or cold and subsequently die from hyper- or hypothermia.
