# Supplementary material for: Assessing Animal Welfare Impacts in the Management of European Rabbits (Oryctolagus cuniculus), European Moles (Talpa europaea) and Carrion Crows (Corvus corone)
Source: PLoS One. 2016 Jan 4;11(1):e0146298. doi: 10.1371/journal.pone.0146298 (PMC4699632; doi:10.1371/journal.pone.0146298)
Supplement: S3 Table — From Sharp and Saunders (2011). (PDF) [file pone.0146298.s011.pdf]

DOMAIN 3: INJURY, DISEASE, FUNCTIONAL IMPAIRMENT

Impact category	Description of impact	Examples
NO IMPACT	Disease, injury or functional impairment is not a feature of or consequence of the mode of action.	
MILD IMPACT	Body responses remain within the homeostatic capacity of the animal to react with no or only minor debility or incapacity.	Minor injuries (e.g. minor skin laceration, oedematous swelling of foot and/or leg, mild mouth injuries).<br>Minor functional impairment (e.g. mild vomiting/retching, diarrhoea).
MODERATE IMPACT	Disease/injury/functional impairment that results in moderately severe debility or incapacity but from which recovery would normally occur spontaneously.	Moderate injuries (e.g. damage to minor tendon or ligament, amputation of a digit, joint haemorrhage, single tooth fracture, major laceration of mouth or tongue, joint dislocation).<br>Moderate or functional impairment (e.g. moderate vomiting/retching, diarrhoea, increased breathing, moderate haemorrhages, convulsions).
SEVERE IMPACT	Injury/disease/functional impairment that result in severe debility or incapacity and serious physiological compromise and would normally cause permanent disability. Includes injuries that are likely to reduce survival if the animal were to be released.	Severe injuries (e.g. deep and wide lacerations, severed tendons, broken foot and leg bones below elbow or stifle, joint dislocations, amputations).<br>Severe or functional impairment (e.g. severe vomiting/retching, diarrhoea, abnormal breathing, severe haemorrhages, convulsions).
EXTREME IMPACT	Injury/disease/functional impairment that result in very severe debility or incapacity due to the effects of traumatic injury, infectious agent or toxin.	Extreme injuries (e.g. death caused by excessive blood loss or shock, spinal chord injury, severe internal bleeding, fractures of more than one limb, severe jaw fracture, fractures of limbs above elbow or stifle).<br>Extreme or functional impairment (e.g. extreme persistent vomiting/retching, diarrhoea, laboured breathing, convulsions, blindness, immobility/ prostration, excessive and prolonged haemorrhaging).
