# Supplementary material for: Assessing Animal Welfare Impacts in the Management of European Rabbits (Oryctolagus cuniculus), European Moles (Talpa europaea) and Carrion Crows (Corvus corone)
Source: PLoS One. 2016 Jan 4;11(1):e0146298. doi: 10.1371/journal.pone.0146298 (PMC4699632; doi:10.1371/journal.pone.0146298)
Supplement: S4 Table — From Sharp and Saunders (2011). (PDF) [file pone.0146298.s012.pdf]

DOMAIN 4: BEHAVIOURAL, INTERACTIVE RESTRICTION

Impact category	Description of impact	Examples
NO IMPACT	No interference with the behavioural needs of an animal (an animal's behavioural needs being those activities which when thwarted produce untoward physiological or psychological effects).	
MILD IMPACT	Mild interference with the behavioural needs of an animal.	Mild and short-term physical restraint resulting in minor behavioural or interactive restriction.
MODERATE IMPACT	Moderate interference with the behavioural needs of an animal resulting in negative physiological or psychological effects which are readily reversed after restoration of normal conditions.	Restraint that results in agitation from not being able to perform natural behaviour that the animal is highly motivated to perform e.g. feeding, moving, resting, grooming, mating, caring for young.
SEVERE IMPACT	Marked interference with the behavioural needs of an animal leading to physiological or psychological compromise that may cause long-term or permanent negative effects.	Severe abnormal self-directed behaviour e.g. chewing/biting of feet and limbs when restrained.<br>Normal defensive and/or escape reactions to visibility of or presence of predators are prevented.
EXTREME IMPACT	Extreme interference with the behavioural needs of individuals or groups of animals leading to psychotic-like behaviour or to agonistic interactions that result in very severe injury or death.	Restraint that results in extreme abnormal self-directed behaviour, excessive aggression, stereotypy (e.g. severe fighting among incompatible social groups, unfamiliar individuals that are in close proximity).<br>Inability to escape attack by a predator.
