# Supplementary material for: Assessing Animal Welfare Impacts in the Management of European Rabbits (Oryctolagus cuniculus), European Moles (Talpa europaea) and Carrion Crows (Corvus corone)
Source: PLoS One. 2016 Jan 4;11(1):e0146298. doi: 10.1371/journal.pone.0146298 (PMC4699632; doi:10.1371/journal.pone.0146298)
Supplement: S5 Table — From Sharp and Saunders (2011). (PDF) [file pone.0146298.s013.pdf]

DOMAIN 5: ANXIETY, FEAR, PAIN, DISTRESS, THIRST, HUNGER ETC.

Impact category	Description of impact	Examples
NO IMPACT	Anxiety, fear, pain, sickness, breathlessness, nausea, lethargy/ weakness. dizziness, greater than normal thirst and/or hunger or other negative affective experiences causing distress are not a feature or consequence of the method.	
MILD IMPACT	Mild anxiety, fear, pain, sickness, breathlessness, nausea, lethargy/ weakness. dizziness, unsatisfied thirst and/or hunger or other negative affective experience causing distress.	Limited human contact with no physical handling.
MODERATE IMPACT	Moderate anxiety, fear, pain, sickness, breathlessness, nausea, lethargy/ weakness. dizziness, unsatisfied thirst and/or hunger or other negative affective experience causing distress.	Moderate level of human contact with minimum of physical handling.
SEVERE IMPACT	Severe anxiety, fear, pain, sickness, breathlessness, nausea, lethargy/ weakness. dizziness, unsatisfied thirst and/or hunger or other negative affective experience causing distress.	High level of human contact with a degree of physical handling.
EXTREME IMPACT	Extreme inescapable or unrelieved anxiety, fear, pain, sickness, breathlessness, nausea, lethargy/ weakness. dizziness, unsatisfied thirst and/or hunger or other negative affective experience causing distress which is judged to be at or beyond the limits of reasonable endurance and results in the death of the animall.	Excitement, fear and distress in struggling restrained animals that result in death from capture myopathy.
