# Supplementary material for: Assessing Animal Welfare Impacts in the Management of European Rabbits (Oryctolagus cuniculus), European Moles (Talpa europaea) and Carrion Crows (Corvus corone)
Source: PLoS One. 2016 Jan 4;11(1):e0146298. doi: 10.1371/journal.pone.0146298 (PMC4699632; doi:10.1371/journal.pone.0146298)
Supplement: S6 Table — From Sharp and Saunders (2011). (PDF) [file pone.0146298.s014.pdf]

Overall impact on welfare	Duration of impact				
	Immediate to Seconds	Minutes	Hours	Days	Weeks
EXTREME	5	6	7	8	8
SEVERE	4	5	6	7	8
MODERATE	3	4	5	6	7
MILD	2	3	4	5	6
NO IMPACT	1	1	1	1	1
