# Supplementary material for: Assessing Animal Welfare Impacts in the Management of European Rabbits (Oryctolagus cuniculus), European Moles (Talpa europaea) and Carrion Crows (Corvus corone)
Source: PLoS One. 2016 Jan 4;11(1):e0146298. doi: 10.1371/journal.pone.0146298 (PMC4699632; doi:10.1371/journal.pone.0146298)
Supplement: S7 Table — From Sharp and Saunders (2011). (PDF) [file pone.0146298.s015.pdf]

Impact category	Description of impact	Examples
NO SUFFERING	No suffering before death. There is immediate death or immediate loss of consciousness lasting until death.<br><br>*Note that components of suffering include (but are not limited to) fear, anxiety, pain, distress, apprehension, sickness, fatigue, thirst, hunger.*<br><br>*Aversion refers to the avoidance or attempted avoidance of unpleasant, noxious stimuli and distressing stimuli*	Direct destruction/concussion of brain tissue resulting in rapid unconsciousness e.g. accurate shooting in the head.<br><br>Inhaled vapour with no irritant effect that induces unconsciousness without pain or discernable discomfort.<br><br>Does not involve physical handling or restraint
MILD SUFFERING	Loss of consciousness is not immediate and there is no or only minimal aversion and no or only mild suffering before death.	Inhaled vapour causing mild irritancy and mild pain and/or distress.<br><br>Mild dyspnoea (breathlesness).<br><br>Mild degree of sickness e.g. vomiting/retching, diarrhoea, lethargy/weakness etc.<br><br>Does not involve physical handling or restraint.
MODERATE SUFFERING	Loss of consciousness is not immediate and there is moderate aversion and suffering before death.	Inhaled vapour causing moderate irritancy and moderate pain and/or distress.<br><br>Moderate degree of sickness e.g. vomiting/retching, diarrhoea, lethargy/weakness etc.<br><br>Moderate dyspnoea.<br><br>May involve physical handling and restraint e.g. to administer an injectable agent via intravenous (IV) or intraperitoneal (IP) route of entry; to apply cervical dislocation; to apply blunt trauma to the head.

Impact category	Description of impact	Examples
SEVERE SUFFERING	Loss of consciousness is not immediate and there is severe suffering before death.	Inhaled vapour causing severe irritancy and severe pain and/or distress.<br><br>Convulsions occurring during unconsciousness when animal recovers consciousness prior to death (i.e. muscle spasms with periods of relaxation as in clonic convulsions).<br><br>Severance of major arteries resulting in rapid blood loss, hypovolaemia and shock.<br><br>Severe degree of sickness e.g. vomiting/retching, diarrhoea, lethargy/weakness etc.<br><br>Severe dyspnoea<br><br>May involve physical handling and restraint e.g. administration of an injectable agent to a non-sedated animal via a difficult-to-access route of entry (e.g. intracardiac, intrahepatic, intrarenal).
EXTREME SUFFERING	Loss of consciousness is not immediate and there is extreme suffering before death.	Inhaled vapour causing extreme irritancy and extreme pain and/or distress.<br><br>Partial or full paralysis whilst conscious.<br><br>Convulsions whilst conscious (i.e. prolonged muscle spasm without periods of relaxation as in tonic convulsions).<br><br>Extreme degree of sickness e.g. vomiting/retching, diarrhoea, lethargy/weakness etc.<br><br>Extreme dyspnoea.<br><br>Severe internal haemorrhages causing swelling within confined spaces.<br><br>May involve physical handling and restraint.
