# Supplementary material for: Assessing Animal Welfare Impacts in the Management of European Rabbits (Oryctolagus cuniculus), European Moles (Talpa europaea) and Carrion Crows (Corvus corone)
Source: PLoS One. 2016 Jan 4;11(1):e0146298. doi: 10.1371/journal.pone.0146298 (PMC4699632; doi:10.1371/journal.pone.0146298)
Supplement: S8 Table — From Sharp and Saunders (2011). (PDF) [file pone.0146298.s016.pdf]

Level of suffering (after application of the method that causes death but before insensibility)	Time to insensibility (minus any lag time)				
	Immediate to Seconds	Minutes	Hours	Days	Weeks
EXTREME	E	F	G	H	H
SEVERE	D	E	F	G	H
MODERATE	C	D	E	F	G
MILD	B	C	D	E	F
NO IMPACT	A	A	A	A	A
