# Supplementary material for: Assessing Animal Welfare Impacts in the Management of European Rabbits (Oryctolagus cuniculus), European Moles (Talpa europaea) and Carrion Crows (Corvus corone)
Source: PLoS One. 2016 Jan 4;11(1):e0146298. doi: 10.1371/journal.pone.0146298 (PMC4699632; doi:10.1371/journal.pone.0146298)
Supplement: S10 Table — (PDF) [file pone.0146298.s018.pdf]

Control method:	**Shooting rabbits - chest shot**

Assumptions	Best practice is followed in accordance with the Standard Operating Procedure S1.<br><br>Shooter uses appropriate gun and ammunition, is competent, and judges shot placement and range accurately.<br><br>Head shots are preferred but chest shots more likely at greater distances.<br><br>Wounding rates should be low if Standard Operating Procedure is followed (but rabbits are often shot by non-professionals).<br><br>Shooting is not recommended as a primary rabbit control technique, but may be useful as part of wider management effort.<br><br>Rabbits are often targeted when feeding in a group.<br><br>The impacts in Part A of the assessment were considered for a number of rabbits feeding together. The first rabbit would be naïve but the impact would probably increase for each successive rabbit shot in a particular shooting bout.<br><br>Rabbits may breed year-round but the impact of shooting on dependent kittens is not assessed.

PART A: assessment of overall welfare impact

DOMAIN 1 Water or food restriction, malnutrition				
No impact	Mild impact	Moderate impact	Severe impact	Extreme impact

DOMAIN 2 Environmental challenge				
No impact	Mild impact	Moderate impact	Severe impact	Extreme impact

DOMAIN 3 Disease, injury, functional impairment				
No impact	Mild impact	Moderate impact	Severe impact	Extreme impact

DOMAIN 4 Behavioural or interactive restriction				
No impact	Mild impact	Moderate impact	Severe impact	Extreme impact

DOMAIN 5 Anxiety, fear, pain, distress, thirst, hunger				
No impact	Mild impact	Moderate impact	Severe impact	Extreme impact

Overall impact
Mild impact

DURATION OF IMPACT				
Immediate to seconds	Minutes	Hours	Days	Weeks

SCORE FOR PART A:	**2-3**
Summary of evidence	
Domain 1	No impact in this domain.
Domain 2	No impact in this domain.
Domain 3	No impact in this domain.
Domain 4	Rabbits form stable and lasting social groups (Marsh, 2009, cited in Sharp & Saunders, 2009), and are usually shot at in a group. If several rabbits from the same group are shot in a single shooting bout then by the time a particular rabbit is shot it is likely to have experienced the shooting of other members of its group, experiencing disturbance as a result.
Domain 5	Rabbits may suffer briefly before being shot if another nearby rabbit is shot first, as a result experiencing fear/panic from the noise, the general disturbance, alarmed animals escaping. In such cases, remaining rabbits are most likely to try to go to ground, either avoiding being shot, being shot before they reach cover, or going to ground and then resurfacing to be shot minutes later during the same shooting bout. In the meantime, rabbits will exhibit natural 'flight or fight' stress response as when encountering a predator. These endocrine responses are short-term and stress hormone levels would quickly return to normal if the rabbit should escape being shot (Munck et al., 1984).

PART B: assessment of mode of death -	**Shooting rabbits - chest shot**

Time to insensibility (minus any lag time)				
Immediate to seconds	Minutes	Hours	Days	Weeks
Level of suffering (after application of the method that causes death but before insensibility)				
No suffering	Mild suffering	Moderate suffering	Severe suffering	Extreme suffering

SCORE FOR PART B:	**B**
Summary of evidence	
Duration	A chest shot is likely to cause insensibility within between seconds and a few minutes, but probably in the order of seconds in most cases. Time to loss of consciousness and time to death will be determined by which tissues are damaged and particularly the rate of blood loss, and therefore the rate at which cerebral hypoxaemia is induced (Gregory, 2005). A shot to the heart is likely to cause loss of consciousness and death rapidly. With chest shots, 'hydrostatic shock' (see comments below) may also be a factor in rapid incapacitation and potentially rapid loss of consciousness; this effect is unpredictable and does not always take place.
Suffering	An animal sustaining a chest shot and remaining conscious will probably suffer for a short time, but the degree of suffering will be determined by which tissues are damaged and the rate of blood loss. However, damage to the lungs, heart etc, is likely to be relatively extensive in a chest-shot rabbit, so suffering is likely to be mild. Haemorrhage is likely to be accompanied by tachypnoea (rapid respiration) and hyperventilation; if these are severe the animal may experience breathlessness before consciousness is lost (Gregory, 2005). Evidence from humans indicates that haemorrhage may also be associated with anxiety and confusion (Burgess, 1995). If hydrostatic shock renders an animal irreversibly insensible before death it is unlikely to suffer.

Summary

CONTROL METHOD	**Shooting rabbits - chest shot**
OVERALL HUMANENESS SCORE	**2-3B**
Comments	Wounding rates - this assessment assumed that rabbits were shot according to best practice, i.e. with the appropriate weapon and ammunition, from a suitable distance, and the shot accurately placed. However, studies with foxes have suggested that a proportion of the foxes shot at are wounded. One study, based on x-ray evidence of shooting wounds among animals admitted to wildlife hospitals and vets, estimated wounding rates to be 9% with shotguns and 3% with rifles (Bentley et al. unpublished data in Baker et al., 2006). Another study examined the accuracy of shooting, by shooters of varying skill levels, at life-size paper fox targets, and estimated that wounding rates could be considerably greater (Fox et al., 2005).<br><br>Hydrostatic shock - when an animal is shot its organs can be damaged both by the projectile and by the pressure wave or hydrostatic shock produced when the projectile enters the body (Courtney & Courtney, 2008). In some cases the pressure wave produced may be of sufficient pressure to kill an animal before it dies of blood loss effects (Courtenay & Courtenay, 2007).

Bibliography

Baker, P., Harris, S. and White, P. (2006). *After the hunt. The future for foxes in Britain* . International Fund for Animal Welfare (IFAW), London, UK. http://www.thefoxwebsite.org/After-the-Hunt.pdf.

Burgess, F.W., Sborov, M.J. and Calcagni, D.R. (1995). Hemorrhage, shock, and fluid resuscitation. In R. Zajtchuk, eds. *Anesthesia and Perioperative Care of the Combat Casualty.* Office of The Surgeon General at TMM Publications, Borden Institute, Washington DC, USA.

Courtney, A. and Courtney, M. (2007). Links between traumatic brain injury and ballistic pressure waves originating in the thoracic cavity and extremities. *Brain Injury,* 21: 657-662.

Courtney, M. and Courtney, A. (2008). *Scientific Evidence for Hydrostatic Shock* . 0803.3051 at <http://arxiv.org/abs/0803.3051>

Fox, N.C., Blay, N., Greenwood, A.G., Wise, D. and Potapov, E. (2005). Wounding rates in shooting foxes (*Vulpes vulpes*). *Animal Welfare* , 14: 93-102

Gregory, N.G. (2005). Bowhunting deer. *Animal Welfare,* 14: 111-116.

Munck, A., Guyre, P. and Holbrook, N. (1984). Physiological Functions of Glucocorticoids in Stress and Their Relation to Pharmacological Actions. *Endocrine Reviews* , 5: 25-44.

Sharp, T. and Saunders, G. (2009) *Ground shooting of rabbits; welfare assessment* . http://www.feral.org.au/wp-content/uploads/2012/02/rabbit_ground_shooting.pdf.
