# Supplementary material for: Assessing Animal Welfare Impacts in the Management of European Rabbits (Oryctolagus cuniculus), European Moles (Talpa europaea) and Carrion Crows (Corvus corone)
Source: PLoS One. 2016 Jan 4;11(1):e0146298. doi: 10.1371/journal.pone.0146298 (PMC4699632; doi:10.1371/journal.pone.0146298)
Supplement: S11 Table — (PDF) [file pone.0146298.s019.pdf]

**Control method:**	**Fencing against crop incursion by rabbits: a) fence installation**

Assumptions	Best practice is followed in accordance with the standard operating procedure S2.<br><br>This SOP is for two months starting just before installation of permanent (non-electric) wire-mesh fencing around the perimeter of a wheat field, this taking place after harvest and before the winter wheat crop is planted in September-October, to exclude from the newly planted wheat rabbits harboured in adjacent woodland.<br><br>Rabbits may breed year-round but the impact of fencing on dependent kittens is not assessed.

PART A: assessment of overall welfare impact

DOMAIN 1 Water or food restriction, malnutrition				
No impact	**Mild impact**	Moderate impact	Severe impact	Extreme impact

DOMAIN 2 Environmental challenge				
**No impact**	Mild impact	Moderate impact	Severe impact	Extreme impact

DOMAIN 3 Disease, injury, functional impairment				
**No impact**	Mild impact	Moderate impact	Severe impact	Extreme impact

DOMAIN 4 Behavioural or interactive restriction				
No impact	**Mild impact**	Moderate impact	Severe impact	Extreme impact

DOMAIN 5 Anxiety, fear, pain, distress, thirst, hunger				
No impact	**Mild impact**	Moderate impact	Severe impact	Extreme impact

Overall impact
**Mild impact**

DURATION OF IMPACT				
Immediate to seconds	Minutes	Hours	**Days**	Weeks

SCORE FOR PART A:	**5**
Summary of evidence	
Domain 1	Because the fencing is installed before winter wheat is planted, when the field is bare, there is no direct impact of putting up the fence on food availability in the field itself. Generally, rabbits graze close to their burrow, grazing less with increasing distance from the burrow (e.g. Bakker et al., 2005). So, while the new fence is unlikely to prevent rabbits from accessing forage in the field-edge on the opposite side of the field, it may prevent them from taking a short-cut across the field corner to access forage in the adjacent field edge. The increased distance to this forage caused by the new fence is unlikely to have more than a mild effect on food intake for a few days, during which time rabbits are likely to modify their ranging behaviour accordingly (McKillop & Wilson, 1999). Rabbits may be deterred from leaving their burrows to feed while the fence is being installed.
Domain 2	No impact in this domain.
Domain 3	No impact in this domain.
Domain 4	For a while after the fence is installed, the presence of the fence close to burrows may create mild behavioural restriction. However it has been observed that when new wire mesh fences are installed to protect crops from rabbits, rabbit numbers living close to protected areas did not change over six years (McKillop et al., 1998). However, rabbits are likely to alter their ranging behaviour within a few days as has been seen when new electric fencing is installed (McKillop & Wilson, 1999).
Domain 5	The appearance of the fence and temporary presence of people putting up the fence will cause a mild impact in this domain.

PART B: assessment of mode of death -
**Not performed - non-lethal method**

Summary

CONTROL METHOD	**Fencing against crop incursion by rabbits: a) fence installation**	
OVERALL HUMANENESS SCORE		**5**
Comments	Rabbits may breed year round, but installation of a fence around an uncropped field while rabbits have dependent young is unlikely to affect the offspring adversely.	

Bibliography

Bakker, E.S., Reiffers, R.C., Olff, H. and Gleichman, J.M. (2005) Experimental manipulation of predation risk and food quality: effect on grazing behaviour in a central-place foraging herbivore. *Oecologia* , 146 (1): 157-167.

McKillop, I.G., Butt, P., Lill, J., Pepper, H.W. and Wilson, C.J. (1998) Long-term cost effectiveness of fences to manage European wild rabbits. *Crop Protection* , 17 (5): 343-400.

McKillop, I.G. and Wilson, C.J. (1999) The behaviour of free-living European rabbits at electric fences. *Crop Protection* , 18: 193-197
