# Supplementary material for: Assessing Animal Welfare Impacts in the Management of European Rabbits (Oryctolagus cuniculus), European Moles (Talpa europaea) and Carrion Crows (Corvus corone)
Source: PLoS One. 2016 Jan 4;11(1):e0146298. doi: 10.1371/journal.pone.0146298 (PMC4699632; doi:10.1371/journal.pone.0146298)
Supplement: S12 Table — (PDF) [file pone.0146298.s020.pdf]

**Control method:**	**Fencing against crop incursion by rabbits: b) fence established**

Assumptions	Best practice is followed in accordance with the standard operating procedure S2.<br><br>This SOP is for two months in spring once a permanent (non-electric) wire-mesh fence, installed around the perimeter of a wheat field the previous autumn (to exclude rabbits harboured in adjacent woodland), has become established.<br><br>Rabbits may breed year-round but the impact of fencing on dependent kittens is not assessed.

PART A: assessment of overall welfare impact

DOMAIN 1 Water or food restriction, malnutrition				
No impact	Mild impact	Moderate impact	Severe impact	Extreme impact

DOMAIN 2 Environmental challenge				
No impact	Mild impact	Moderate impact	Severe impact	Extreme impact

DOMAIN 3 Disease, injury, functional impairment				
No impact	Mild impact	Moderate impact	Severe impact	Extreme impact

DOMAIN 4 Behavioural or interactive restriction				
No impact	Mild impact	Moderate impact	Severe impact	Extreme impact

DOMAIN 5 Anxiety, fear, pain, distress, thirst, hunger				
No impact	Mild impact	Moderate impact	Severe impact	Extreme impact

Overall impact
No impact

SCORE FOR PART A	1
Summary of evidence<br>Domain 1	By this time rabbits will have adjusted to the presence of the fence and any effects particularly on food availability. If rabbits are breeding, the number of offspring produced is likely to be influenced by existing food availability (with the fence in situ), because rabbits require green pasture with a high protein content for reproduction (Parer, 1987). Rabbit litter sizes are influenced by maternal diet, specifically a phenol found in sprouted wheat, 6-methoxybenzoxazolinone (6-MBOA), that increases litter size in rabbits and stimulates reproduction in other small wild herbivorous mammals (Rodriguez-De Lara et al., 2007). Therefore reproductive investment should be determined by the available maternal diet, and pregnant or lactating females should not suffer additional food stress as a result of the fence.
Domain 2	No impact in this domain.
Domain 3	No impact in this domain.
Domain 4	No impact in this domain.
Domain 5	No impact in this domain.

PART B: assessment of mode of death -
**Not performed - non-lethal method**

Summary

CONTROL METHOD	**Fencing against crop incursion by rabbits: b) fence established**	
OVERALL HUMANENESS SCORE		1
Comments	Rabbits may breed year round, but presence of a fence around an uncropped field while rabbits have dependent young is unlikely to affect the offspring adversely.<br><br>Assessment of established fence has been assessed over two months for comparison with assessment for two months starting with installation of the fence. However, the fence should have a similar impact throughout the growing season once established.	

Bibliography

Parer, I. (1987) Factors influencing the distribution and abundance of rabbits, *Oryctolagus cuniculus* , in Queensland, Australia. *Proceedings of the Royal Society of Queensland* , 98: 73-82.

Rodriguez-De Lara, R., Herrera-Corredor, C.A., Fallas-Lopez, M., Rangel-Santos, R., Mariscal-Aguayo, V,. Martınez-Hernandez, P.A. and Garcıa-Muniz, J.G. (2007) Influence of supplemental dietary sprouted wheat on reproduction in artificially inseminated doe rabbits. *Animal Reproduction Science* , 99: 145–155.
