# Supplementary material for: Assessing Animal Welfare Impacts in the Management of European Rabbits (Oryctolagus cuniculus), European Moles (Talpa europaea) and Carrion Crows (Corvus corone)
Source: PLoS One. 2016 Jan 4;11(1):e0146298. doi: 10.1371/journal.pone.0146298 (PMC4699632; doi:10.1371/journal.pone.0146298)
Supplement: S13 Table — (PDF) [file pone.0146298.s021.pdf]

**Control method:**	**Managing molehills on lawns**

Assumptions	Best practice is followed in accordance with the Standard Operating Procedure S5.<br><br>Molehill soil is carefully lifted using a shovel or spade, and either redistributed thinly over a wider area of grass, or used elsewhere. Where surface tunnels are present and cannot be tolerated, these are gently trodden down.

PART A: assessment of overall welfare impact

DOMAIN 1 Water or food restriction, malnutrition				
No impact	Mild impact	Moderate impact	Severe impact	Extreme impact

DOMAIN 2 Environmental challenge				
No impact	Mild impact	Moderate impact	Severe impact	Extreme impact

DOMAIN 3 Disease, injury, functional impairment				
No impact	Mild impact	Moderate impact	Severe impact	Extreme impact

DOMAIN 4 Behavioural or interactive restriction				
No impact	Mild impact	Moderate impact	Severe impact	Extreme impact

DOMAIN 5 Anxiety, fear, pain, distress, thirst, hunger				
No impact	Mild impact	Moderate impact	Severe impact	Extreme impact

Overall impact
No impact

SCORE FOR PART A:	**1**
Summary of evidence	
Domain 1	Moles are insectivores with a high metabolic rate and are highly dependent on a regular food supply. Their diet largely consists of earthworms, *Lumbricus terrestris* , from which they are likely to obtain much of their water. Moles excavate a system of underground tunnels in which to locate their prey (Gorman & Stone, 1990). Careful removal of molehill soil is unlikely to damage an existing tunnel system and in general moles are unlikely to suffer food or water shortages as a result.<br><br>Tunnels that breach the soil surface are relatively uncommon and short-lived structures and so these are relatively unlikely to be present (Gorman & Stone, 1990). Further, mole control is most frequently conducted on domestic lawns in summer, and if the weather is particularly dry and hot moles will be less likely to make surface tunnels as their prey migrate deeper into the soil and moles are forced to hunt them in their deeper tunnels (Gorman & Stone, 1990; Nicholls, 2010). If surface tunnels are present and need to be trodden down, the damage caused to the tunnel system will be restricted to these.
Domain 2	No impact in this domain.
Domain 3	No impact in this domain. Moles have a highly sophisticated sense of touch. Their short tails are covered with sensory hairs called vibrissae. As a mole travels through its tunnel system, its tail (which is held semi-erect) brushes against the tunnel walls and roof, picking up information including vibrations passing through the soil (Gorman & Stone, 1990). In this way, a mole is likely to detect the approaching activity, on the surface of the ground, and might retreat briefly to deeper parts of the tunnel system as a result; physical injury is unlikely to occur.
Domain 4	Moles might briefly avoid parts of their tunnel system that are near the ground surface. Overall this is unlikely to prevent them from performing behaviour that they are highly motivated to perform.
Domain 5	Since no tunnel system repairs are likely to be required, moles are unlikely to suffer any impact under this domain.

PART B: assessment of mode of death -
**Not performed - non-lethal method**

Summary

CONTROL METHOD	**Managing molehills on lawns**
OVERALL HUMANENESS SCORE	**1**
Comments	Careful removal of molehill soil should have no impact on mole welfare. Surface tunnels occur relatively rarely and can be trodden down carefully if they cannot be tolerated. No data are available on the likely impact of doing this on mole welfare or the proportion of moles likely to be affected. Based on knowledge of moles' sensory acuity, and ability to detect activity on the soil surface, it is predicted that they are unlikely to be killed or injured. Given the relatively rare occurrence of surface tunnels, no impact is predicted under all domains.

Bibliography

Gorman, M. L. and Stone, R.D. (1990). *The Natural History of Moles* . London, Helm.
Nicholls, J. (2010) *Mole catching; a practical guide* . Crowood Press, Marlborough, UK.
