# Supplementary material for: Assessing Animal Welfare Impacts in the Management of European Rabbits (Oryctolagus cuniculus), European Moles (Talpa europaea) and Carrion Crows (Corvus corone)
Source: PLoS One. 2016 Jan 4;11(1):e0146298. doi: 10.1371/journal.pone.0146298 (PMC4699632; doi:10.1371/journal.pone.0146298)
Supplement: S14 Table — (PDF) [file pone.0146298.s022.pdf]

Control method:	**Spring trapping moles**

Assumptions	Best practice is followed in accordance with the Standard Operating Procedure S3.<br><br>Traps used are Scissor, Duffus or Talpa traps that are assumed to meet the current requirements of the Spring Traps Approval process (80% of 12 animals are irreversibly unconscious within 5 minutes), although mole traps do not currently require approval (Baker et al. 2012).<br><br>Trapping is conducted in spring, but not during breeding period.<br><br>Traps are checked every 24 hours.

PART A: assessment of overall welfare impact

DOMAIN 1 Water or food restriction, malnutrition				
No impact	Mild impact	Moderate impact	Severe impact	Extreme impact

DOMAIN 2 Environmental challenge				
No impact	Mild impact	Moderate impact	Severe impact	Extreme impact

DOMAIN 3 Disease, injury, functional impairment				
No impact	Mild impact	Moderate impact	Severe impact	Extreme impact

DOMAIN 4 Behavioural or interactive restriction				
No impact	Mild impact	Moderate impact	Severe impact	Extreme impact

DOMAIN 5 Anxiety, fear, pain, distress, thirst, hunger				
No impact	Mild impact	Moderate impact	Severe impact	Extreme impact

Overall impact
No impact

SCORE FOR PART A:	**1**
Summary of evidence	Note that Part A of the assessment examines the 'impact on the animal prior to the action that causes death'. Part B then looks at the 'actual mode of death' and the 'extent and duration of suffering caused'. With lethal trapping there is usually no impact in Part A.
Domain 1	No impact in this domain.
Domain 2	No impact in this domain.
Domain 3	No impact in this domain.
Domain 4	No impact in this domain.
Domain 5	No impact in this domain.

PART B: assessment of mode of death -	**Spring trapping moles**

Time to insensibility (minus any lag time)				
Immediate to seconds	Minutes	Hours	Days	Weeks
Level of suffering (after application of the method that causes death but before insensibility)				
No suffering	Mild suffering	Moderate suffering	Severe suffering	Extreme suffering

SCORE FOR PART B:	**E**
Summary of evidence	
Duration	Mole spring traps are designed to catch moles around the body when a trigger plate or wire is pushed, releasing the killing mechanism, which kills the mole by crushing (Gorman & Stone, 1990). Mole spring traps are assumed to cause irreversible unconsciousness within five minutes, as per the current Spring Traps Approval requirements which are based on the Agreement on International Humane Trapping Standards (AIHTS, 1998). Moles are sometimes caught by extremities (Atkinson et al., 1994) or are found alive in spring traps (Baker et al., 2015), but since traps tend to be checked only once every 24 hours, it is not possible to say how long they have been trapped before being found.
Suffering	There are three ways in which spring traps can kill the target animal (Parrott et al., 2009). Ideally a spring trap will strike the mole in the correct anatomical location, and with sufficient force, to cause cranial or upper vertebra destruction. (The impact momentum generated by a mole trap will also cause physical damage to the nervous system, blood vessels and organs.) Alternatively, the clamping force of the trap may cause death in one of two ways. If the striking bar is located across the neck it can cause occlusion of blood vessels supplying the brain. If the bar is across the body, thoracic compression can cause hypoxia as a result of asphyxiation (Fera, 2009).<br><br>The small amount of evidence available suggests that spring trapped moles tend not to suffer broken vertebrae (Atkinson et al., 1994; Baker et al., 2015) and that most die largely of acute haemorrhage when major blood vessels are ruptured (most likely leading to acute haemorrhagic shock), while some may asphyxiate (Baker et al., 2015).<br><br>An animal dying from blood loss is likely to become unconscious before death and both time to unconsciousness and time to death depend on the rate of blood loss (Baker et al., 2015). Acute haemorrhagic shock is a severe clinical condition in which a sharp drop in blood pressure occurs following traumatic and haemorrhagic bleeding. Because of reduced oxygen supply, ischaemia (local anaemia) may occur in vital organs and irreversible change or death may result (Xu et al., 2011). The key systems affected by haemorrhagic shock are the central nervous, cardiac, and renal systems (Falk et al., 1992). Symptoms will depend on the volume and rate of blood loss (Smith, 1997). Severe haemorrhagic shock involves rapid blood loss, increased heart rate, a profound drop in blood pressure, delayed capillary refill, tachypnea (rapid breathing) and respiratory collapse, anuria (no urine output) and the patient is lethargic and obtunded (dulled reflexes) (Martel et al., 2002).

Summary

CONTROL METHOD	**Spring trapping moles**
OVERALL HUMANENESS SCORE	**1E**
Comments	Spring trapping of moles may have a greater welfare impact than that identified here, because mole traps are not currently subject to the British Spring Traps Approval process (Baker et al., 2012).<br><br>Moles may sometimes detect a trap that has been set in their run and block it with soil before tunnelling around it (Nicholls, 2010). This is likely to affect only a minority of trap-settings and to have no more impact on moles than when moles themselves conduct routine maintenance to damaged tunnels.<br><br>The degree of suffering in the trap will depend on the rate of blood loss which determines whether the animal becomes unconscious before death.<br><br>New trap standards proposed by Talling and Inglis (2009) would require irreversible unconsciousness within 30 seconds, and if traps met this standard, this would produce a Part B score of D and total score of 1D.<br><br>Moles may be trapped year-round. Trapping during the breeding season would have welfare impacts for dependent young, in which case efforts should be made to find any nests and humanely despatch dependent young.

Bibliography

AIHTS (1998) *AGREEMENT on international humane trapping standards between the European Community, Canada and the Russian Federation)* http://www.canadainternational.gc.ca/eu-ue/assets/pdfs/eu25-en.pdf.

Atkinson, R.P.D., Macdonald, D.W. and Johnson, P.J. (1994) The status of the European mole *Talpa europaea* L. as an agricultural pest and its management. *Mammal Review* 24: 73-90.

Baker, S.E., Ellwood, S.A., Tagarielli, V.L. and Macdonald, D.W. (2012) Mechanical Performance of Rat, Mouse and Mole Spring Traps, and Possible Implications for Welfare Performance. *PLoS ONE* , 7 (6): e39334. doi:10.1371/journal.pone.0039334.

Baker, S.E., Shaw, R.F., Atkinson, R.P.D., West, P. and Macdonald, D.W. (2015) Potential welfare impacts of kill-trapping European moles (*Talpa europaea*) using scissor traps and Duffus traps: a post-mortem examination study. *Animal Welfare* , 24: 1-14.

Falk, J.L., O'Brien, J.F. and Kerr, R. (1992) Fluid resuscitation in traumatic hemorrhagic shock. *Critical Care Clinics,* 8 (2): 323-40.

Fera (2009) *Review of red squirrel conservation activity in northern England* . A report to Natural England (NECR019). Natural England. http://www.forestry.gov.uk/pdf/eng-nee-redsquirrel-review-09.pdf/$FILE/eng-nee-redsquirrel-review-09.pdf.

Gorman, M.L. and Stone, R.D. (1990) *The Natural History of Moles* . Christopher Helm: London, UK.

Martel, M.J., MacKinnon, C.J., Arsenault, M.Y., Bartellas, E., Klein, M.C., Lane, C.A., Martel, M.J., Sprague, A.E. and Wilson, A.K. (2002) Hemorrhagic shock. *Journal of Obstetrics and Gynaecology Canada* , 24(6): 504-511. http://www.sogc.org/guidelines/public/115E-CPG-June2002.pdf.

Smith, H.O. (1997) Shock in the gynecologic patient. In: Rock JA,Thomson JD, editors. *Telinde's Operative Gynecology* . 8th ed. Lippincott-Raven, Philadelphia, US. pp. 245-61.

Talling, J.C. and Inglis, I.R. (2009) *Improvements to trapping standards* . DG ENV, 361 pp. http://ec.europa.eu/environment/biodiversity/animal_welfare/hts/pdf/final_report.pdf.

Xu, H., Han, L.C., Gao, W., Sun, X.X., Zhou, Y., Meng, X.Z., Zhang, H. and Xu, L.X. (2011) Therapeutic effects of intravenous infusion of hyper-oxygenated solution on acute haemorrhagic shock in rabbits. *Journal of International Medical Research* , 39: 1843-1851.
