# Supplementary material for: Assessing Animal Welfare Impacts in the Management of European Rabbits (Oryctolagus cuniculus), European Moles (Talpa europaea) and Carrion Crows (Corvus corone)
Source: PLoS One. 2016 Jan 4;11(1):e0146298. doi: 10.1371/journal.pone.0146298 (PMC4699632; doi:10.1371/journal.pone.0146298)
Supplement: S15 Table — (PDF) [file pone.0146298.s023.pdf]

Control method:	**Live-trapping moles**

Assumptions	Best practice is followed in accordance with the Standard Operating Procedure S4.<br><br>Moles are trapped using plastic tube traps.<br><br>Trapping is conducted in spring, but not during breeding period.<br><br>Traps are checked every 4 hours.

PART A: assessment of overall welfare impact

DOMAIN 1 Water or food restriction, malnutrition				
No impact	Mild impact	Moderate impact	Severe impact	Extreme impact

DOMAIN 2 Environmental challenge				
No impact	Mild impact	Moderate impact	Severe impact	Extreme impact

DOMAIN 3 Disease, injury, functional impairment				
No impact	Mild impact	Moderate impact	Severe impact	Extreme impact

DOMAIN 4 Behavioural or interactive restriction				
No impact	Mild impact	Moderate impact	Severe impact	Extreme impact

DOMAIN 5 Anxiety, fear, pain, distress, thirst, hunger				
No impact	Mild impact	Moderate impact	Severe impact	Extreme impact

Overall impact
Moderate-Severe impact

DURATION OF IMPACT				
Immediate to seconds	Minutes	Hours	Days	Weeks

SCORE FOR PART A:	**5-6**
Summary of evidence	
Domain 1	Moles have a high metabolic rate and may die if left in a trap for any length of time without food (Natural England, 2011). Trapped moles will be in the trap for up to four hours, but bait provided in the trap should be sufficient to prevent anything more than minor impact on food or water intake, as sufficient worms are provided for a 24 hour period and worms provide a source of moisture too (Rudge, 1966). There should be no impact on nutrition in this time.
Domain 2	Because tube traps do not accommodate the provision of bedding, moles might quickly suffer chilling in cold weather (Baker & Macdonald, 2012). Little is known of the welfare impacts of live-trapping moles, but in one study, two out of eight captures were found dead in Friesian traps provisioned with bedding and food; both had been caught overnight and were discovered dead in the morning after ≤14 hours in the trap (Ros Shaw, Pers. Comm.).
Domain 3	No impact in this domain.
Domain 4	Tube traps allow little movement because they are narrow. Normal behaviour and interactions will be prevented while the mole is in the trap. The one-way swing doors at either end of the trap potentially allow more than one mole to become trapped together; this may lead to fighting which could cause injury or death (Baker & Macdonald, 2012).
Domain 5	Moles are insectivores and as such have high metabolic rates, and live-traps need to be checked frequently to prevent them dying of cold, wet or stress (Gorman & Stone, 1990). While held in a trap moles may become agitated from not being able to perform their natural behaviour. The process of transferring a mole from trap to bucket for transport to the release site will involve minimal handling.

PART B: assessment of mode of death -	
**Not performed - non-lethal method**	

Summary

CONTROL METHOD	**Live-trapping moles**	
OVERALL HUMANENESS SCORE	**5-6**	
Comments	Moles may sometimes detect a trap that has been set in their run and block it with soil before tunnelling around it (Nicholls, 2010). This is likely to affect only a minority of trap-settings and to have no more impact on moles than when moles themselves conduct routine maintenance to damaged tunnels. A range of impacts has been specified for domains 2, 4 and 5, because of a lack of objective data on these. The score for Part A may be greater (worse) if the traps are checked less frequently, largely through impacts in domains 1, 2 and hence 5.<br><br>Tube-traps have a one-way swing door at either end which could potentially allow more than one animal to enter the trap and fight with each other (Baker & Macdonald, 2012). Moles have largely exclusive territories outside the breeding season (e.g. 13% range overlap, Macdonald et al., 1994) and so this may not occur frequently in most situations, during most of the year, but might occur more frequently during the breeding season.	

Bibliography

Baker, S.E. and Macdonald, D.W. (2012) Not so humane mole tube traps. *Animal Welfare* , 21(4): 613-615.

Gorman, M.L. and Stone, R.D. (1990) *The Natural History of Moles* . Christopher Helm, Kent, UK.

Natural England (2011) *Moles: options for management and control* . Natural England Technical Information Note TIN033, http://publications.naturalengland.org.uk/publication/34015?category=23035.

Nicholls, J. (2010) *Mole catching; a practical guide.* Crowood Press, Marlborough, UK.

Rudge, A.J.B. (1966) catching and keeping live moles. *Journal of Zoology* , 149(1): 42-45.
