# Supplementary material for: Assessing Animal Welfare Impacts in the Management of European Rabbits (Oryctolagus cuniculus), European Moles (Talpa europaea) and Carrion Crows (Corvus corone)
Source: PLoS One. 2016 Jan 4;11(1):e0146298. doi: 10.1371/journal.pone.0146298 (PMC4699632; doi:10.1371/journal.pone.0146298)
Supplement: S16 Table — (PDF) [file pone.0146298.s024.pdf]

**Control method:**	**Relocating moles**

Assumptions	Best practice is followed in accordance with the Standard Operating Procedure S4.<br><br>Moles are released into soft-release chambers.<br><br>Release is conducted in the spring, but not during the breeding period.

PART A: assessment of overall welfare impact

DOMAIN 1 Water or food restriction, malnutrition				
No impact	Mild impact	Moderate impact	Severe impact	Extreme impact

DOMAIN 2 Environmental challenge				
No impact	Mild impact	Moderate impact	Severe impact	Extreme impact

DOMAIN 3 Disease, injury, functional impairment				
No impact	Mild impact	Moderate impact	Severe impact	Extreme impact

DOMAIN 4 Behavioural or interactive restriction				
No impact	Mild impact	Moderate impact	Severe impact	Extreme impact

DOMAIN 5 Anxiety, fear, pain, distress, thirst, hunger				
No impact	Mild impact	Moderate impact	Severe impact	Extreme impact

Overall impact
Moderate-Extreme impact

DURATION OF IMPACT				
Immediate to seconds	Minutes	Hours	Days	Weeks

SCORE FOR PART A:	**6-8**
Summary of evidence	
Domain 1	The quantification of availability of food resources, suitable soil type and the presence of conspecifics can be difficult (see Massei et al., 2010). There is no conclusive evidence that relocated moles can successfully set up a new territory at a release site without an existing run system (Natural England, 2011); this is likely to exert considerable pressure on the mole to obtain sufficient food to survive. In order to survive, a relocated mole will need to create an entire feeding tunnel system, capable of meeting its food requirements, before it succumbs to starvation in the process. Whether or not it does this is likely to depend on whether it has been released in suitable, unoccupied habitat, whether it is in good condition following trapping (see Massei et al., 2010) and whether the soft-release facilities are sufficient to sustain the mole until the new tunnel system can take over. Setting up a new territory may be within the mole's normal ability, because young moles disperse from the natal territory to set up their own territory from scratch. However, once established in a territory, a mole tends to stay there for the rest of its life (Gorman & Stone, 1990). It may be that moles cope less well with the challenge of establishing a new territory when they are mature or when they are moved suddenly to a completely new area.
Domain 2	Provided moles have access to food, dry hay, adequate ventilation and a cool temperature they should be relatively easy to keep alive (Rudge, 1966). Relocated moles released into suitable soft-release chambers, with access to suitable habitat, should not be subject to environmental conditions outside the normal range encountered.
Domain 3	If a relocated mole is forced to disperse above ground because it finds itself in unsuitable or occupied habitat, it will be extremely vulnerable to predation by a variety of mammals, and birds such as owls. This has been observed among dispersing young moles (Gorman & Stone, 1990) and moles forced above ground when the soil is frozen or too dry and hard for digging (Morris, 1966). Moles are highly territorial and release into another mole's territory (or being forced to enter one in search of suitable habitat) may result in fighting between individuals (Natural England, 2011) and potentially death (Atkinson & Macdonald, 1994).
Domain 4	Moles will be subject to some behavioural and interactive restriction while being transported to the release site, but once released into the soft-release chamber may not be unnaturally restricted in this way.
Domain 5	The process of transferring a mole from trap to release site will involve minimal handling and transportation in a bucket provisioned with bedding and food. However, the process of being relocated to a new site with no tunnel system is likely to be stressful.

PART B: assessment of mode of death -
**Not performed - non-lethal method**

Summary

CONTROL METHOD	**Relocating moles**	
OVERALL HUMANENESS SCORE	**6-8**	
Comments	The biology and behaviour of moles are not well-adapted to live-trapping and relocation (Baker & Macdonald, 2012). However, the assessment assumes that best efforts will be made to select suitable, unoccupied habitat for the release site. However, little is known about selecting suitable habitat beyond trying to ensure there is access to sufficient food. Natural England does not recommend that moles are relocated, on welfare grounds, because of the risk of fighting with resident moles and the pressure exerted on a mole to set up a new territory (Natural England, 2011).<br><br>Doubt about the fate of relocated moles produces impact estimates up to extreme levels in Domains 1 and 3.<br><br>Relocating a mole may possibly constitute an offence under the Animal Welfare Act 2006 (Natural England, 2011).<br><br>If successful establishment of a territory in suitable, unoccupied habitat could be assumed within a few days then the impact in Domain 1 would be reduced to Moderate and that in Domain 3 to No Impact, making that in Domain 5, and the overall impact, Moderate. The overall score for relocation would then be 6.<br><br>The effects of live-trapping and relocation will be compounded when performed consecutively.	

Bibliography

Atkinson, R.P.D. and Macdonald, D.W. 1994. Can repellents function as a nonlethal means of controlling moles (*Talpa europaea*). *Journal of Applied Ecology* 31: 731-736.

Baker, S.E. and Macdonald, D.W. (2012) Not so humane mole tube traps. *Animal Welfare*, 21 (4): 613-615.

Gorman, M.L. and Stone, R.D. (1990) *The Natural History of Moles*. Christopher Helm, Kent, UK.

Massei, G., Quy, R.J., Gurney, J. and Cowan, D.P. (2010) Can translocations be used to mitigate human-wildlife conflicts? *Wildlife Research*, 37: 428-439.

Morris, P. (1966) The mole as a surface dweller. *Journal of Zoology*, 149 (1): 46-49.

Natural England (2011) *Moles: options for management and control*. Natural England Technical Information Note TIN033, http://publications.naturalengland.org.uk/publication/34015?category=23035.

Rudge, A.J.B. (1966) catching and keeping live moles. *Journal of Zoology*, 149 (1): 42-45.
