# Supplementary material for: Assessing Animal Welfare Impacts in the Management of European Rabbits (Oryctolagus cuniculus), European Moles (Talpa europaea) and Carrion Crows (Corvus corone)
Source: PLoS One. 2016 Jan 4;11(1):e0146298. doi: 10.1371/journal.pone.0146298 (PMC4699632; doi:10.1371/journal.pone.0146298)
Supplement: S17 Table — (PDF) [file pone.0146298.s025.pdf]

Control method:	**Shooting crows**

Assumptions	Best practice is followed in accordance with the Standard Operating Procedure S6.<br><br>Shooter uses appropriate shotgun and ammunition, is competent, and judges shot placement and range accurately. For crows the preferred method is a shot to the centre of the chest with a shotgun.<br><br>Wounding rates should be low if best practice is followed (but it is recognised that crows are often shot by non-professionals and with air weapons).<br><br>A single bird is targeted at a time.<br><br>Shooting is conducted during daylight hours.<br><br>Shooting takes place under Natural England General Licence WML-GL04 [96]. Under this licence, shooting may only be conducted where the authorised person is satisfied that appropriate legal methods of resolving the problem such as scaring and proofing are either ineffective or impracticable. Shooting may legally be conducted during the breeding period but this period is not covered by this assessment.<br><br>Shooting may have limited value in bird control, but may be useful as part of wider management effort, e.g. as part of a scaring approach.<br><br>The impacts in Part A of the assessment were considered for a number of birds feeding together. The first bird would be naïve but the impact would increase for each successive bird shot in a single shooting exercise.<br><br>Crows are territorial during breeding, so shooting may not be very efficient then. However if shooting is conducted during breeding, efforts should be made to find and destroy eggs or young.

PART A: assessment of overall welfare impact

DOMAIN 1 Water or food restriction, malnutrition				
No impact	Mild impact	Moderate impact	Severe impact	Extreme impact

DOMAIN 2 Environmental challenge				
No impact	Mild impact	Moderate impact	Severe impact	Extreme impact

DOMAIN 3 Disease, injury, functional impairment				
No impact	Mild impact	Moderate impact	Severe impact	Extreme impact

DOMAIN 4 Behavioural or interactive restriction				
No impact	Mild impact	Moderate impact	Severe impact	Extreme impact

DOMAIN 5 Anxiety, fear, pain, distress, thirst, hunger				
No impact	Mild impact	Moderate impact	Severe impact	Extreme impact

Overall impact
Mild impact

DURATION OF IMPACT				
Immediate to seconds	Minutes	Hours	Days	Weeks

SCORE FOR PART A:	**3**
Summary of evidence	
Domain 1	No impact in this domain.
Domain 2	No impact in this domain.
Domain 3	No impact in this domain.
Domain 4	No impact in this domain.
Domain 5	Crows may suffer fear or distress for a short time before being shot if another bird in the flock is shot first. In such cases, crows may retreat to a nearby tree, in the meantime, exhibiting natural 'flight or fight' stress response as when encountering a predator. These endocrine responses are short-term and stress hormone levels return to normal quickly (Munck et al., 1984). Birds which have retreated when frightened by the sound of a shot may subsequently return to the feeding area and be shot. Therefore when a particular bird is shot it may have suffered a mild impact for a few minutes beforehand.

PART B: assessment of mode of death -	**Shooting crows**

Time to insensibility (minus any lag time)				
Immediate to seconds	Minutes	Hours	Days	Weeks
Level of suffering (after application of the method that causes death but before insensibility)				
No suffering	Mild suffering	Moderate suffering	Severe suffering	Extreme suffering

SCORE FOR PART B:	**A**
Summary of evidence	
Duration	The major factor in wounding capacity using a shotgun with a given load is the range at which the shot is taken (Noer et al., 2007; Wilson, 1978). Shooting from an effective range will result in a denser shot pattern, killing the bird quickly. Death is caused by damage to vital organs, haemorrhaging and hydrostatic shock. Some birds may be wounded and will require follow-up. Time until death for these birds could be minutes.
Suffering	The lethality of a shotgun wound depends on the number of shotgun pellets entering the body, the organs injured and the amount of tissue damage (Merck, 2007). A well-placed shot, taken from effective range, should render a crow unconscious immediately and it should not suffer. Any wounded birds requiring follow-up may experience moderate suffering.

Summary

CONTROL METHOD	**Shooting crows**
OVERALL HUMANENESS SCORE	**3A**
Comments	The mode of death for birds wounded by shooting and not followed up could score D.<br><br>Wounding rates - this assessment assumed that crows were shot according to best practice, i.e. with the appropriate weapon and ammunition, from a suitable distance, and the shot accurately placed. However, studies with foxes have suggested that a proportion of the foxes shot at are wounded. One study, based on x-ray evidence of shooting wounds among foxes admitted to wildlife hospitals and vets, estimated wounding rates to be 9% with shotguns and 3% with rifles (Bentley et al. cited in Baker et al., 2006). Another study examined the accuracy of shooting, by shooters of varying skill levels, at life-size paper fox targets, and estimated that wounding rates could be considerably greater (Fox et al., 2005).<br><br>Hydrostatic shock - when an animal is shot its organs can be damaged both by the projectile and by the pressure wave or hydrostatic shock produced when the projectile enters the body (Courtney & Courtney, 2008). In some cases the pressure wave produced may be of sufficient pressure to kill an animal before it dies of blood loss effects (Courtenay & Courtenay, 2007).<br><br>Individual birds that are part of a population that is managed by shooting, but which are not themselves shot, may suffer 'non-target' Part A effects over time.

Bibliography

Baker, P., Harris, S. and White, P. (2006) *After the hunt. The future for foxes in Britain*. London, UK. http://www.thefoxwebsite.org/After-the-Hunt.pdf: International Fund for Animal Welfare (IFAW).

Courtney, A. and Courtney, M. (2007) Links between traumatic brain injury and ballistic pressure waves originating in the thoracic cavity and extremities. *Brain Injury,* 21: 657-662.

Courtney, M. and Courtney, A. (2008) *Scientific Evidence for Hydrostatic Shock*. 0803.3051 at <http://arxiv.org/abs/0803.3051>.

Fox, N.C., Blay, N., Greenwood, A.G., Wise, D. and Potapov, E. (2005) Wounding rates in shooting foxes (*Vulpes vulpes*). *Animal Welfare,* 14: 93-102.

Merck, M.D. (2007) *Veterinary forensics: animal cruelty investigations*. Iowa: Blackwell Publishers.

Munck, A., Guyre, P. and Holbrook, N. (1984). Physiological Functions of Glucocorticoids in Stress and Their Relation to Pharmacological Actions. *Endocrine Reviews*, 5: 25-44.

Noer, H., Madsen, J. and Hartmann, P. (2007) Reducing wounding of game by shotgun hunting: effects of a Danish action plan on pink-footed geese. *Journal of Applied Ecology*, 44(3):653-62.

Sharp, T. and Saunders, G. (2009) *Shooting of pest birds (small to medium); welfare assessment*. http://www.feral.org.au/wp-content/uploads/2011/04/bird_shooting_sm_med_worksheet.pdf.

Wilson, J.M. (1978). Shotgun ballistics and shotgun injuries. *Western Journal of Medicine,* 129(2): 149-55.
