# Supplementary material for: Assessing Animal Welfare Impacts in the Management of European Rabbits (Oryctolagus cuniculus), European Moles (Talpa europaea) and Carrion Crows (Corvus corone)
Source: PLoS One. 2016 Jan 4;11(1):e0146298. doi: 10.1371/journal.pone.0146298 (PMC4699632; doi:10.1371/journal.pone.0146298)
Supplement: S18 Table — (PDF) [file pone.0146298.s026.pdf]

**Control method:**	**Scaring crows using gas guns**

Assumptions	Best practice is followed in accordance with the standard operating procedure S8.<br><br>This SOP is for two months starting after harvest and just before the winter wheat crop is planted in September-October, to prevent crows taking newly sewn crop seed and seedlings.<br><br>This assessment does not include the breeding period.<br><br>Scaring takes place only during daylight hours. Scaring may be combined with some shooting to reinforce aversion but the effects of shooting are not included in this SOP.

PART A: assessment of overall welfare impact

DOMAIN 1 Water or food restriction, malnutrition				
No impact	Mild impact	Moderate impact	Severe impact	Extreme impact

DOMAIN 2 Environmental challenge				
No impact	Mild impact	Moderate impact	Severe impact	Extreme impact

DOMAIN 3 Disease, injury, functional impairment				
No impact	Mild impact	Moderate impact	Severe impact	Extreme impact

DOMAIN 4 Behavioural or interactive restriction				
No impact	Mild impact	Moderate impact	Severe impact	Extreme impact

DOMAIN 5 Anxiety, fear, pain, distress, thirst, hunger				
No impact	Mild impact	Moderate impact	Severe impact	Extreme impact

Overall impact
Mild impact

DURATION OF IMPACT				
Immediate to seconds	Minutes	Hours	Days	Weeks

SCORE FOR PART A:	5
Summary of evidence	
Domain 1	Depending on the scaring effect of a gas gun, birds might lose valuable time and energy by continuously approaching the protected field, and then retreating when the gas gun fires. However, if birds learn quickly to avoid the protected site, this effect will be short-term only (a few days) and of minimal impact. If alternative food is in short supply, an effective bird scarer could potentially restrict the food available to birds, or cause them to move on to another site further afield.
Domain 2	Noise from intermittent gunfire may provide environmental challenge if birds are roosting very close to the protected site. This could potentially cause them to roost elsewhere. However, it is unlikely that roosts will be very close to the gun, as guns tend to be deployed a distance into the crop (in the centre of an area of crop to be protected), and so birds may habituate quickly.
Domain 3	Gas guns produce extremely loud noise levels, which at close quarters would be considered damaging to human hearing. However, birds are unlikely to be in the immediate vicinity of the gas gun when it is first deployed because some corvids are known to display neophobia in a feeding context (Zucca et al., 2007) and noise levels further from the gun are likely to have a mild, reversible impact on birds' hearing, e.g. through temporary threshold shift (Govindarajua et al., 2011).
Domain 4	Birds will exhibit a natural 'flight or fight' stress response as when encountering a predator. These endocrine responses are short-term and stress hormone levels quickly return to normal (Munck et al., 1984).
Domain 5	Birds will be startled by gas guns but will recover quickly as described above. While scarers induce some degree of stress, stress levels are acceptable because they do not exceed those that a bird would experience in a variety of natural situations and the bird is free to take appropriate avoidance action (Inglis, 1985).

PART B: assessment of mode of death -
**Not performed - non-lethal method**

Summary

CONTROL METHOD	**Scaring crows using gas guns**	
OVERALL HUMANENESS SCORE		5
Comments	If alternative food is not available close by, this could increase the impact in Domain 1 and potentially 5 - and hence the overall impact.<br><br>If some shooting is conducted to reinforce aversion this would need to be considered in a separate assessment.<br><br>If scaring occurred in spring, during the breeding period, there is potential for a gas gun to disturb nesting in the same way as roosting, producing a greater impact in domain 2. If birds are nesting very close to the gun, this could increase the impact experienced in Domains 2, 4 and hence 5. There is a risk that nests and nestlings might be abandoned if disturbance is great.	

Bibliography

Govindarajua, R., Omar, R., Rajagopalan, R., Norlisah, R. and Ng, K.H. (2011) Hearing loss after noise exposure. *Auris Nasus Larynx* , 38 (4): 519–522.

Inglis, I.R. (1985) Humane control of birds. In: *Humane Control of Land Mammals and Birds* . Universities Federation for Animal Welfare (UFAW), Potters Bar, England.

Munck, A., Guyre, P. and Holbrook, N. (1984). Physiological Functions of Glucocorticoids in Stress and Their Relation to Pharmacological Actions. *Endocrine Reviews,* 5: 25-44.

Zucca, P., Milos, N. and Vallortigara, G. (2007) Piagetian object permanence and its development in Eurasian jays (*Garrulus glandarius*). *Animal Cognition* , 10: 243–259.
