# Supplementary material for: Assessing Animal Welfare Impacts in the Management of European Rabbits (Oryctolagus cuniculus), European Moles (Talpa europaea) and Carrion Crows (Corvus corone)
Source: PLoS One. 2016 Jan 4;11(1):e0146298. doi: 10.1371/journal.pone.0146298 (PMC4699632; doi:10.1371/journal.pone.0146298)
Supplement: S19 Table — (PDF) [file pone.0146298.s027.pdf]

**Control method:**	**Cage-trapping of crows & cervical dislocation**

Assumptions	Best practice is followed in accordance with S7.<br><br>Trapping takes place under Natural England General Licence WML-GL04 (Natural England 2011). Under this licence, trapping may only be conducted where the authorised person is satisfied that appropriate legal methods of resolving the problem such as scaring and proofing are either ineffective or impracticable. Trapping may be conducted during the breeding period but this period is not covered by this assessment. Trapping may have limited value in bird control, but may be useful as part of wider management effort, e.g. as part of a scaring approach.<br><br>This assessment covers use of single-capture Larsen traps, checked every 24 hours. The assessment is for the trapped bird and does not consider impacts on the decoy.<br><br>Note that handling will reduce the humaneness of this method.

PART A: assessment of overall welfare impact

DOMAIN 1 Water or food restriction, malnutrition				
No impact	**Mild impact**	Moderate impact	Severe impact	Extreme impact

DOMAIN 2 Environmental challenge				
No impact	**Mild impact**	Moderate impact	Severe impact	Extreme impact

DOMAIN 3 Disease, injury, functional impairment				
No impact	**Mild impact**	Moderate impact	Severe impact	Extreme impact

DOMAIN 4 Behavioural or interactive restriction				
No impact	Mild impact	**Moderate impact**	Severe impact	Extreme impact

DOMAIN 5 Anxiety, fear, pain, distress, thirst, hunger				
No impact	Mild impact	**Moderate impact**	Severe impact	Extreme impact

Overall impact
**Moderate impact**

DURATION OF IMPACT				
Immediate to seconds	Minutes	**Hours**	Days	Weeks

SCORE FOR PART A:	**5**
Summary of evidence	
Domain 1	Food and water are provided and replenished daily. Wild-caught crows can be readily sustained in captivity (e.g. Cox et al., 2004), but some corvids display neophobia in a feeding context (Zucca et al., 2007), and may not feed or drink normally for a short while following capture. However, intake restrictions are likely to be within usual tolerances for the species.
Domain 2	Trapping is avoided in adverse conditions and sufficient shade and protection from unexpected adverse weather should be provided. However, a captured bird may be exposed to damp, cold or hot conditions, depending on trap location.
Domain 3	Birds may be injured, e.g. on wings or the head, if they try to escape when humans approach the trap. Inspecting the trap (and removing any bird that has been captured) shortly before nightfall will minimise the risk of nocturnal predators, such as badgers or foxes, injuring birds caught during the day.
Domain 4	Under General Licence WML-GL04, cage dimensions do not need to satisfy the requirements of section 8(1) of the Wildlife and Countryside Act (1981). Normal behaviour and movement is restricted by the cage. Trapped birds are likely to suffer from distress when confined and they can sometimes be injured while trying to escape from the trap or during capture or restraint prior to euthanasia. Raptors may sit on the trap, causing fear and panic.
Domain 5	Birds are likely to experience fear and distress while trapped, and especially during handling. American crows *(Corvus brachyrhynchos)* are known to discriminate among individual people and to 'scold', with harsh vocalisations, people who have threatened them in the past (Marzluff et al., 2010). They also transmit such information socially, to conspecifics (Cornell et al., 2012). Trapped crows might therefore become additionally stressed when a trapper familiar to the decoy bird approaches, as the decoy bird may scold the trapper from a distance, potentially adding to the intensity or duration of suffering by trapped birds.

PART B: assessment of mode of death -	**Cervical dislocation**

Time to insensibility (minus any lag time)				
Immediate to seconds	Minutes	Hours	Days	Weeks
Level of suffering (after application of the method that causes death but before insensibility)				
No suffering	Mild suffering	Moderate suffering	Severe suffering	Extreme suffering

SCORE FOR PART B:	**C**
Summary of evidence	
Duration	Cervical dislocation does not cause concusssion and so the bird may not become unconscious immediately (Gregory & Wotton, 1990; Erasmus et al., 2010). Studies on chickens indicate that electrical activity in the brain can continue for 13 seconds after cervical dislocation (Gregory & Wotton, 1990).
Suffering	Birds will be handled prior to cervical dislocation and will suffer fear and distress as a result. Crows may be additionally stressed during handling because of scolding by a decoy bird that recognises the trapper as threatening (Marzluff et al., 2010) or by social learning about the trapper (Cornell et al., 2012). In a study of cervical dislocation of turkeys, reflexes lasted for 43 seconds following cervical dislocation, with birds gasping as a result of hypoxia and most likely distressed before becoming unconscious (Erasmus et al., 2010). To make sure that unconsciousness occurs as quickly as possible, the operator needs first to master the technical skills of cervical dislocation. Death should be confirmed and a second mode of death employed if necessary.

Summary

CONTROL METHOD	**Cage-trapping of crows and cervical dislocation**
OVERALL HUMANENESS SCORE	**5C**
Comments	Crows are intelligent birds, potentially prone to stress.<br><br>There will be welfare impacts on the decoy bird which will depend on the length of time for which it is used before being replaced.<br><br>During the breeding season, there may be additional impacts for parent birds held in the trap, as well as impacts for dependent offspring. If trapping is conducted during breeding, dependent offspring should be found and despatched humanely.<br><br>This assessment assumes that the SOP is followed but if traps are checked less often than specified, or trapped birds not despatched quickly after discovery, then impacts could be increased.

Bibliography

Cornell, H.N., Marzluff, J.M. and Pecoraro, S. (2012) Social learning spreads knowledge about dangerous humans among crows. *Proceedings of the Royal Society B - Biological Sciences* , 279(1728): 499-508.

Cox, R., Baker, S.E., Macdonald, D.W. and Berdoy, M. (2004) Protecting egg prey from Carrion Crows: the potential of aversive conditioning. *Applied Animal Behaviour Science* , 87(3-4): 325-342.

Erasmus, M.A., Lawlis, P., Duncan, I.J.H. and Widowski, T.M. (2010). Using time to insensibility and estimated time of death to evaluate a non-penetrating captive bolt, cervical dislocation, and blunt trauma for on-farm killing of turkeys. *Poultry Science* , 89: 1345-1354.

Gregory, N.G. and Wotton, S.B. (1990) Comparison of neck dislocation and percussion of the head on visual evoked responses in the chicken's brain. *Veterinary Record* , 126: 570-572.

Marzluff, J.M., Walls, J., Cornell, H.N., Withey, J.C. and Craig, D.P. (2010) Lasting recognition of threatening people by wild American crows. *Animal Behaviour* , 79(3): 699-707.

Natural England (2013) *LICENCE (General) WML-GL04 To kill or take certain wild birds to prevent serious damage or disease* . Natural
